# Fifty million years of beetle evolution along the Antarctic Polar Front

**DOI:** 10.1073/pnas.2017384118

**Published:** 2021-06-09

**Authors:** Helena P. Baird, Seunggwan Shin, Rolf G. Oberprieler, Maurice Hullé, Philippe Vernon, Katherine L. Moon, Richard H. Adams, Duane D. McKenna, Steven L. Chown

**Affiliations:** ^a^School of Biological Sciences, Monash University, Clayton, VIC 3800, Australia;; ^b^Department of Biological Sciences, University of Memphis, Memphis, TN 38152;; ^c^Center for Biodiversity Research, University of Memphis, Memphis, TN 38152;; ^d^School of Biological Sciences, Seoul National University, Seoul 08826, Republic of Korea;; ^e^Australian National Insect Collection, Commonwealth Scientific and Industrial Research Organisation, Canberra, ACT 2601, Australia;; ^f^Institut de Génétique, Environnement et Protection des Plantes, Institut national de recherche pour l’agriculture, l’alimentation et l’environnement, Université de Rennes, 35653 Le Rheu, France;; ^g^Université de Rennes, CNRS, UMR 6553 ECOBIO, Station Biologique, 35380 Paimpont, France;; ^h^Department of Computer and Electrical Engineering and Computer Science, Florida Atlantic University, Boca Raton, FL 33431;; ^i^Securing Antarctica’s Environmental Future, School of Biological Sciences, Monash University, Clayton, VIC 3800, Australia

**Keywords:** Antarctica, species radiation, paleoclimate, herbivory, island biogeography

## Abstract

Global cooling and glacial–interglacial cycles since Antarctica’s isolation have been responsible for the diversification of the region’s marine fauna. By contrast, these same Earth system processes are thought to have played little role terrestrially, other than driving widespread extinctions. Here, we show that on islands along the Antarctic Polar Front, paleoclimatic processes have been key to diversification of one of the world’s most geographically isolated and unique groups of herbivorous beetles—Ectemnorhinini weevils. Combining phylogenomic, phylogenetic, and phylogeographic approaches, we demonstrate that these weevils colonized the sub-Antarctic islands from Africa at least 50 Ma ago and repeatedly dispersed among them. As the climate cooled from the mid-Miocene, diversification of the beetles accelerated, resulting in two species-rich clades. One of these clades specialized to feed on cryptogams, typical of the polar habitats that came to prevail under Miocene conditions yet remarkable as a food source for any beetle. This clade’s most unusual representative is a marine weevil currently undergoing further speciation. The other clade retained the more common weevil habit of feeding on angiosperms, which likely survived glaciation in isolated refugia. Diversification of Ectemnorhinini weevils occurred in synchrony with many other Antarctic radiations, including penguins and notothenioid fishes, and coincided with major environmental changes. Our results thus indicate that geo-climatically driven diversification has progressed similarly for Antarctic marine and terrestrial organisms since the Miocene, potentially constituting a general biodiversity paradigm that should be sought broadly for the region’s taxa.

## Significance

The Antarctic environment is famously inhospitable to most terrestrial biodiversity, traditionally viewed as a driver of species extinction. Combining population- and species-level molecular data, we show that beetles on islands along the Antarctic Polar Front diversified in response to major climatic events over the last 50 Ma in surprising synchrony with the region’s marine organisms. Unique algae- and moss-feeding habits enabled beetles to capitalize on cooling conditions, which resulted in a decline in flowering plants—the typical hosts for beetles elsewhere. Antarctica’s cooling paleoclimate thus fostered the diversification of both terrestrial and marine life. Climatically driven evolutionary processes since the Miocene may underpin much of the region’s diversity, are still ongoing, and should be further investigated among Antarctic biota.

Antarctica’s isolation, cooling, and glacial–interglacial cycles over the Cenozoic have resulted in the remarkable diversification of a unique marine fauna (1, 2). The investigation of marine radiations in Antarctica has reshaped modern understanding of biodiversity processes, for example, by revealing a surprising inverse latitudinal gradient in diversification rates for fish and brittle stars (3–5). In contrast, Antarctica’s paleoclimatic legacy for terrestrial communities has long been considered one of widespread extinction due to glaciation. Evidence of terrestrial species surviving in Antarctic glacial refugia (6) and discoveries of substantial endemic diversity and biogeographic structuring in some groups (7, 8) is changing this narrative, indicating extended evolutionary histories on land. Yet, such evolutionary histories remain obscured by a lack of large-scale molecular phylogenetic work, with most Antarctic terrestrial research focused on small subsets of species or populations (9, 10). The few studies that have taken a multilocus phylogenetic approach have uncovered hidden terrestrial diversity and signals of long-term allopatric divergence (e.g., refs. 11 and 12), hinting that Cenozoic climatic processes may have driven terrestrial diversification in ways similar to that for marine life.

The hypothesis that diversification has proceeded similarly in Antarctic marine and terrestrial groups has not been tested. While the extinction of a diverse continental Antarctic biota is well established (13), mounting evidence of significant and biogeographically structured Antarctic terrestrial diversity (8, 14, 15) with a long evolutionary history (6, 16) suggests the possibility of broadly similar diversification processes across marine and terrestrial Antarctic systems. If valid for some taxa, further tests should then be sought across a wider variety of organisms. Here, we therefore evaluate the terrestrial applicability of the paradigm emerging for Antarctic marine biodiversity—that a major cooling phase from the mid-Miocene climatic transition (14 Ma) onwards, and subsequent habitat restructuring, have led to significant and ongoing diversification for many taxa, including those with much older origins in the region (2, 4, 17). We do so by using one of the most well-known and speciose groups from the sub-Antarctic, the herbivorous Ectemnorhinini weevils (Coleoptera: Curculionidae) (18–20).

Preliminary molecular studies indicate that the Ectemnorhinini, along with numerous other terrestrial taxa, have long histories in the sub-Antarctic, extending to the Miocene or earlier [e.g., beetles (21), midges (22), and springtails (11)]. This enables a comparison of their evolution throughout the same periods of environmental change that drove the diversification of Antarctic marine taxa. Moreover, the sub-Antarctic islands overlap spatially with the Southern Ocean, with climates that reflect oceanic conditions both past and present (23, 24). While in some respects quite different to the continental Antarctic, the islands are in other ways quite similar, providing a window into diversification processes that might be sought for continental groups, especially given their age and biogeographic structuring. Both regions share many higher taxa (e.g., ref. 25), a dynamic geo-climatic history (6, 26), a profound degree of isolation, and indications that climatic events likely structured their biota (6, 8, 27). The terrestrial habitat on the continent and its surrounding islands is fragmented by large expanses of ice or ocean, respectively, and has been further isolated by the Antarctic Circumpolar Current for at least 34 Ma (28, 29). Cyclic growth and contraction of ice sheets throughout the Plio–Pleistocene, though typically associated with the continent, has also had extensive impacts on the sub-Antarctic islands (26). The more intensively surveyed sub-Antarctic faunas thus provide an opportunity to investigate terrestrial diversification processes for the wider Antarctic while recognizing that for many groups on the continent, the main legacy of change has been extinction.

To test the hypothesis that a major phase of cooling from the mid-Miocene onwards and subsequent habitat restructuring has led to the diversification of Antarctic terrestrial taxa, we integrate three tiers of molecular data to reveal a comprehensive evolutionary history for the Ectemnorhinini weevils. This additionally allows us to resolve the geographic, taxonomic, and temporal origins of the Ectemnorhinini and the role of dispersal and colonization in the development of the region’s biogeography. We first resolve the controversial origins of these weevils (19, 30) with a phylogenomic approach using anchored hybrid enrichment (AHE) for up to 515 genes across 12 representative species of Ectemnorhinini and a worldwide sample of 87 species of putative relatives and known outgroups, mostly from the beetle subfamily Entiminae (18, 30, 31). We then build on these outcomes by exploring the timing and patterns of taxonomic diversification, including divergence times and proposed dispersal events, using a multilocus phylogenetic dataset (three mitochondrial and two nuclear genes) for an extensive sample of Ectemnorhinini from each archipelago on which they are known to occur. Finally, we reveal contemporary limits to gene flow and examine the population structure of the littoral-dwelling ectemnorhinine weevil *Palirhoeus eatoni* using phylogeographic methods applied to a library of 5,859 genome-wide single-nucleotide polymorphisms (SNPs). This unusually widespread species is found on all four archipelagos of the Kerguelen Province known to host Ectemnorhinini: Crozet, Kerguelen, Prince Edward Islands (PEI), and Heard Island and McDonald Islands (HIMI).

## Results

### Phylogenomics.

**Fig. 1.**
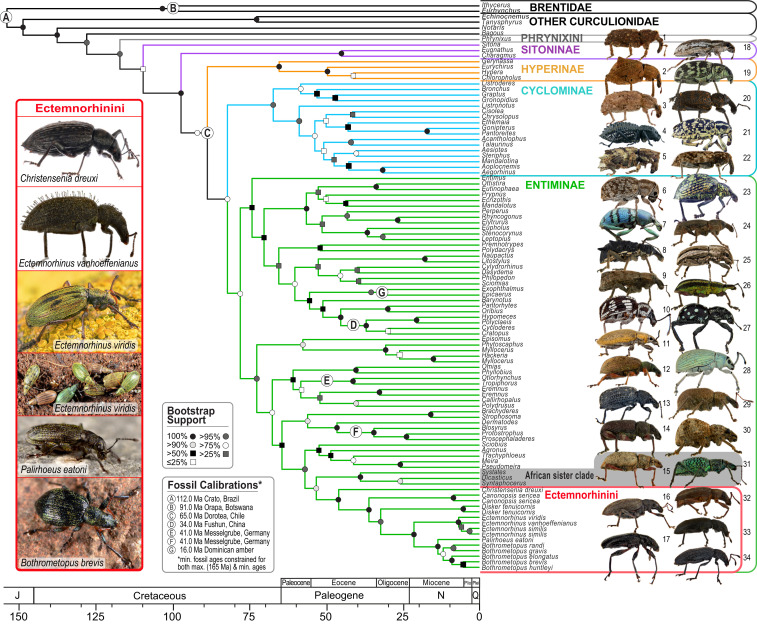
Dated phylogeny of the weevil subfamily Entiminae and relatives, showing the phylogenetic position and nearest relatives of the Ectemnorhinini, inferred from 515 nuclear genes. Major clades (focal subfamilies and tribes) are color coded, and outgroup taxa (Brentidae and other Curculionidae) are shown in black. Letters indicate nodes in the phylogeny constrained by fossil priors (*SI Appendix*, Text S5), and bootstrap support is indicated by node shading (error bars for node age estimates and exact bootstrap values are shown in *SI Appendix*, Fig. S1). (*Right*) Exemplar taxa are shown to illustrate morphological diversity: 1) *Phrynixus terreus*, 2) *Eurychirus bituberculatus*, 3) *Ethemaia sellata*, 4) *Gagatophorus* sp. (not sampled, but a near relative of *Acantholophus*), 5) *Aesiotes notabilis*, 6) *Eutinophaea nana*, 7) *Eupholus* sp., 8) *Leptopius gladiator*, 9) *Cylydrorhinus caudiculatus*, 10) *Oribius gestroi*, 11) *Hypomeces obscurus*, 12) *Phyllobius calcaratus*, 13) *Otiorhynchus sulcatus*, 14) *Sciobius marshalli*, 15) *Dicasticus funicularis*, 16) *Canonopsis sericea*, 17) *Palirhoeus eatoni*, 18) *Sitona discoideus*, 19) *Eugnathus* sp., 20) *Bronchus furvus*, 21) *Chrysolopus spectabilis*, 22) *Steriphus major*, 23) *Entimus imperialis*, 24) *Prypnus fallax*, 25) *Naupactus peregrinus*, 26) *Exophthalmus sulcicrus*, 27) *Pantorhytes stanleyanus*, 28) *Myllocerus aurifex*, 29) *Eremnus segnis*, 30) *Blosyrus* sp., 31) *Systates perblandus*, 32) *Christensenia antarctica*, 33) *Disker tenuicornis*, and 34) *Bothrometopus gracilipes*. (*Left*) Four of the six sampled genera of Ectemnorhinini (the bottom four of these images were taken by Bernard Chaubet; the authors took all others).

We generated a fossil-calibrated Bayesian timetree for 12 Ectemnorhinini species and 87 phylogenetically relevant taxa from all major biogeographic regions worldwide. Our analyses were based on data from the first and second codon positions of 515 genes (111,294 base pairs [bp] total) recovered by AHE. The resulting timetree revealed that Ectemnorhinini weevils diverged from their most recent common ancestor with other weevils ∼55 Ma ago (Fig. 1, 95% highest posterior density [HPD]: 44 to 65 Ma; *SI Appendix*, Fig. S1). The closest relatives of Ectemnorhinini are three African genera, *Systates*, *Dicasticus*, and *Syntaphocerus* (Fig. 1), the latter two representing the African tribe Embrithini and the first representing an African group often confused with the tribe Peritelini. Marginal likelihood estimates obtained via stepping stone analysis were used to test alternative hypotheses for the nearest extant relatives of Ectemnorhinini. These analyses provided additional support for a sister relationship with the aforementioned African genera (*SI Appendix*, Text S1 and Figs. S2 and S3). Tribes previously proposed to be the nearest living relatives of Ectemnorhinini based on morphology, such as the Phyllobiini (30) and the Leptopiini (18), represented in our analysis by *Phyllobius* and *Leptopius*, were not found to be close relatives (Fig. 1 and *SI Appendix*, Figs. S2 and S3). However, in agreement with morphological hypotheses, *Christensenia* from the Crozet archipelago was recovered as the most basal genus of the Ectemnorhinini (18), estimated to have diverged from the rest of the tribe in the Eocene (95% HPD: 37 to 57 Ma; *SI Appendix*, Fig. S1), followed by the monospecific genera *Canonopsis* and *Disker.* The two most diverse clades of Ectemnorhinini, the *Ectemnorhinus* and the *Palirhoeus–Bothrometopus* groups, diverged in the Miocene (Fig. 1).

### Phylogenetics.

A broader taxon sample of 24 Ectemnorhinini species (two-thirds of known species in the group) was sequenced for five genes (2,812 bp total), with phylogenetic analysis resulting in congruent Bayesian and maximum likelihood phylogenies that challenge traditionally recognized diversity in the tribe (*SI Appendix*, Fig. S4). In the molecular phylogenies, distinct clades were evident for island populations of some morphologically determined species, whereas other morphologically determined species failed to show any clear delineation; we therefore proposed molecular operational taxonomic units (MOTUs) based on these phylogenies (*SI Appendix*, *Designation of Molecular Operational Taxonomic Units (MOTUs)*). The phylogenetic trees were generally well supported, although some nodes in the genera *Bothrometopus* and *Ectemnorhinus* had relatively low support, possibly reflecting incomplete lineage sorting due to recent radiation (32) (*SI Appendix*, Fig. S4). Bayesian timetrees calibrated using the geological emergence of the PEI archipelago had largely congruent topologies and date estimates, whether individual gene trees were linked in BEAST (Bayesian Evolutionary Analysis Sampling Trees) or coestimated within a shared species tree in starBEAST and whether or not secondary constraints from the phylogenomic tree were applied (*SI Appendix*, Figs. S5–S7). These trees were also reasonably concordant with the phylogenomic timetree, with error bars for most key divergence dates overlapping. However, date estimates obtained via the phylogenomic analyses were typically somewhat older (*SI Appendix*, *Supplementary phylogenomic results*), and unsampled Ectemnorhinini species could potentially affect these estimates.

**Fig. 2.**
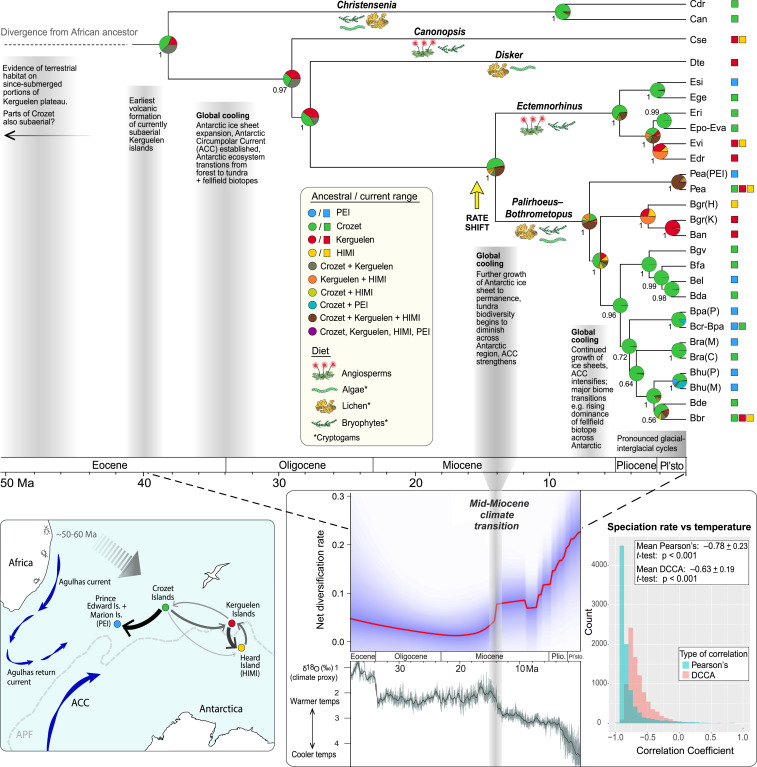
Dated phylogeny of the Ectemnorhinini inferred from three mitochondrial and two nuclear genes, together with key diversification and biogeographic inferences. Bayesian posterior support is provided to the left of each node (error bars can be found in *SI Appendix*, Fig. S5). Pie charts show the probability of ancestral ranges for each node as estimated using the DEC+*J*+*X* biogeographic model of range evolution. The primary diet for each genus is provided below stem branches, and the tips are labeled with MOTU codes (*SI Appendix*, Table S1) with key geo-climatic events shown on the timeline, and the diversification rate shift detected by BAMM (with highest posterior probability; *SI Appendix*, *Diversification analyses*) indicated with an arrow. (*Lower Left*) A schematic map with the hydrography of the Southern Ocean’s Kerguelen Province and surrounds, including the Antarctic Circumpolar Current and Antarctic Polar Front, with arrows (and their widths) among islands indicating the frequency of dispersal events as inferred by historical biogeographic analysis (DEC+*J*+*X* model). (*Lower Right*) The net diversification rate estimated in BAMM (red line with purple shading showing 95% CI) together with a paleoclimate record based on deep-sea benthic oxygen isotopes, from ref. 97, adapted with permission from AAAS (light gray represents climate smoothed over 20 kyr intervals and dark gray over 1 Ma intervals). Coefficients for the correlation between speciation rates estimated in BAMM and paleotemperature (smoothed over 1 Ma) are shown: both Pearson’s and detrended cross-correlation analysis coefficients indicated a significant inverse relationship.

The crown age estimate for Ectemnorhinini based on the most thoroughly calibrated BEAST timetree was 38 Ma (Fig. 2, 95% HPD: 29 to 48 Ma; *SI Appendix*, Fig. S5), which is compatible with maximum age estimates for subaerial land on the Kerguelen Plateau (26), although divergence of the group from its African relatives necessarily occurred earlier than this (Fig. 2). The divergence of the two species-rich clades of Ectemnorhinini, each of which broadly represents a feeding niche (cryptogams versus angiosperms) (33), occurred in the mid-Miocene (14 Ma, 95% HPD: 9 to 19 Ma; *SI Appendix*, Fig. S5), with most of the diversification in these groups (at least of extant and sampled species) occurring throughout the Pliocene and Pleistocene (Fig. 2). Estimation of diversification rates in BAMM (Bayesian Analysis of Macroevolutionary Mixtures) identified a single significant rate shift (increase) occurring in the Miocene just before the crown divergence of these species-rich clades (Fig. 2 and *SI Appendix*, *Diversification analyses*). Furthermore, there was substantial evidence of an inverse relationship between BAMM-generated diversification rates and paleoclimate data (an increase in diversification with decreasing temperature) based on Pearson’s correlations and, more importantly, detrended cross-correlation analysis (Fig. 2). This correlation was significant whether temperature trends were smoothed over either million-year or 20,000-y windows (Fig. 2; cf. *SI Appendix*, Fig. S9). Furthermore, in comparing among constant-rate, time-dependent, and temperature-dependent diversification models, RPANDA (Phylogenetic Analyses of Diversification) allocated the highest support to the model in which speciation rate increases exponentially with decreasing temperature, although other diversification processes may also have played a role (*SI Appendix*, Table S3).

Ancestral range reconstruction using a range of biogeographic models consistently designated the older archipelagos of Crozet or Kerguelen (or both) as the likely range for ectemnorhinine early ancestors, with Crozet remaining the likely ancestral range at most nodes, even after the emergence of Heard Island and the PEI (dispersal–extinction–cladogenesis [DEC]+*J*+*X* model: Fig. 2; simpler models: *SI Appendix*, Figs. S10 and S11). Biogeographic stochastic mapping (BSM) thus unsurprisingly inferred a high degree of speciation in, and dispersal from, the Crozet archipelago (Fig. 2 and *SI Appendix*, *Historical biogeography inference*). The estimate for parameter “*X*” (a distance-based modifier of dispersal) was ∼2 (*SI Appendix*, Table S4), indicating an inverse square function of dispersal probability with increasing geographic distance as commonly predicted by ecological theory (34). Nonetheless, at least 25 interarchipelago dispersal events (mostly reflecting anagenetic range expansion: *SI Appendix*, Fig. S12) were estimated throughout the phylogeny by BSM, the majority of which occurred in an east–west direction against the prevailing winds and currents of the Antarctic Circumpolar Current (Fig. 2; and for a comparison of all models, *SI Appendix*, Tables S5–S7). Regardless of the biogeographic model employed, within-archipelago speciation was inferred as the dominant mode of cladogenesis (*SI Appendix*, Table S4 and Fig. S12), occurring primarily on the Crozet and Kerguelen archipelagos (*SI Appendix*, Fig. S13).

### Phylogeography.

**Fig. 3.**
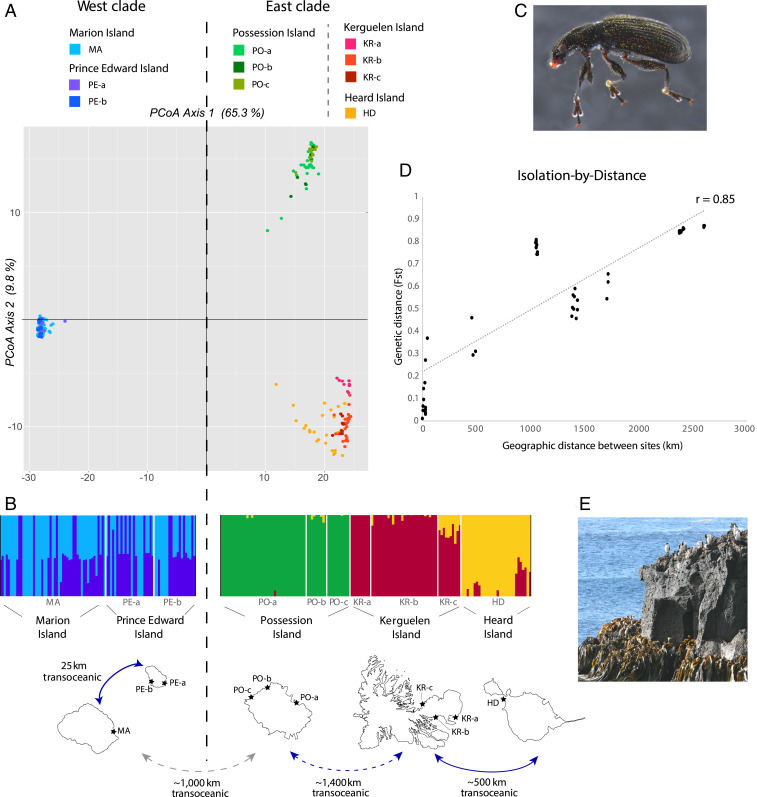
Phylogeographic structure in the marine weevil *Palirhoeus eatoni *(Ectemnorhinini). Principal Coordinate Analysis (*A*) demonstrates the deeply divergent west and east clades. Bayesian cluster analysis (*B*) was conducted separately on these clades to explore finer-level structuring (cluster results based on the entire dataset are provided in *SI Appendix*, Figure S14). Diagrammatic representation of the sites on each island and the population connectivity requiring transoceanic dispersal is shown below the cluster analysis, while an image of *P. eatoni* (from Marion Island) is provided in panel *C*. A significant isolation-by-distance correlation was found (*D*)—for statistics regarding this correlation, see *SI Appendix*, Table S10. A photo of the littoral zone on Marion Island (*E*) demonstrates the dynamic rockface habitat of *P. eatoni *with abundant kelp for rafting.

The population structure in the marine weevil *Palirhoeus eatoni* based on the analysis of 5,859 SNPs showed a deep genetic break between the PEI archipelago (“west” clade) and all other islands (“east” clade), explaining over 65% of the variation in principal coordinate analysis (PCoA) (Fig. 3*A*). Between the two clades, *F*_ST_ values ranged from 0.77 to 0.88, and up to one-third of SNPs were fixed for different alleles (*SI Appendix*, Tables S8 and S9), indicating a complete absence of contemporary gene flow and the likelihood that these populations are undergoing allopatric speciation. The magnitude of this division supports our designation of MOTUs to capture additional diversity in the phylogenetic timetree, in which the same east and west clades were observed for *P. eatoni* (*SI Appendix*, Fig. S4). A further subdivision into two clusters was evident within the east clade—the Crozet archipelago formed one cluster, and the Kerguelen and HIMI archipelagos formed another (Fig. 3 and *SI Appendix*, Fig. S14). Considerable transoceanic dispersal between Prince Edward and Marion islands and between Kerguelen and Heard islands (despite separation by >500 km open ocean in the latter case) was demonstrated by their admixture in Bayesian cluster analysis (Fig. 3*B*). Nonetheless, a strong isolation-by-distance (IBD) correlation (*r* = 0.85; Fig. 3*D*), which remained significant when correcting for cluster in partial Mantel tests (*SI Appendix*, Table S10), indicates that dispersal in *P. eatoni* is distance dependent in agreement with similar findings from the historical biogeographic analysis of our phylogenetic timetree. Compared to archipelago-level structure, intraisland structure was weak (Fig. 3*A*) though detectable on Kerguelen Island (*F*_ST_ = 0.17 to 0.37; *SI Appendix*, Table S8). None of the populations were significantly different in terms of genetic diversity or inbreeding (*SI Appendix*, Table S11).

## Discussion

### Antarctic Life Diversified as the Planet Cooled.

Despite growing recognition that extant terrestrial life survived in the Antarctic throughout glacial cycles (6, 14, 16), little rigorous molecular evidence is available for the tempo or mode of terrestrial diversification, nor for prolonged terrestrial evolution throughout multiple epochs (35). Here, we have integrated ∼120,000 bp of genomic data with multiple calibrations and analyses to demonstrate that Ectemnorhinini beetles arrived in the sub-Antarctic from Africa around 50 Ma ago, repeatedly dispersing along the Antarctic Polar Front and diversifying as temperatures decreased from the mid-Miocene throughout the Plio–Pleistocene and up to the present day. This beetle diversification is synchronous with that of several marine groups that have radiated primarily across the Southern Ocean, including penguins (36), fishes (17), limpets (37), and cryptic-species complexes of octocorals (38) and amphipods (39). Although such a pattern may reflect a variety of extinction and speciation scenarios (40), its prevalence across diverse taxa and over a period of pronounced environmental transitions is unlikely to be coincidental and aligns predictably with evolutionary theory (e.g., refs. 41 and 42).

Significant global cooling, ice sheet growth, and oceanographic changes during the middle and late Miocene climate transitions (∼14 and ∼7 Ma ago) finalized the isolation of Antarctica and the establishment of permanent polar conditions both marine and terrestrial (43). The emergence of most Antarctic marine biodiversity around this time has been attributed to these profound environmental changes, which provided ecological opportunities (e.g., new habitat and diet niches, extinction of predators) for marine life to exploit (2, 17, 36). This also seems to be the case for Ectemnorhinini weevils. On land, the major Miocene ecosystem transition in Antarctica was the loss of angiosperm plant diversity (44), leaving cryptogam flora and fellfield biotopes dominant on the continent (45) and even in the sub-Antarctic, where angiosperm communities waxed and waned in glacial refugia (46). The Ectemnorhinini, which appear to have undergone an acceleration in diversification from the mid-Miocene, represent one of the few beetle groups worldwide to have radiated on a primarily cryptogam (rather than angiosperm) diet (47). Most ectemnorhinines are also particularly well adapted to the epilithic setting provided by fellfield habitat (33). Therefore, a Miocene transition to polar terrestrial conditions likely promoted the diversification of these weevils (especially the cryptogam-feeding *Bothrometopus* clade), which capitalized on novel polar niches, mirroring the region’s marine scenario. Vacant ecological niches may have also been contributed by earlier extinctions, promoting “rebound” speciation in the Miocene (4).

Following a Miocene increase in diversification rate, the most significant accumulation of ectemnorhinine lineages occurred over the last 5 Ma, including angiosperm-feeding lineages, reflecting the persistence of at least some angiosperms throughout the region’s cooling (27, 46). Indeed, diversification appears to be ongoing as indicated by significant phylogeographic structure for the widespread species *P. eatoni*. A similar pattern of continued or accelerated diversification over the Plio–Pleistocene has been documented in many Antarctic marine groups (36, 38, 48). Moreover, preliminary evidence indicates the same timing of diversification processes for other terrestrial groups, such as mites (49), mosses (50), springtails (16), and diatoms (12), with diversification ongoing in some of these groups too (51). Ongoing speciation across such a fragmented terrestrial habitat should not be surprising, yet Antarctica has only recently been acknowledged as a dynamic region actively generating diversity and, even then, mainly for marine life (4, 5). As deeper molecular attention is paid to lesser-known terrestrial taxa from the Antarctic continent (e.g., bacteria, tardigrades), additional endemic diversity is invariably uncovered, implying extended histories in the region (15, 52). Given the island-like habitat available to terrestrial biota on the continent reduced to fellfield during the Miocene and further reshaped by >5 Ma of glacial cycles, these lineages have likely undergone allopatric diversification in a manner similar to sub-Antarctic groups. Indeed, this has already been anticipated by phylogeographic studies (53, 54).

Macroevolutionary theory predicts that synchronous diversification across varied taxa should reflect allopatric processes driven by acute changes in the physical environment (41, 42). Indeed, mounting evidence indicates that cooling and habitat restructuring owing to Plio–Pleistocene glacial cycles promoted the remarkable diversification of marine fauna through population fragmentation and recolonization processes (1, 2). Similar processes are likely for the Ectemnorhinini, with increased diversification over the Plio–Pleistocene largely attributed to speciation within (rather than between) the Crozet and Kerguelen archipelagos—both of which experienced substantial glaciation (26, 55). Population divergence across known historical glacial barriers (55) was evident for the weevil *P. eatoni* on Kerguelen Island, and has been demonstrated for numerous other terrestrial species throughout the region (see ref. 10). Founder events arising from long-distance dispersal have additionally played a role as exemplified by the pronounced east–west divergence of *P. eatoni*. The mode and timing of diversification for sub-Antarctic Ectemnorhinini, alongside emerging circumstantial evidence for similar diversification tempos in other Antarctic terrestrial groups, provide strong initial support that the diversification processes common to Antarctic marine taxa are more general. Geo-climatically driven diversification since the mid-Miocene (1, 2) should thus be considered a potential general biodiversity paradigm that warrants explicit testing among additional marine and terrestrial Antarctic taxa.

### Sub-Antarctic Biogeography Characterized by Repeated Colonization.

Unveiling one of the longest Antarctic evolutionary histories to date for an extant terrestrial group not only provides insights about general diversification processes in the region but also informs long-standing biogeographic debates about faunal origins and colonization dynamics in the sub-Antarctic (56–58). African affinities have previously been suggested for invertebrates of the Kerguelen Province islands based on biogeographic and genetic evidence (30, 49, 59). Yet, these origins have been widely debated and clearly do not apply to several taxa (46, 60). Similarly, both the Kerguelen and Crozet archipelagos have been the source of much speculation about their geological origins and roles in the diversification of the Southern Hemisphere fauna (30, 59, 61).

Our analyses strongly support an African origin hypothesis for the Ectemnorhinini, settling this century-long debate. An estimated divergence from African relatives 44 to 65 Ma ago suggests that the ancestors of present-day weevils inhabited the Southern Ocean before the oldest currently subaerial island in the Kerguelen Province. Although historical biogeographic analysis could not distinguish between an ancestral distribution on the Kerguelen or Crozet islands, the former has a maximum age estimate of 39 Ma (26), and the latter has an even younger maximum estimate of 9 Ma (62). However, terrestrial sediments and fossils drilled from the Kerguelen Plateau indicate that submerged components were previously subaerial as early as the Cretaceous (63), with similar proposals made for the age of the Crozet Plateau (59). These ancient lost islands have been implicated as stepping stones for dispersal between Africa and other continents during the early Eocene (64, 65), and our findings certainly imply a potential role for them in the early evolution of the region’s insect fauna. While our dating estimates were based on an incomplete sampling of the Ectemnorhinini, unsampled extant species almost entirely belong to the recently diverged species-rich clades. Taxon sampling is therefore unlikely to significantly affect age estimates for deeper nodes such as the divergence between Ectemnorhinini and African sister taxa.

Our data suggest that long-distance transoceanic dispersal has been integral to both the initial colonization of the sub-Antarctic and the ongoing evolution of Ectemnorhinini weevils. More than 20 interarchipelago dispersal events (not necessarily involving speciation) were inferred throughout the region, with additional dispersal likely having occurred among islands within archipelagos (i.e., between subaerial and since-submerged islands) and involving unsampled lineages. Transoceanic dispersal in flightless weevils is thought to be feasible via rafting on oceanographic currents (66). Initial dispersal of weevils from Africa across the Southern Ocean could certainly have been aided by the Agulhas Return Current and Antarctic Circumpolar Current (Fig. 2) as proposed for other terrestrial taxa (67). Rafting is also a likely dispersal mechanism for *P. eatoni* given its littoral habit and genetic signals of IBD since shorter rafting journeys are likely to occur most frequently (68). Bird-mediated dispersal, however, is strongly indicated for at least some ectemnorhinine species based on our inferences of significant dispersal against the region’s prevailing westward currents. Antarctic seabirds can cover several hundred kilometers in a single day when migrating among sub-Antarctic islands (69), and soil invertebrates are known to attach to their feathers (70). Indeed, physiological data (*SI Appendix*, *Physiological data*) demonstrate that most Ectemnorhinini weevils could survive more than a week of bird-mediated dispersal before succumbing to dehydration or starvation. Irrespective of the mechanisms, our results support the growing paradigm that colonization processes are important in the biogeography of the far Southern Hemisphere (10, 58, 71).

## Materials and Methods

### Samples.

We refer to morphologically described species using the nomenclature established by Kuschel and Chown (18), acknowledging that the taxonomy of the Ectemnorhinini remains contested (20). Ectemnorhinini were collected from Possession Island (Crozet Islands archipelago); Grande Terre Island, or commonly “Kerguelen Island” (Kerguelen Islands archipelago); Prince Edward Island (PEI archipelago); Marion Island (PEI archipelago) and Heard Island (HIMI archipelago), all situated between 46° and 53° south in the Southern Ocean (Fig. 2 and *SI Appendix*, Tables S12–S14). Due to the remote inaccessible nature of these islands and the rarity of many of the species, specimens were collected opportunistically from 2000 to 2018. Ultimately, 24 of the 36 putative species in the group were sampled (*SI Appendix*, Table S13). Weevils were preserved in ethanol until genomic DNA was extracted from leg or thoracic muscle tissue. DNA was extracted using the OmniPrep DNA extraction kit and treated with ribonuclease A before genomic DNA library preparation for samples used in phylogenomic analyses. These specimens are stored in the McKenna Lab collection at the University of Memphis. DNA was extracted from specimens for phylogenetics and phylogeography using the QIAamp DNA Micro Kit. The deposition details for these specimens are provided in *SI Appendix*, Table S13.

### Phylogenomics.

We employed AHE to determine the closest extant relatives of the Ectemnorhinini and estimate their root age and geographic origin in a global framework. We sequenced a subset of 12 Ectemnorhinini species (15 specimens) together with a wide range of taxa from the beetle family Curculionidae (*SI Appendix*, Table S12). The selection of ingroup taxa was focused on the CEGH clade of Curculionidae ("broad-nosed" weevils) (72) including proposed near relatives of Ectemnorhinini, such as Phyllobiini (30), Leptopiini (18), and Pachyrhynchini (31). Because Ectemnorhinini have the southern-most distribution of all weevils in the subfamily Entiminae and are restricted to the South Indian Ocean Province, we more densely sampled Southern Hemisphere entimine genera from the adjacent continents of Africa and Australia, but we also included taxa from South America and the Palaearctic region, such as Naupactini, Phyllobiini, Polydrusini, and Otiorhynchini. For outgroup taxa, we selected some lineages outside of the CEGH clade (Bagoinae, Erirhininae, and Brachycerinae) (72, 73). Representatives of two brentid subfamilies (Ithycerinae and Eurhynchinae) were used to root the phylogeny.

### Library Preparation and AHE.

We followed the user manual for the NEBNext Ultra II DNA Library Prep Kit for Illumina (New England Biolabs). Our methods for AHE were based in part on the protocol described in ref. 74, using probes published in ref. 75. We used myBaits Hyb Capture kits for AHE, following instructions in the user manual (Arbor Biosciences). All AHE data were sequenced using Illumina HiSeq X Ten sequencers (Illumina) and paired-end 150 reads. Before assembly, the raw read fastq files were trimmed using Trimmomatic (76). Raw reads were assembled using SOAPdenovo2 (77) following the genome and sequence read archive assembly methods in ref. 47.

We used a beetle-specific orthologous reference gene set (522 genes) for AHE. Official gene sets were downloaded from OrthoDB v.7 (78). We used Orthograph (79) to generate a profile hidden Markov model from the amino acid sequences of each reference gene from OrthoDB following the list of gene identifications (IDs) in OrthoDB. The Orthograph pipeline was used to search all AHE data in our taxon sample with default settings for all parameters.

Multiple fasta files were generated using the orthology prediction pipeline described above; we handled the sequences with a script provided in the Orthograph package to concatenate the OrthoDB IDs from all species (79). The resulting fasta files, which contained all taxa for each gene, were saved in a folder with files organized by OrthoDB IDs. Multiple sequence alignments (MSA) were then undertaken for each gene using the L-INS-i option (an iterative refinement method) in MAFFT (Multiple Alignment using Fast Fourier Transform) v.7.130b (80). The alignments were checked with Aliscore v.1.2 (81, 82) and for outliers as described in ref. 83. To reconstruct a phylogeny with nucleotide data corresponding to the protein alignments, we employed Pal2Nal v.14 (84), again as outlined in ref. 83. After processing MSAs for outliers and with Aliscore, the filtered fasta files were concatenated to form a supermatrix of 515 genes using FASconCAT v.1.0 (85). The final dataset analyzed contained nucleotides from first and second codon positions (“C12”) and is deposited on the Zenodo Digital Repository (doi:10.5281/zenodo.3955188). All code used in preparing the dataset can be found in ref. 86.

### Phylogenomic Inference and Molecular Dating.

Detailed analytical methodology is provided in *SI Appendix*, Text S4. Briefly, we used PartitionFinder v.1.1.1 (87) to find the best-fit cluster of partitions for the C12 dataset. The dataset was partitioned using PartitionFinder and analyzed under the GTR+G+I model using maximum likelihood (ML) phylogenetic inference conducted in RAxML (Randomized Axelerated Maximum Likelihood) v.8 (88) on the University of Memphis high-performance computing (HPC) cluster. The Phylogenetic Analysis by Maximum Likelihood (PAML) package (89) was employed to generate a timetree and estimate divergence times using a Markov chain Monte Carlo (MCMC) approach. We used the data from first and second codon positions and the partitioned (best) ML tree as input for our analysis. A total of seven fossil calibrations (*SI Appendix*, Text S5) were used as soft minimum ages (truncated Cauchy distributions), and an independent rates model was used to relax the clock. Four separate MCMCtree runs were implemented on the University of Memphis HPC cluster, and the resulting output files were checked for convergence using a custom plotting script in R (90). To further test statistical support for the nearest relatives of the Ectemnorhinini, we conducted marginal likelihood estimation (via stepping stone sampling in MrBayes v.3.2.6). Specifically, we assessed the relative fit of several alternative sister relationship hypotheses using Bayes factors for model comparison (detailed in *SI Appendix*, *Supplementary phylogenomic results*).

### Phylogenetics.

We explored interspecific relationships, divergence times, and dispersal events within Ectemnorhinini by sequencing three mitochondrial and two nuclear gene regions for all 24 morphologically determined species sampled from the tribe. All but one of these species were sequenced from all of the islands within their distribution, and for most species, multiple individuals were sequenced from each island on which they are known to occur (*SI Appendix*, Table S13).

### Multilocus Sanger Sequencing.

Fragments of five genes—cytochrome *c* oxidase I (COI), cytochrome *b*, elongation factor 1-alpha, 16S ribosomal RNA, and 28S ribosomal RNA—were amplified using the QIAGEN Taq DNA Polymerase Kit (details in *SI Appendix*, Table S15). The PCR product was purified by ExoSAP and sequenced in both directions on an Applied Biosystems 3730xl sequencer at Macrogen, Inc. Sequence chromatograms were aligned using the ClustalW algorithm (91) to create consensus sequences for each individual. These were visually checked for indels and translated to amino acids to check for stop codons. Due to the presence of indels, the ribosomal genes 16S and 28S were aligned using the G-INS-i algorithm (an iterative refinement method) in MAFFT, with ambiguously aligned regions deleted using Gblocks v.0.91 and the options set for a less stringent selection. We “RY-coded” purines and pyrimidines for the third codon of COI (see ref. 92) to ameliorate substitution saturation (*SI Appendix*, Table S16 and Fig. S15). The raw sequences for each gene are deposited in GenBank (*SI Appendix*, Table S13).

### Phylogenetic Inference and Molecular Dating.

Detailed methodology regarding phylogenetic analyses and molecular dating, including a schematic overview of all analyses and how they provided cross-checks, is provided in *SI Appendix* Text S6 and Fig. S16). In brief, we initially explored relationships among Ectemnorhinini species using Bayesian inference (BI) and ML analyses of a concatenated dataset of the five genes. BI analysis was carried out in MrBayes on the CIPRES (Cyberinfrastructure for Phylogenetic Research) Science Gateway 3.3 (93) using best-fitting substitution models applied independently to partitions of the sequence data as determined by PartitionFinder (*SI Appendix*, Table S17). ML analysis was conducted with RAxML on the CIPRES Gateway using the GTR+G substitution model applied independently to data partitions as determined by PartitionFinder (*SI Appendix*, Table S17). BI and ML phylogeny estimates were compared to assess congruence—with one another, with the phylogenomic tree generated by AHE, and with morphological species designations. As some phylogenetic clades were inconsistent with morphological species designations (which themselves have been highly contested, see refs. 18 and 20), ML and BI trees were used to designate MOTUs for subsequent analyses as detailed in *SI Appendix*, *Designation of Molecular Operational Taxonomic Units (MOTUs)*. We additionally performed all analyses using morphological species to ensure our use of MOTUs did not significantly affect our interpretations, and the results were highly similar (*SI Appendix*, *Supplementary results based on morphological species rather than MOTUs*). Thus, we only refer to MOTU-derived results in the main text.

Divergence dates among Ectemnorhinini were estimated from our five-gene dataset by linking gene trees, as implemented in BEAST v.2.5 (94), and by coestimating gene trees embedded in a shared species tree, as implemented in starBEAST (95). We randomly selected one sequence to represent each MOTU (or morphological species) for analysis in BEAST. For starBEAST, which performs optimally with multiple samples per lineage, we retained the entire sequence dataset. Uncorrelated relaxed lognormal clocks allowed for rate variation among lineages and a birth–death process was used for the tree prior. For calibration, geological (island formation) constraints were applied as uniform upper bounds, and secondary fossil-derived constraints were applied as normal priors (detailed in *SI Appendix*, Text S7 and Fig. S17). A second analysis was run (in BEAST only) with secondary constraints excluded but with substitution rates calculated previously for Coleoptera provided as lognormal priors for the mean rates of relaxed clocks (*SI Appendix*, Text S7).

### Diversification and Historical Biogeography Inference.

Diversification rates and potential rate shifts for the Ectemnorhinini (based on the most thoroughly calibrated phylogeny generated by BEAST; see *SI Appendix*, Fig. S16) were assessed using an MCMC approach implemented in BAMM v2.5.0 (96). Sampling bias was accounted for using an input file providing the taxonomic coverage for each genus (as per *SI Appendix*, Table S13). We ran four chains of 20 million generations each and analyzed the output with the BAMMtools package for R, checking log likelihood traces and effective sample size values for convergence. For each of the 9,000 simulations of diversification rate retained by BAMM (after a burn-in of 10%), we also tested for a correlation between the estimated speciation rate and paleoclimate (specifically temperature). Deep-sea benthic oxygen isotope data smoothed in both 20,000-y and million-year windows (from ref. 97) were converted to temperature as per ref. 98 to reconstruct paleoclimate records reflecting shorter- and longer-term trends. The correlations were analyzed in R (see ref. 99 for script) using Pearson’s correlation coefficients and, to account for nonstationarity and autocorrelation of the two timeseries, detrended cross-correlation analysis (100). A Student’s *t* test was used to determine if the mean correlation coefficient in each case was significantly different from zero. To further examine the relationship between paleotemperature and speciation rate, we used the RPANDA package for R (101) to assess the fit of various temperature-dependent, time-dependent, and constant-rate diversification models to our phylogenetic tree (using the million-year smoothed paleoclimate data). We used Akaike information criterion correction values and weights to identify the diversification model with the best fit. We restricted comparisons to exponentially variable models because of ongoing difficulties with the way RPANDA handles linear dependencies. Input files and the R script for the RPANDA analyses have been deposited on Figshare (doi:10.26180/14446023).

Biogeographic history was inferred from our BEAST timetree using ML ancestral range estimation carried out with BioGeoBEARS (BioGeography with Bayesian (and likelihood) Evolutionary Analysis in R Scripts) (102). We applied the DEC biogeographic model (103), which includes more biologically relevant assumptions of speciation such as subset sympatry, than, for instance, the Dispersal–Vicariance Analysis model, which assumes vicariance as a default explanation (104). We also ran the model with or without two additional free parameters: founder-event speciation (“+*J*”) and a distance modifier for dispersal probability (“+*X*”), both of which are prudent in the context of widely dispersed islands (105). Statistically comparing the likelihood of each model fit has been deemed inappropriate (106), and thus, we focused on the most biologically relevant model which includes all parameters (DEC+*J*+*X*) and compared outcomes from the simpler models. In each instance, we carried out 100 iterations of BSM (107) to infer the average number and type of cladogenetic and anagenetic dispersal and speciation events throughout the evolutionary history of the phylogeny. Analyses were time stratified such that Heard Island and the PEI could not be ancestral ranges before their subaerial appearance 22 (108) and 0.475 Ma ago (109), respectively. The ranges were designated at the archipelago level (i.e., the geographically proximal Marion and Prince Edward islands were considered together as the PEI) so that dispersal inferences were uniformly interpreted as long-distance among-archipelago events. Geographic ranges and the distances between each archipelago (used as input data) are provided in *SI Appendix*, *Input data for historical biogeographic inference* and on Figshare, along with all associated R scripts (doi:10.26180/14446023).

### Phylogeography.

We explored contemporary gene flow among islands using genome-wide SNPs for *Palirhoeus eatoni,* the most widespread and only supralittoral-dwelling ectemnorhinine species. We sampled *P. eatoni* from one site each on Marion and Heard islands and from multiple sites on Prince Edward, Possession, and Kerguelen islands, enabling exploration of intraisland as well as interisland population genetic structure (*SI Appendix*, Table S14). Marion Island was sampled at two different time points, though preliminary analyses failed to demonstrate any significant temporal difference (regarding allele frequencies or overall diversity); therefore, these samples were pooled to create a single population for Marion.

### SNP Library Preparation, Genotyping, and Quality Filtering.

A genome-wide SNP library for *P. eatoni* was developed using Diversity Arrays Technology sequencing (DArT-Seq) (110) at DArT Pty. Ltd. DArT-Seq is a reduced-representation next-generation sequencing method, similar to double-digest restriction-associated DNA sequencing. A customized combination of restriction enzymes was used to digest genomic DNA, adaptors were ligated to restriction enzyme overhangs, fragments were PCR amplified and sequenced on an Illumina HiSeq 2500, and raw Illumina data files were processed with a proprietary DArT pipeline to provide a quality-filtered SNP dataset. The SNP dataset generated by DArT was further filtered using the “dartR” package v.1.0.5 (111) in R. A detailed explanation of DArT-Seq SNP library preparation, quality control, and filtering measures is provided in *SI Appendix*, *Supplementary methods for phylogeography*, and the final filtered SNP dataset is available on Figshare, together with code for all analyses performed in R (doi:10.26180/14446023).

### Phylogeographic Analyses.

The final dataset was tested for evidence of selection using the *F*_ST_ outlier approach and a false discovery rate of α = 0.01 in BayeScan 2.1 (112). SNPs were also tested for deviations from the Hardy–Weinberg Equilibrium (HWE) in each population, using exact tests conducted in dartR and adjusted with the Bonferroni correction. SNPs putatively under selection or found to violate HWE in any single population each represented less than 5% of the total dataset, and only 15 SNPs (0.3% of the dataset) violated HWE in more than one population. Therefore, while a conservative dataset was prepared with all of these SNPs removed, it was only used for a subset of analyses to check that the removal of these loci did not affect results. Indeed, results were congruent with those for the full dataset (*SI Appendix*, *Phylogeographic results for conservative SNP dataset*); thus, we only refer to the full dataset in the main text.

Genetic diversity and population structure were assessed within and among sites as detailed in *SI Appendix*, Text S9. In summary, we calculated observed and expected heterozygosity, inbreeding coefficients, and pairwise *F*_ST_ estimates among all populations using GenoDive v.2.0 (113). PCoA was conducted in dartR to observe the clustering of individuals due to genetic similarity, and a Bayesian clustering algorithm was also used to infer population structure as implemented in fastSTRUCTURE (114). It was clear that there were two pronounced genetic clusters of the data (“east” and “west”), which swamped all finer-level genetic structure; therefore we additionally conducted a fastSTRUCTURE analysis within each of these clades independently. As the identification of population clusters can be confounded by an underlying pattern of IBD and vice versa (115), we explored our dataset for a linear relationship between genetic distance (*F*_ST_) and geographic distance between sites (input data: *SI Appendix*, Table S25), which would indicate IBD. We carried out partial Mantel tests using the “ncf” package v.1.2–8 (116) in R, testing whether IBD patterns remained significant while correcting for clusters as a covariate and whether clusters remained significant after correcting for geographic distance. Finally, we explored the dataset for fixed allelic differences between populations (when no alleles are shared for a given SNP), which indicates a complete absence of contemporary gene flow (117), using dartR.

## Supplementary Material

Supplementary File

## Data Availability

Data on genetic material contained in this paper are published for noncommercial use only. Utilization by third parties for purposes other than noncommercial scientific research may infringe the conditions under which the genetic resources were originally accessed and should not be undertaken without obtaining consent from the corresponding author of the paper and/or obtaining permission from the original provider of the genetic material. Phylogenomic (AHE) data have been deposited in Zenodo (DOI:10.5281/zenodo.3955188) (118); phylogeographic (SNP) data have been deposited in Figshare (DOI:10.26180/14446023) (119) and all phylogenetic sequences have been deposited in GenBank (accessions MT701045 and MT701503).
