# Supplementary material for: Fifty million years of beetle evolution along the Antarctic Polar Front
Source: Proc Natl Acad Sci U S A. 2021 Jun 9;118(24):e2017384118. doi: 10.1073/pnas.2017384118 (PMC8214695; doi:10.1073/pnas.2017384118)
Supplement: Supplementary File [file pnas.2017384118.sapp.pdf]

PNAS
www.pnas.org

Supplementary Information for:

Fifty million years of beetle evolution along the Antarctic Polar Front

Helena P. Baird*, Seunggwan Shin, Rolf G. Oberprieler, Maurice Hullé, Philippe Vernon, Katherine L. Moon, Richard H. Adams, Duane D. McKenna and Steven L. Chown

* Corresponding author: Helena P Baird
School of Biological Sciences, Monash University, VIC 3800 Australia
helena.baird@monash.edu

The contents of this PDF file are as follows:

i. Supplementary phylogenomic results

Figure S1: Dated phylogeny for Ectemnorhinini and all 87 weevil relatives and outgroups, based on phylogenomic dataset

Text S1: Evaluation of alternative hypotheses for the nearest relatives of Ectemnorhinini

Figure S2: Phylogeny showing the alternative topological hypotheses tested using marginal likelihood estimation

Figure S3: Distributions of marginal likelihood estimates for alternative topological hypotheses

ii. Designation of Molecular Operational Taxonomic Units (MOTUs)

Text S2: MOTU designation criteria

Table S1: List of MOTUs designated based on phylogenetics

Figure S4: Maximum Likelihood and Bayesian phylogenies showing MOTU designation

iii. Supplementary phylogenetic results

Figure S5: Dated phylogeny for Ectemnorhinini based on phylogenetic dataset, generated in BEAST using geological and secondary constraints

Figure S6: Dated phylogeny for Ectemnorhinini based on phylogenetic dataset, generated in BEAST using geological constraints and substitution rates

Figure S7: Dated phylogeny for Ectemnorhinini based on phylogenetic dataset, generated in starBEAST using geological and secondary constraints

iv. Diversification analyses

Table S2: Bayes factor matrix comparing different diversification models to a null model of no rate heterogeneity

Figure S8: 95% credible set of distinct diversification rate shift configurations

Figure S9: Correlation coefficients for speciation rate vs paleotemperature

Table S3: Outcomes of diversification model selection using RPANDA

v. Historical biogeography inference

Table S4: Outcomes of historical biogeographic inference using BioGeoBEARS

Figure S10: Ancestral range estimation using the DEC biogeographic model

Figure S11: Ancestral range estimation using the DEC+*J* biogeographic model

Table S5: Mean number of inferred inter-archipelago dispersal events using the DEC biogeographic model

Table S6: Mean number of inferred inter-archipelago dispersal events using the DEC+*J* biogeographic model

Table S7: Mean number of inferred inter-archipelago dispersal events using the DEC+*J*+*X* biogeographic model

Figure S12: Frequency distributions of inferred cladogenetic and anagenetic events (for all three models)

Figure S13: Frequency distribution of inferred within-archipelago speciation events using the DEC+*J*+*X* biogeographic model

vi. Supplementary phylogeographic results

Table S8: Matrix of pairwise F_{ST} values among *Palirhoeus eatoni* populations

Table S9: Matrix of pairwise fixed allelic differences among *P. eatoni* populations

Figure S14: fastStructure plot showing population clustering in *P. eatoni*

Table S10: Outcomes for partial Mantel tests of isolation-by-distance in *P. eatoni*

Table S11: Genetic diversity statistics

vii. Physiological data

Text S3: Physiological evidence supporting zoochory of ectemnorhine weevils by seabirds

viii. Specimen details and sequence data deposition

Table S12: List of all weevil species used for phylogenomic analysis, with corresponding codes relating to Anchored Hybrid Enrichment data deposited on Zenodo

Table S13: List of all Ectemnorhinini species, collection details for specimens used for phylogenetics, and corresponding accession codes for sequences deposited on Genbank

Table S14: Collection details for specimens of *P. eatoni* used for phylogeography

ix. Supplementary methods for phylogenomics

Text S4: Phylogenomic inference and molecular dating: detailed methods

Text S5: Fossil choice and placement for phylogenomic timetree calibration

x. Supplementary methods for phylogenetics

Table S15: PCR primers and thermal protocol

Table S16: Results of Xia's test for substitution saturation

Figure S15: Saturation plot for COI third codon

Text S6: Phylogenetic inference and molecular dating: detailed methods

Figure S16: Overview of phylogenetic and biogeographic analyses and cross-checks carried out on sequence data

Table S17: Best-fit partitioning strategies and substitution models

Text S7: Prior settings and time calibrations used in BEAST and starBEAST analyses

Figure S17: Placement of calibrations and constraints on phylogeny of Ectemnorhinini

xi. Supplementary results based on morphological species rather than MOTUs

Figure S18: Dated phylogeny for Ectemnorhinini based on phylogenetic dataset with morphological species at tips, generated in BEAST using geological and secondary constraints

Table S18: Outcomes of historical biogeographic inference using BioGeoBEARS

Figure S19: Ancestral range estimation using the DEC+*J*+*X* biogeographic model

Table S19: Mean number of inferred inter-archipelago dispersal events

xii. Input data for historical biogeographic inference

Table S20: Distribution ranges for each Ectemnorhinini species and MOTU

Table S21: Matrix of pairwise distances among ranges (archipelagos)

xiii. Supplementary methods for phylogeography

Text S8: Genome-wide SNP library preparation and quality filtering

Table S22: Size of SNP dataset at each stage of quality filtering

Text S9: Phylogeographic analyses of SNP dataset: detailed methods

xiv. Phylogeographic results for conservative SNP dataset

Table S23: Genetic diversity statistics

Table S24: Matrix of pairwise F_{ST} values among *P. eatoni* populations

Figure S20: PCoA for *P. eatoni* populations

xv. Input data for isolation-by-distance analysis

Table S25: Matrix of pairwise distances among sites sampled for *P. eatoni*

xvi. References for all Supplementary Information

i. Supplementary phylogenomic results

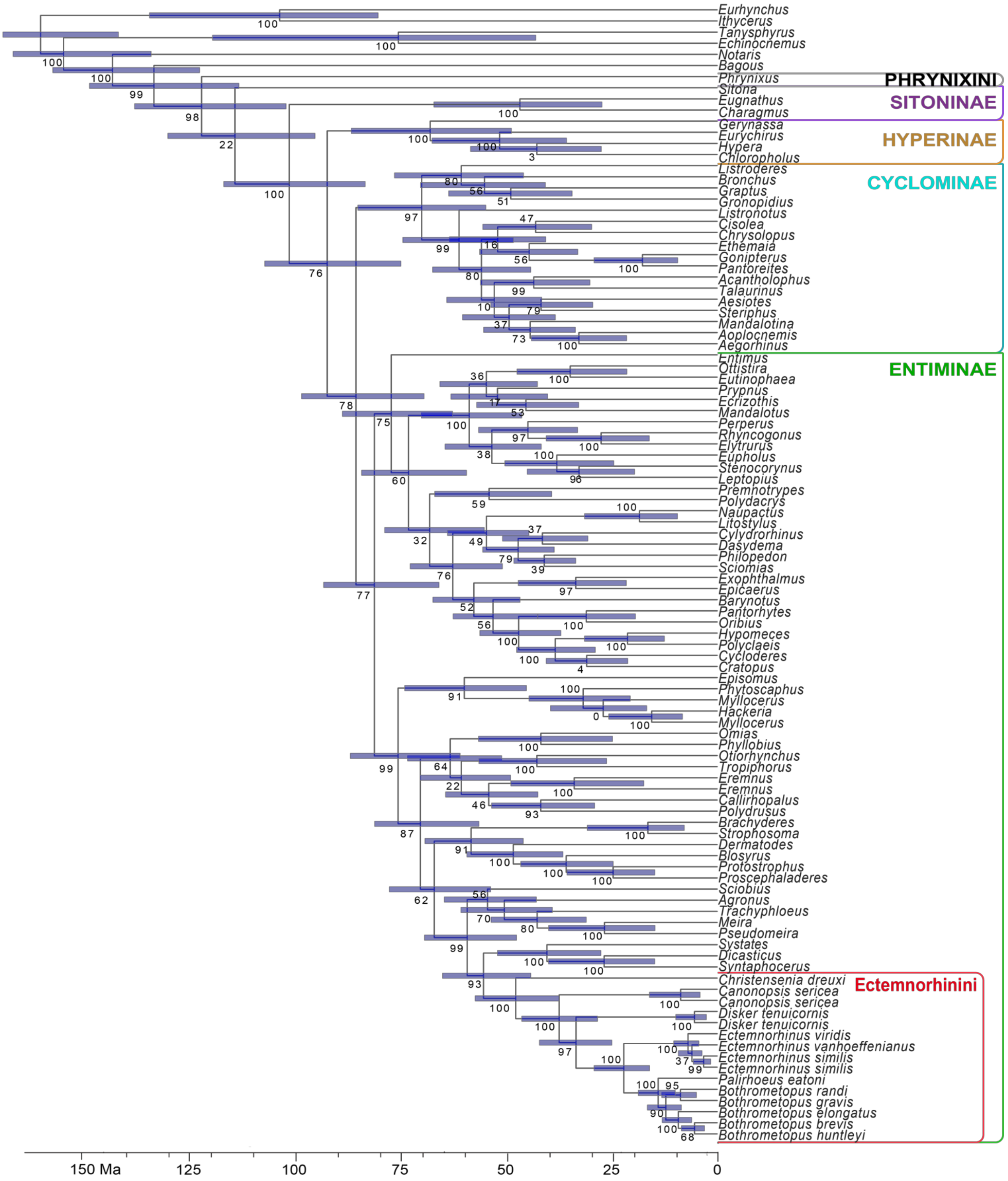

Figure S1 Dated phylogeny of weevil subfamily Entiminae and near relatives, showing the phylogenetic position of Ectemnorhinini, generated in MCMCTree using a dataset of 1st and 2nd codons for 515 nuclear protein-coding genes and calibrated with fossils (see Text S5). Error bars represent 95% highest posterior densities for estimated node age. Bootstrap support is shown to the left of nodes: 38% of all nodes had maximal (100%) ML bootstrap support (MLBS); 18% had >90% MLBS; 11% had 76-90% MLBS; 15% had 51-75% MLBS, and 18% had MLBS ≤50%. The raw tree file in Nexus format can be found on Figshare (doi:10.26180/14446023).

Text S1: Evaluation of alternative hypotheses for the nearest extant relatives of the Ectemnorhinini

We conducted an array of phylogenetic model selection analyses to investigate statistical support for competing hypotheses based on morphology concerning the nearest extant relatives of Ectemnorhinini weevils. A potential sister group relationship between Ectemnorhinini and each of three other major lineages of Entiminae was evaluated:
1. Leptopiini (represented by *Ottistira*, *Eutinophaea*, *Prypnus*, *Ecrizothis*, *Mandalotus*, *Perperus*, *Rhyncogonus*, *Elytrurus*, *Eupholus*, *Stenocorynus*, *Leptopius*);
2. Phyllobiini (represented by *Omias* and *Phyllobius*);
3. A clade consisting of three African genera (*Systates*, *Dicasticus* and *Syntaphocerus*) that was recovered in our original molecular analysis (see Figure 1, Figure S1).
We refer to these three hypotheses as "morphology A" (Ectemnorhinini + Leptopiini), "morphology B" (Ectemnorhinini + Phyllobiini), and "molecular" (Ectemnorhinini + the African clade), respectively. The first two hypotheses ("morphology A" and "morphology B") represent alternative sister relationships proposed in previous studies based on morphological characters (1, 2), while the "molecular" hypothesis represents the inferred topology from our concatenated set of 515 nuclear genes (see Figure 1, Figure S1). The weevil taxa used to define the monophyly constraints for each analysis are shown in Figure S2.

To evaluate statistical support for the three competing hypotheses, we conducted marginal likelihood estimation in order to assess relative fit using Bayes factor ('BF'; ref. 3) model comparisons. Marginal likelihoods were estimated independently for each hypothesis using stepping-stone sampling (4-6) as implemented in MrBayes v.3.2.6. Our alignment of 515 nuclear genes and associated partitioned models inferred from PartitionFinder were used as input for these analyses. For each hypothesis, we specified monophyly constraints such that interspecific relationships both inside and outside the proposed clade were free to vary throughout the MCMC sampling process within the constraints of the sister grouping of Ectemnorhinini with either Leptopiini (i.e. "morphology A"), Phyllobiini (i.e. "morphology B"), or the African lineages (i.e. "molecular"), respectively (see Figure S2). Following recommendations of previous studies (e.g. ref. 5), we conducted the stepping-stone analyses for a total of 50 steps across 20 million MCMC iterations, with sampling every 10,000 iterations after discarding the first 39,000 iterations as burn-in. Additionally, the first 90,000 samples were discarded from the beginning of each step. We employed Metropolis coupling with 16 chains for every stepping-stone analysis, and we conducted 10 separate MrBayes runs in parallel to obtain a total of 10 replicates of marginal likelihood estimates for each of the three hypotheses. We evaluated the fit of the two morphology hypotheses (i.e., "morphology A" and "morphology B") relative to the "molecular" hypothesis by computing Bayes Factors based on the average marginal likelihood measured across the 10 replicates for each hypothesis. That is, we compared models by computing the following Bayes Factors:

$$\mathrm{BF_A} = 2(\mathrm{MLE_{molec.}} - \mathrm{MLE_{morph.\ A}})$$
$$\mathrm{BF_B} = 2(\mathrm{MLE_{molec.}} - \mathrm{MLE_{morph.\ B}})$$

where $\mathrm{MLE_{morph.\ A}}$, $\mathrm{MLE_{morph.\ B}}$, $\mathrm{MLE_{molec.}}$ denote the average log-transformed marginal likelihood estimate obtained for "morphology A", "morphology B", and "molecular", respectively. Evidence in favor of the "molecular" hypothesis is found with increasingly larger Bayes Factors, with values greater than 6 indicating strong support and values less than 2 indicating negligible support.

Marginal likelihood estimates obtained from stepping-stone analysis with MrBayes revealed strong evidence in support of the "molecular" hypothesis that was recovered in our dated phylogenetic analyses of 515 nuclear genes ($MLE_{molec.}$= -627666.3 for Ectemnorhinini + the African clade; Figure S3). Evaluation of monophyly model fit using Bayes Factors suggested that both of the alternative hypotheses based on phenotypic characters (i.e., "morphology A" and "morphology B") exhibited relatively poor overall fit compared to the "molecular" hypothesis, as demonstrated by BF_A= 2459.3 and BF_B= 740.9, respectively (Figure S3). These conclusions were found to be consistent across replicate runs, such that marginal likelihood estimates based on the "molecular" hypothesis were always greater than those of the other two alternative hypotheses (i.e., distributions of marginal likelihoods shown in Figure S3). Combined with other lines of evidence, these results suggest that the African lineage represented by *Systates*, *Dicasticus* and *Syntaphocerus* is most likely to represent the sister clade to Ectemnorhinini weevils.

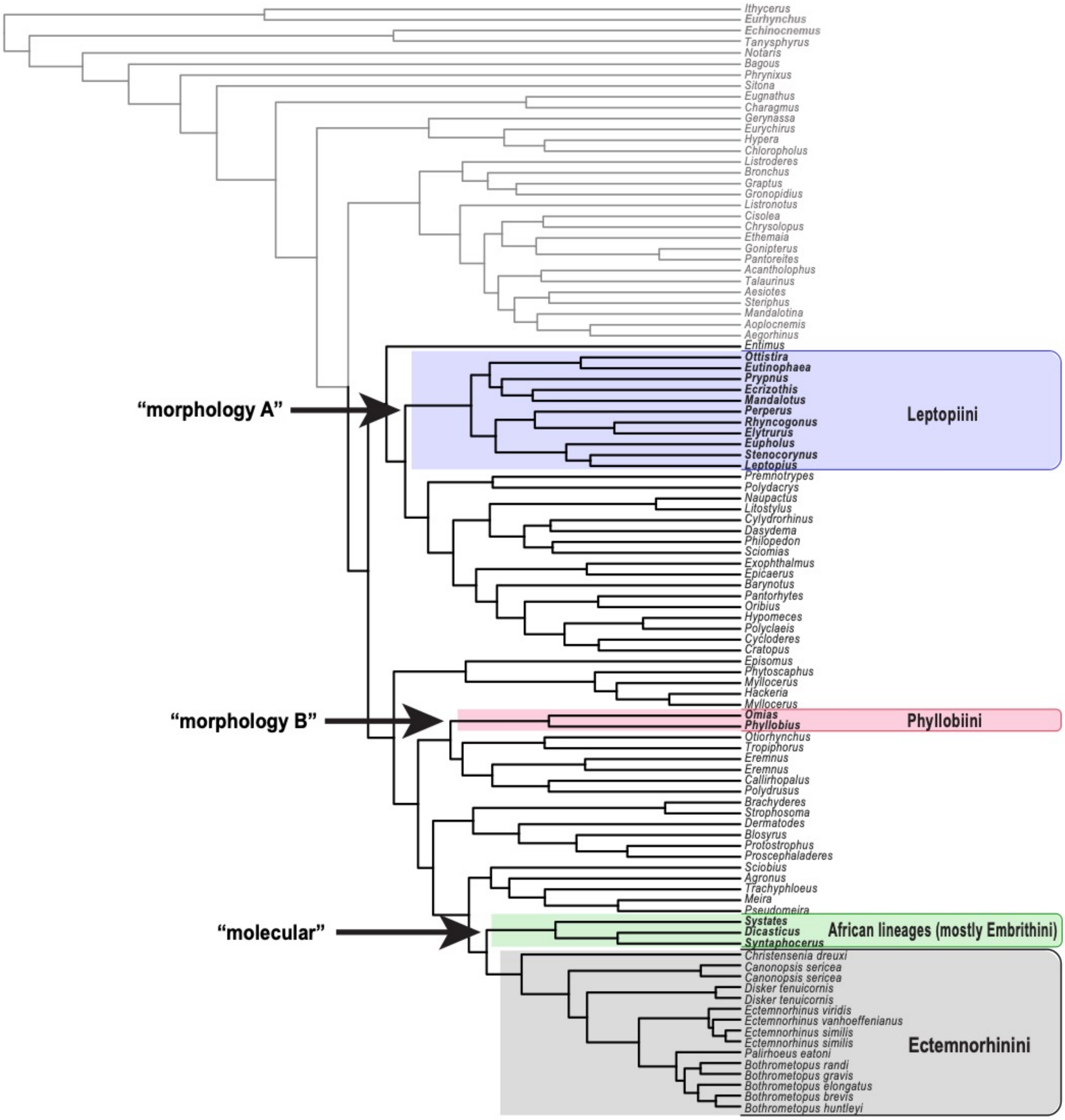

Figure S2 Alternative topological hypotheses (regarding sister relationship to the Ectemnorhinini) tested using marginal likelihood estimation. The Bayesian phylogeny generated from 515 nuclear protein-coding genes is shown (as per Figures 1, S1), with all weevil taxa outside the focal Entiminae greyed out for clarity. The three colored boxes indicate the three hypotheses tested (see Text S1); in each case the highlighted clade was constrained as monophyletic with the Ectemnorhinini (thus enforcing a sister relationship), yet all interspecific relationships within and outside this monophyletic group were allowed to vary freely.

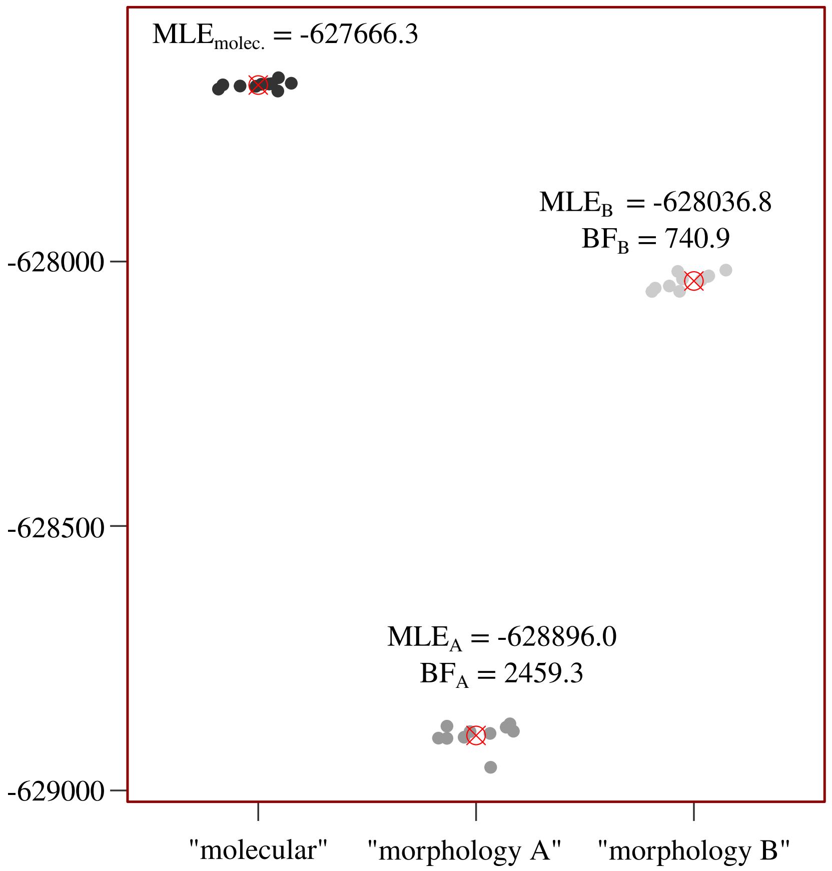

Figure S3 Outcomes from phylogenetic model selection analyses for testing the nearest extant relatives of the Ectemnorhinini (see Text S1). Distributions of marginal likelihood estimates across 10 replicates of stepping-stone analysis are indicated by filled circles, with mean marginal likelihood estimates shown as red targets for the three hypotheses: “molecular” (left), “morphology A” (center) and “morphology B” (right). Mean marginal likelihood estimators are also provided for each model, and Bayes factors (i.e., BF_A and BF_B) are shown for the two alternative hypotheses (“morphology A” and “morphology B”).

ii. Designation of Molecular Operational Taxonomic Units (MOTUs)

Text S2: MOTU designation criteria

Preliminary Maximum Likelihood (ML) and Bayesian Inference (BI) phylogenetic analyses revealed that molecular data did not consistently support traditional morphological distinctions for Ectemnorhinini (see Figure S4). As the morphological taxonomy of the group has long been controversial, we designated new species units (MOTUs) based on the phylogenetic data.

Morphological species were split when:
morphospecies consisted of two or more monophyletic subsets (clades) that each occur exclusively on a different island and were separated by 100% bootstrap support (ML) and 1.0 posterior probability (BI), thus suggesting ongoing genetic exchange is highly unlikely.

Morphological species were combined when:
morphospecies occurred in paraphyly on either the ML or BI tree (having low support where they were not in paraphyly), yet formed a monophyletic clade when combined with a second morphospecies (with which they were confirmed to have very few, if any, reliable morphological distinctions).

MOTUs resulting from these criteria more closely met the assumption that tree tips represent minimum, genetically exchanging populations, as required for subsequent analyses (i.e. molecular dating in BEAST and biogeographical inference in BioGeoBEARS). Similar criteria have been applied to other weevil radiations (7).

The new MOTU codes and the morphological species/localities to which they apply are provided in Table S1 and Figure S4 below.

Table S1 Molecular operational taxonomic units (MOTUs) designated phylogenetically for the Ectemnorhinini tribe of weevils, based on Maximum Likelihood and Bayesian Inference analysis of five genes. Purple MOTUs have resulted from the combining of morphospecies, green MOTUs from the splitting of morphospecies (as per Text S2).

Genus	MOTU code	Species (locality)
Bothrometopus	Ban	*B. angusticollis*
	Bde	*B. desolationis*
	Bda	*B. daviesi*
	Bbr*	*B. brevis* + *B. cf. brevis*
	Bcr-Bpa	*B. crozetensis* + *B. parvulus* (Marion Island clade)
	Bpa(P)	*B. parvulus* (Prince Edward Island clade)
	Bel	*B. elongatus*
	Bfa	*B. fasciatus*
	Bgr(K)	*B. gracilipes* (Kerguelen Island clade)
	Bgr(H)	*B. gracilipes* (Heard Island clade)
	Bgv	*B. gravis*
	Bhu(M)	*B. huntleyi* (Marion Island clade)
	Bhu(P)	*B. huntleyi* (Prince Edward Island clade)
	Bra(C)[#]	*B. randi* (Possession Island clade)
	Bra(M)	*B. randi* (Marion Island clade)
Christensenia	Can	*C. antarctica*
	Cdr	*C. dreuxi*
Canonopsis	Cse	*C. sericeus*
Disker	Dte	*D. tenuicornis*
Ectemnorhinus	Edr	*E. drygalskii*
	Ege	*E. geniculatus*
	Epo-Eva	*E. possessionensis* + *E. vanhoeffenianus*
	Eri	*E. richtersi*
	Esi	*E. similis*
	Evi	*E. viridis*
Palirhoeus	Pea(PEI)^	*P. eatoni* (Prince Edward + Marion islands clade)
	Pea^	*P. eatoni* (all other islands)

* Note that the genetic (and morphological) similarity of *B. cf. brevis* specimens from Possession Island to *B. brevis* most likely indicates a new locality record for *B. brevis* on Possession Island

[#] This clade was given the suffix “C” as Possession Island represents the Crozet archipelago

^ This split was also supported by SNP data, which provided strong evidence of negligible gene flow between these clades

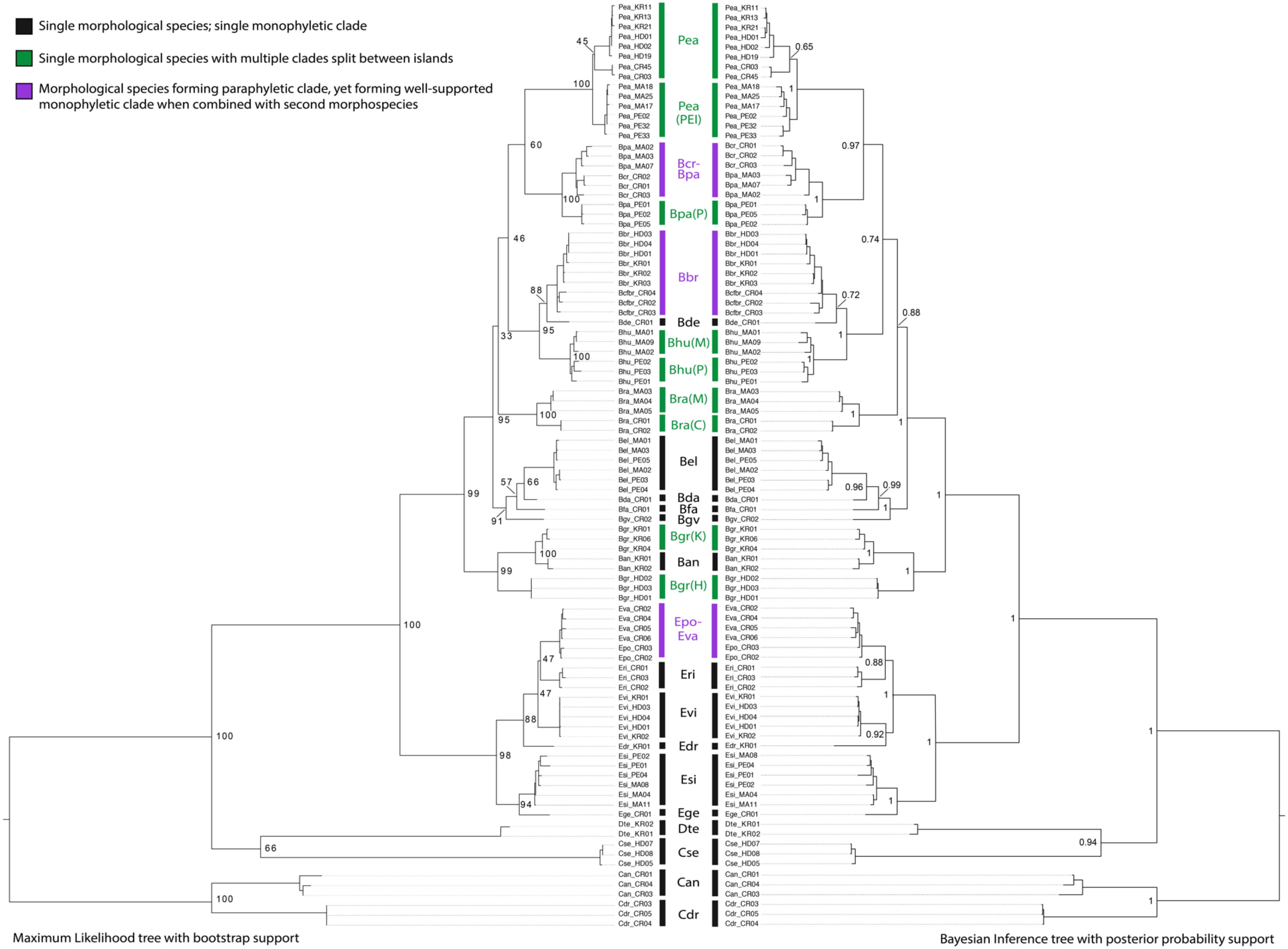

Figure S4 Maximum Likelihood (ML) and Bayesian Inference (BI) phylogenetic trees for the Ectemnorhinini, based on an 'RY-coded' concatenated dataset of three mitochondrial and two nuclear genes. Topology was broadly congruent between the two analytical techniques. MOTUs are delineated in the center (see Text S2 for criteria). ML bootstrap support / BI posterior probabilities are provided on nodes. Analyses based on original sequences (without RY-coding) also supported these designations. Raw tree files in Newick format are provided on Figshare (doi:10.26180/14446023).

iii. Supplementary phylogenetic results

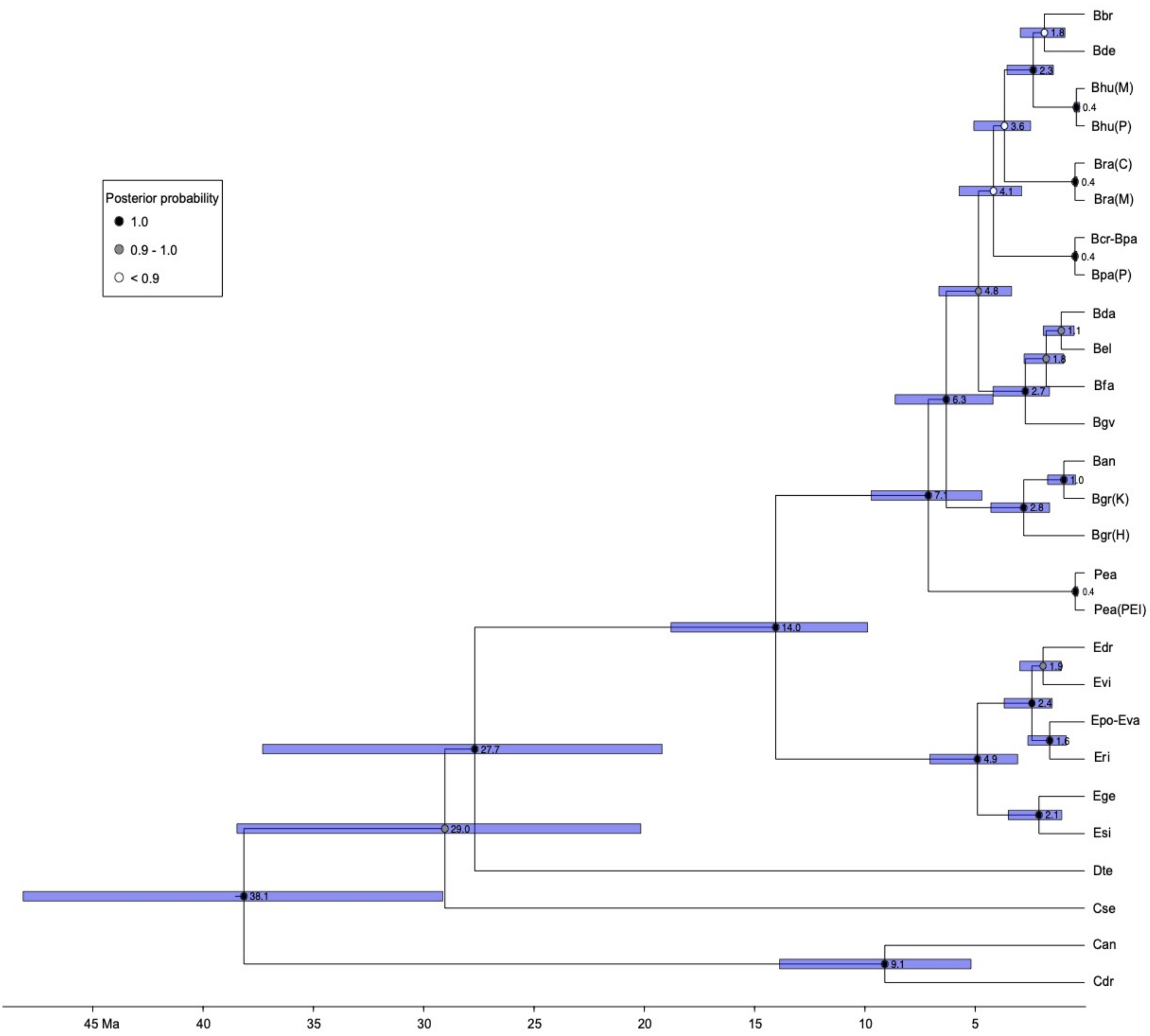

Figure S5 Maximum clade credibility tree for Ectemnorhinini generated in BEAST using data from three mitochondrial and two nuclear genes. The tree was calibrated with the geological emergence of the Prince Edward Islands archipelago and two key divergence dates estimated by our fossil-calibrated phylogenomic tree (see Text S7). Tips are labelled with MOTU codes (see section ii); nodes are labelled with estimated node ages (in Ma). Error bars represent 95% highest posterior densities for estimated node age. Posterior probability (PP) is indicated by node shading; 69% of nodes had PP = 1.0; 19% had PP = 0.90-0.99, and 12% had PP = 0.5-0.9. This phylogeny was used for subsequent historical biogeographic analyses and a simplified version is also shown in Figure 2, main text. The tree is also provided in Newick format on Figshare (doi:10.26180/14446023).

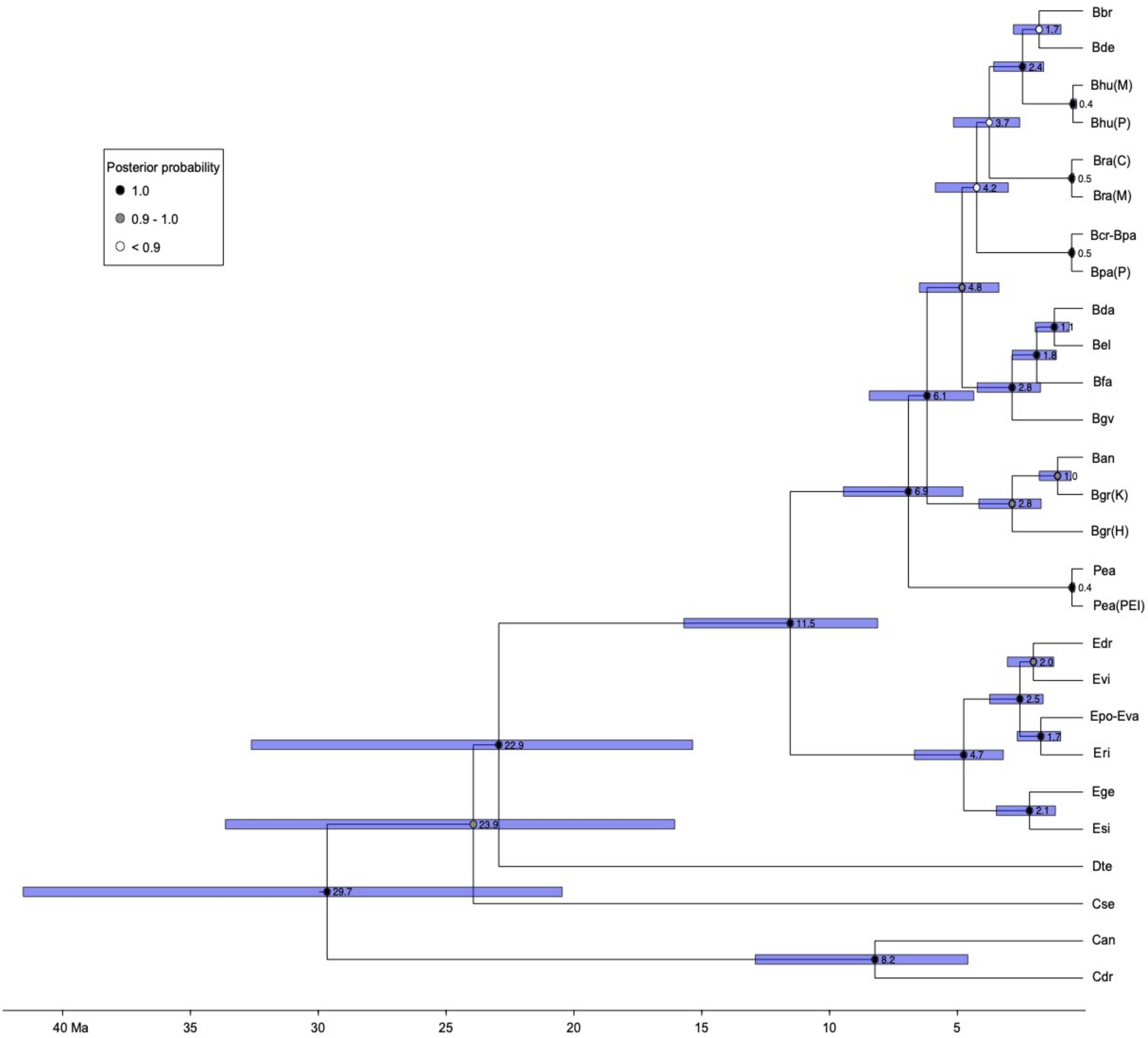

Figure S6 Maximum clade credibility tree for Ectemnorhinini generated in BEAST using data from three mitochondrial and two nuclear genes. The tree was calibrated with the geological emergence of the Prince Edward Islands archipelago and substitution rates from the Coleoptera literature (see Text S7). Tips are labelled with MOTU codes (see section ii); nodes are labelled with estimated node ages (in Ma). Error bars represent 95% highest posterior densities for estimated node age. Posterior probability is indicated by node shading.

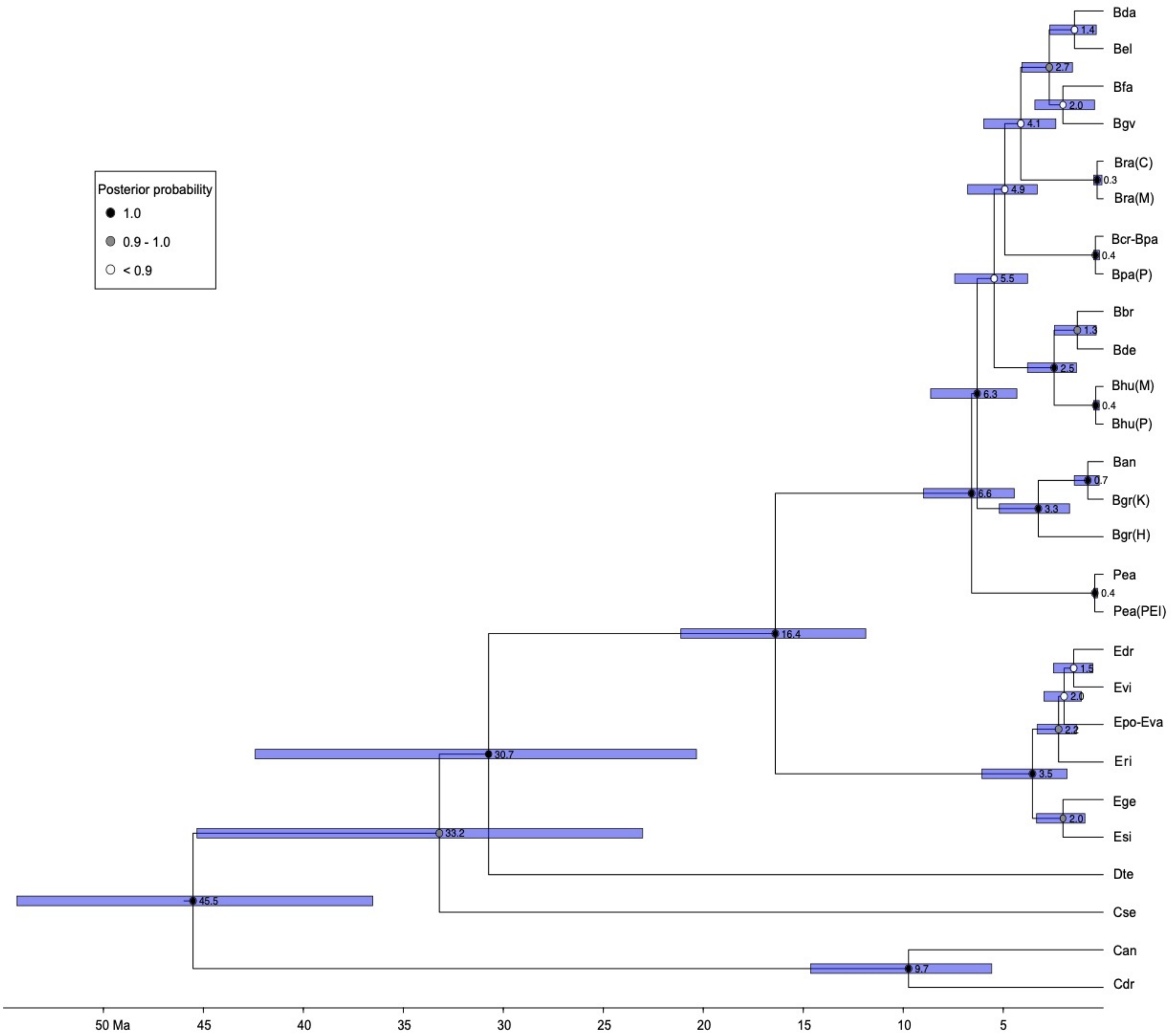

Figure S7 Maximum clade credibility tree for Ectemnorhinini generated in starBEAST using data from three mitochondrial and two nuclear genes (gene trees co-estimated in a shared species tree). The tree was calibrated with the geological emergence of the Prince Edward Islands archipelago and two key divergence dates estimated by our fossil-calibrated phylogenomic tree (see Text S7). Tips are labelled with MOTU codes (see section ii); nodes are labelled with estimated node ages (in Ma). Error bars represent 95% highest posterior densities for estimated node age. Posterior probability is indicated by node shading.

iv. Diversification analyses

Table S2 Bayes factors for different models of diversification rate heterogeneity estimated in BAMM as compared to the null model (no diversification rate shifts), using the dated phylogeny shown in Figure S5 and an input file of sampling proportions for each genus (deduced from Table S13) to account for sampling bias. The most highly supported model is shown in bold.

No. of rate shifts	Bayes factor
0	1
**1**	**81.9**
2	59.3
3	38.2
4	25.3
5	14.7
6	9.5
7	7.3
8	3.6
10	7.3

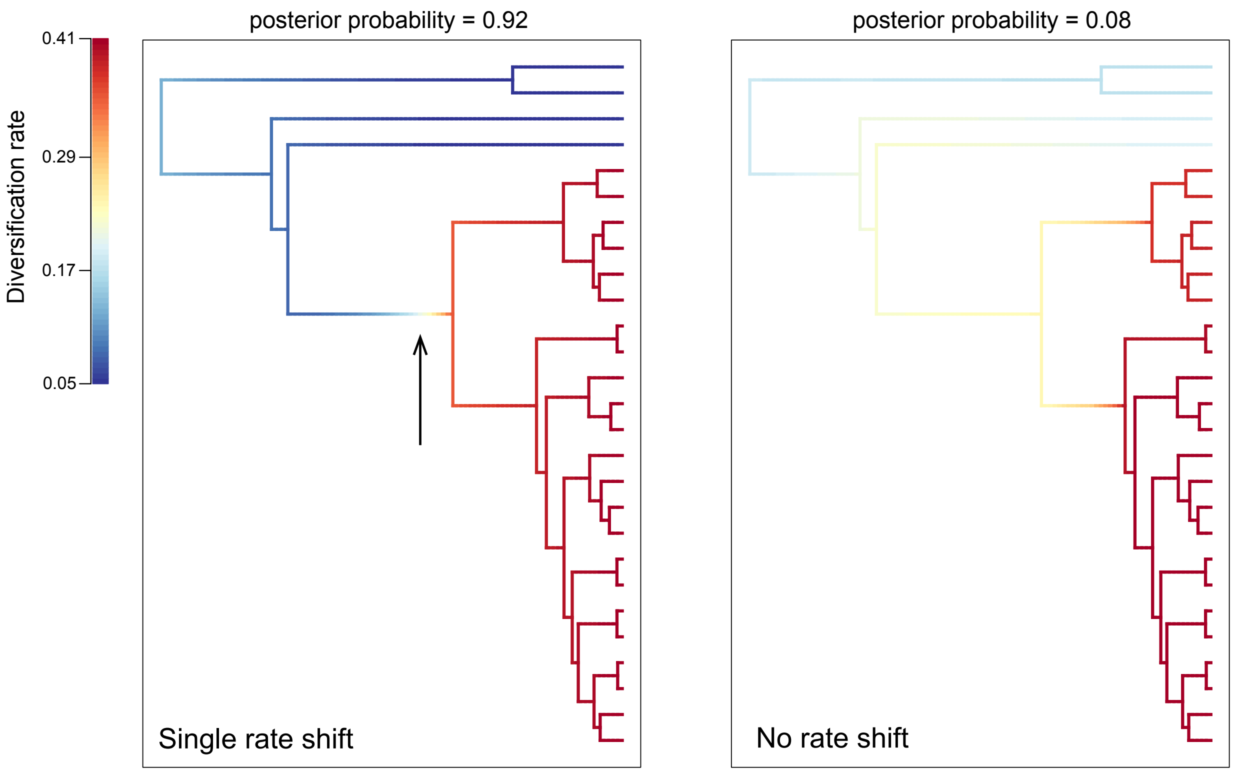

Figure S8 The 95% credible set of distinct diversification rate shift configurations, as estimated in BAMM using the dated phylogeny shown in Figure S5 and an input file of sampling proportions for each genus (deduced from Table S13) to account for sampling bias. Posterior probability is shown above each plot; a single rate shift (acceleration) received much stronger support than no rate shifts (0.92 versus 0.08 posterior probability).

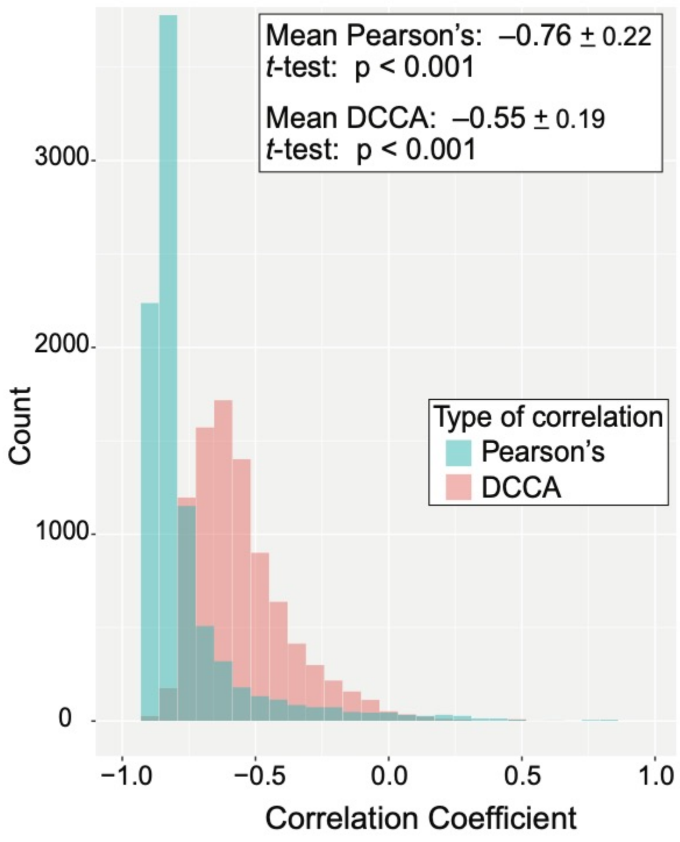

Figure S9 Correlation coefficients (both Pearson's and detrended cross-correlation analysis ('DCCA')) for the correlation between speciation rate and paleotemperature (using climate data smoothed in 20,000-year windows: see ref. 8). The analysis was run for all 9,000 simulations of speciation rate estimated by BAMM. The distribution of coefficients was tested for a significant departure from zero using a Student's *t*-test; in both cases, a significant negative correlation was detected.

Table S3 Outcomes of diversification model selection using RPANDA. The highest supported model is shown in bold, while the models that fall within △AIC < 2 (thus should all be considered to have reasonable support) are highlighted (note however that we do not consider a constant-rate model biologically realistic). For each matching pair of models which only differ in terms of whether they are time- or temperature-dependent, the temperature-dependent model always received higher support.
That the exponential function was consistently negative indicates an increase in speciation rate with decreasing temperature / decreasing time (toward the present).

MODEL	AICc	△AIC	AIC weight	Exponential function (rate of change of speciation rate with time/temperature)
**TEMP-dependent: speciation variable, no extinction**	**147.766**	**0.000**	**31%**	**-0.306**
Constant speciation, constant extinction	148.574	0.808	20%	0
TIME-dependent: speciation variable, no extinction	149.012	1.247	16%	-0.105
TEMP-dependent: speciation variable, constant extinction	149.916	2.150	10%	-0.207
TEMP-dependent: constant speciation, extinction variable	150.604	2.838	7%	0
TIME-dependent: constant speciation, extinction variable	151.105	3.340	6%	0
TIME-dependent: speciation variable, constant extinction	151.556	3.790	5%	-0.105
TEMP-dependent: speciation variable, extinction variable	152.692	4.927	3%	-0.184
TIME-dependent: speciation variable, extinction variable	153.792	6.027	2%	-0.052
Constant speciation, no extinction	163.889	16.123	0%	0

v. Historical biogeography inference

Table S4 Outcomes of historical biogeographic inference in BioGeoBEARS using the Dispersal-Extinction-Cladogensis (DEC) model, the DEC+*J* model and the DEC+*J*+*X* model, as well as the mean number of cladogenetic and anagenetic events inferred from 100 iterations of biogeographic stochastic mapping (BSM).
LnL = log-likelihood; *d* = dispersal parameter; *e* = extinction parameter; *J* = founder effect parameter; *X* = dispersal distance modifier

**Model outcomes**						**Mean event counts (from BSM)**				
						Anagenetic	Cladogenetic			
Model	LnL	*d*	*e*	*J*	*X*	Range expansion	Founder event	Within-archipelago sympatry	Subset sympatry	Vicariance
DEC	-85.99	0.08	0.05	n/a	n/a	27	0	15	6	5
DEC+*J*	-77.41	0.05	0.03	0.16	n/a	18	8	12	4	2
DEC+*J*+*X*	-74.47	0.24	0.03	0.59	-1.96	18	7	15	3	2

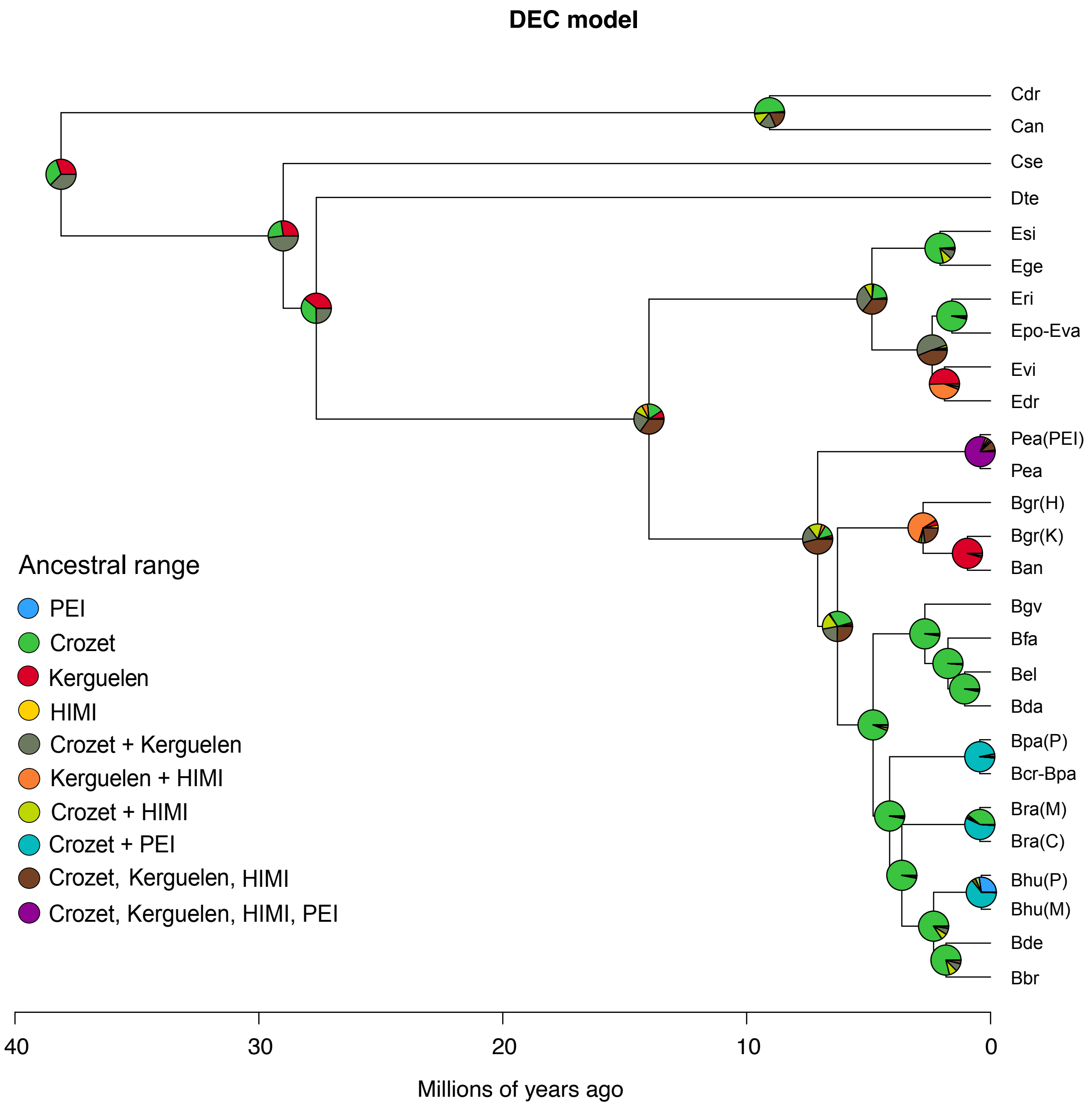

Figure S10 Ancestral range estimation based on the DEC biogeographic model implemented in BioGeoBEARS. Pie charts show the probability of ancestral ranges for each ancestral node throughout the phylogeny. Phylogeny tips are labelled with MOTU codes.

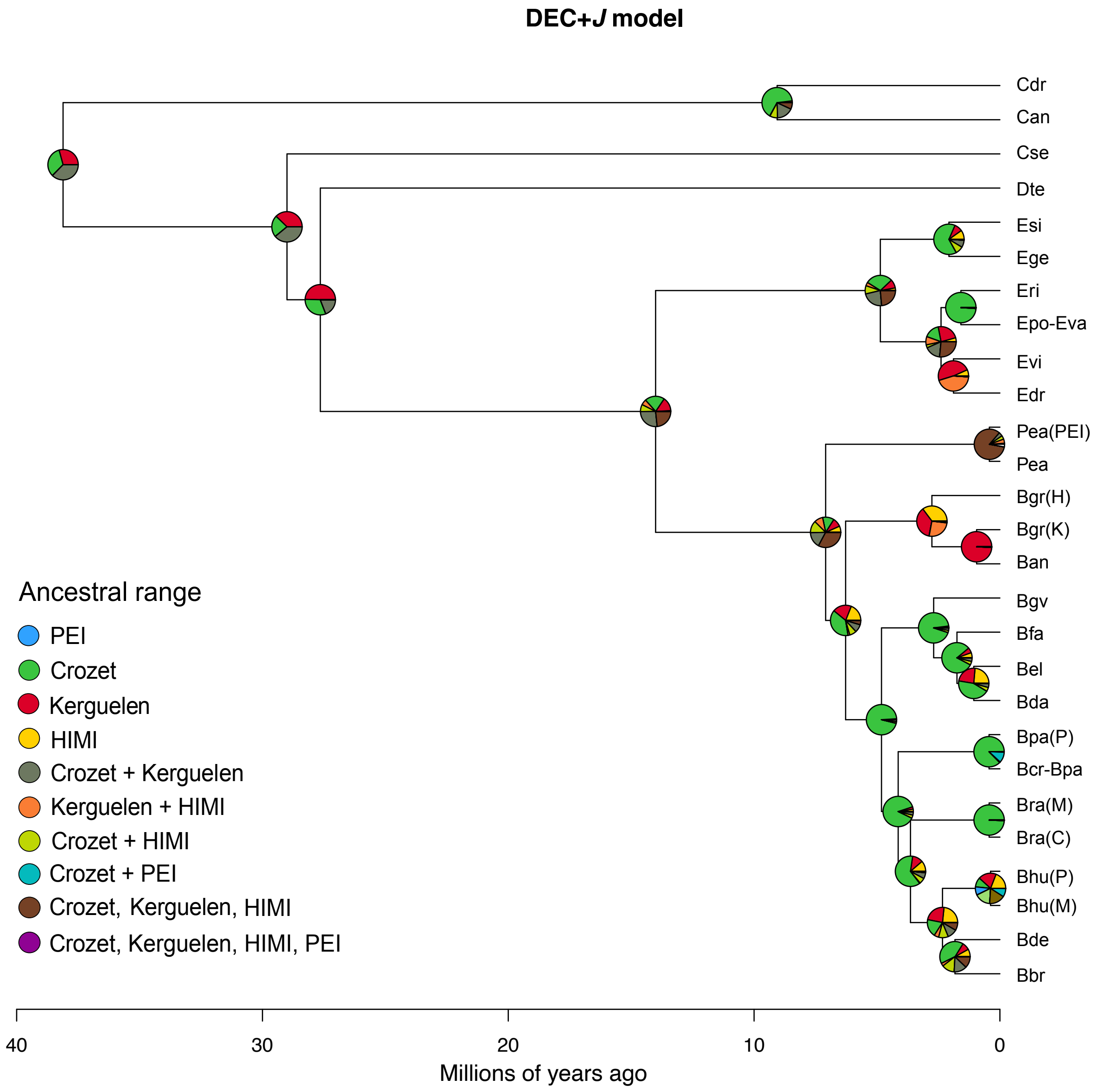

Figure S11 Ancestral range estimation based on the DEC+*J* biogeographic model implemented in BioGeoBEARS. Pie charts show the probability of ancestral ranges for each ancestral node throughout the phylogeny. Phylogeny tips are labelled with MOTU codes.

Table S5 Mean number of total inter-archipelago dispersal events (both anagenetic and cladogenetic) inferred throughout the Ectemnorhinini evolutionary history based on the DEC (dispersal-extinction-cladogenesis) model and 100 iterations of biogeographic stochastic mapping. Dispersal events inferred in a westerly direction are highlighted in yellow. HIMI = Heard Island and McDonald Islands; K = Kerguelen Islands; C = Crozet Islands; PEI = Prince Edward Islands

		Dispersal into archipelago				
		HIMI	K	C	PEI	*Mean total dispersals out of archipelago:*
Dispersal from archipelago	HIMI	0	2	1.4	0.6	**4**
	K	3.5	0	3.9	0.6	**8**
	C	4.5	5.6	0	5	**15.1**
	PEI	0	0	0	0	**0**
	Mean total dispersals into archipelago:	**8**	**7.6**	**5.3**	**6.2**	**Mean total dispersal events ≈ 27**

Table S6 Mean number of total inter-archipelago dispersal events (both anagenetic and cladogenetic) inferred throughout the Ectemnorhinini evolutionary history based on the DEC+*J* model and 100 iterations of biogeographic stochastic mapping. Dispersal events inferred in a westerly direction are highlighted in yellow.
HIMI = Heard Island and McDonald Islands; K = Kerguelen Islands; C = Crozet Islands; PEI = Prince Edward Islands

Dispersal from archipelago		Dispersal into archipelago				
		HIMI	K	C	PEI	*Mean total dispersals out of archipelago:*
	HIMI	0	1.7	1.2	1.0	**3.9**
	K	3.8	0	3.7	1.1	**8.6**
	C	3.2	4.8	0	5.3	**13.3**
	PEI	0	0	0	0	**0**
	Mean total dispersals into archipelago:	**7**	**6.5**	**4.9**	**7.4**	**Mean total dispersal events ≈ 26**

Table S7 Mean number of total inter-archipelago dispersal events (both anagenetic and cladogenetic) inferred throughout the Ectemnorhinini evolutionary history based on the DEC+*J*+*X* model and 100 iterations of biogeographic stochastic mapping. Dispersal events inferred in a westerly direction are highlighted in yellow.
HIMI = Heard Island and McDonald Islands; K = Kerguelen Islands; C = Crozet Islands; PEI = Prince Edward Islands

Dispersal from archipelago	Dispersal into archipelago: HIMI	K	C	PEI	*Mean total dispersals out of archipelago:*
HIMI	0	3.2	0.7	0.1	**4**
K	5.8	0	2.2	0.3	**8.3**
C	1.6	3.7	0	7	**12.3**
PEI	0	0	0	0	**0**
Mean total dispersals into archipelago:	**7.4**	**6.9**	**2.9**	**7.4**	**Mean total dispersal events ≈ 25**

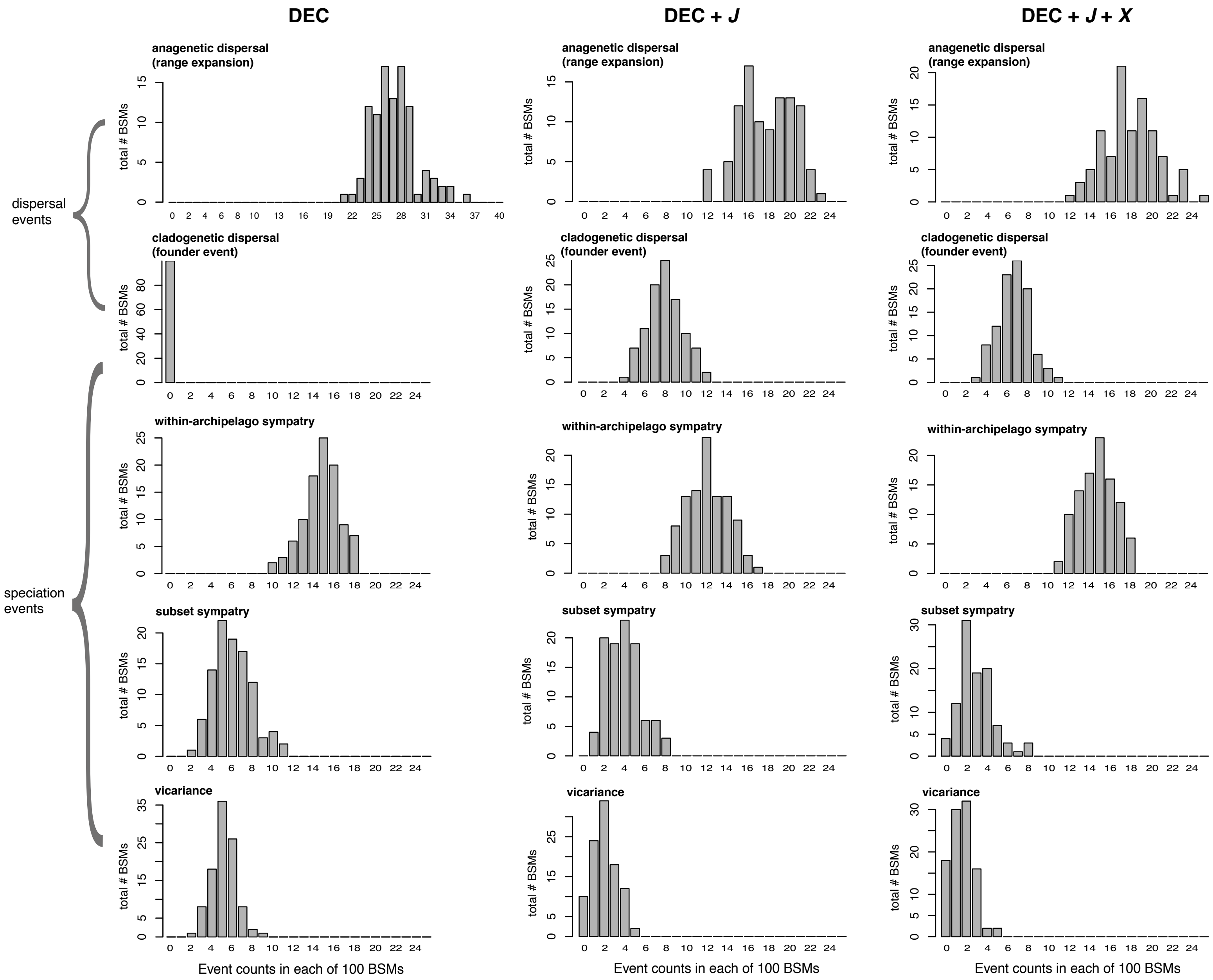

Figure S12 Frequency distributions of key historical biogeographic events throughout the Ectemnorhinini phylogeny (based on the DEC, DEC+*J* and DEC+*J*+*X* models), inferred by 100 iterations of biogeographic stochastic mapping (BSM).

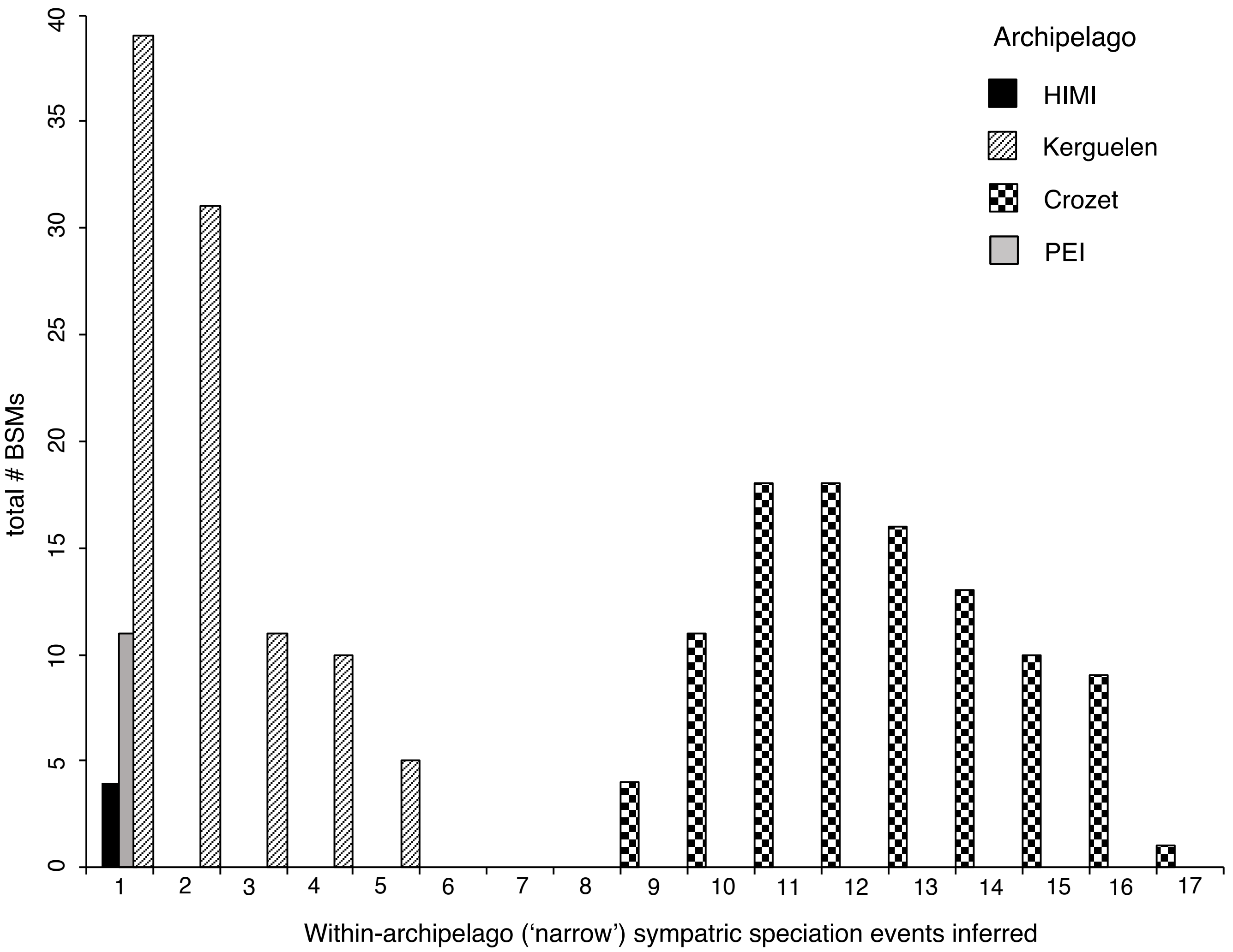

Figure S13 Frequency distribution of within-archipelago speciation events inferred throughout the Ectemnorhinini evolutionary history based on 100 iterations of biogeographic stochastic mapping (BSM) using the DEC+*J*+*X* model. Note that for the HIMI and PEI archipelagos, most iterations of BSM inferred zero events (not shown on chart). Mean estimates for each archipelago were: Heard Island and McDonald Islands (HIMI) = 0; Kerguelen = 2; Crozet = 13; Prince Edward Islands (PEI) = 0.

Table S8 Matrix of pairwise F_{ST} values among sites for *Palirhoeus eatoni*, based on a dataset of 5,859 SNPs. Site codes correspond to those detailed in Table S14 (capital letters code for island, while lower-case suffixes denote sites within islands). F_{ST} values have been color-coded according to the legend provided. The clear 'east'–'west' split between Marion and Prince Edward islands (PEI archipelago) and all other islands is outlined with a dashed line.

F_{ST} legend
0–0.120
0.121–0.240
0.241–0.480
0.481–0.600
0.601–0.720
0.721–0.840
0.841–0.960

F_{ST}	HD	KR-a	KR-b	KR-c	PO-a	PO-b	PO-c	MA	PE-a	PE-b
HD	-									
KR-a	0.447	-								
KR-b	0.284	0.259	-							
KR-c	0.301	0.374	0.168	-						
PO-a	0.545	0.454	0.499	0.468	-					
PO-b	0.622	0.505	0.560	0.519	0.089	-				
PO-c	0.651	0.534	0.588	0.562	0.139	0.068	-			
MA	0.873	0.870	0.860	0.860	0.796	0.827	0.836	-		
PE-a	0.871	0.862	0.851	0.849	0.766	0.800	0.812	0.046	-	
PE-b	0.875	0.868	0.855	0.853	0.767	0.802	0.815	0.026	0.041	-

Table S9 Matrix of pairwise fixed differences among populations of *Palirhoeus eatoni*, shown as the number of SNPs fixed for alternative alleles (a), and the percentage of the entire dataset (5,859 SNPs) fixed for alternative alleles (b). Site codes correspond to those provided in Table S14 (capital letters code for island, lower-case suffixes for sites within islands). The clear 'east'–'west' split between Marion and Prince Edward islands (PEI archipelago) and all other islands is outlined with a dashed line.

(a)

	HD	KR-a	KR-b	KR-c	PO-a	PO-b	PO-c	MA	PE-a	PE-b
HD	-									
KR-a	32	-								
KR-b	3	2	-							
KR-c	8	14	1	-						
PO-a	115	87	61	65	-					
PO-b	186	141	106	110	0	-				
PO-c	195	153	117	124	1	1	-			
MA	1920	1951	1691	1796	1109	1225	1278	-		
PE-a	1880	1904	1652	1754	1085	1197	1238	0	-	
PE-b	2009	2031	1760	1871	1171	1291	1335	0	0	-

(b)

	HD	KR-a	KR-b	KR-c	PO-a	PO-b	PO-c	MA	PE-a	PE-b
HD	-									
KR-a	1%	-								
KR-b	0%	0%	-							
KR-c	0%	0%	0%	-						
PO-a	2%	1%	1%	1%	-					
PO-b	3%	2%	2%	2%	0%	-				
PO-c	3%	3%	2%	2%	0%	0%	-			
MA	33%	33%	29%	31%	19%	21%	22%	-		
PE-a	32%	33%	28%	30%	19%	20%	21%	0%	-	
PE-b	34%	35%	30%	32%	20%	22%	23%	0%	0%	-

Figure S14 Plot generated by Bayesian clustering analysis in fastSTRUCTURE, showing the three main clusters identified across all populations of *Palirhoeus eatoni.*

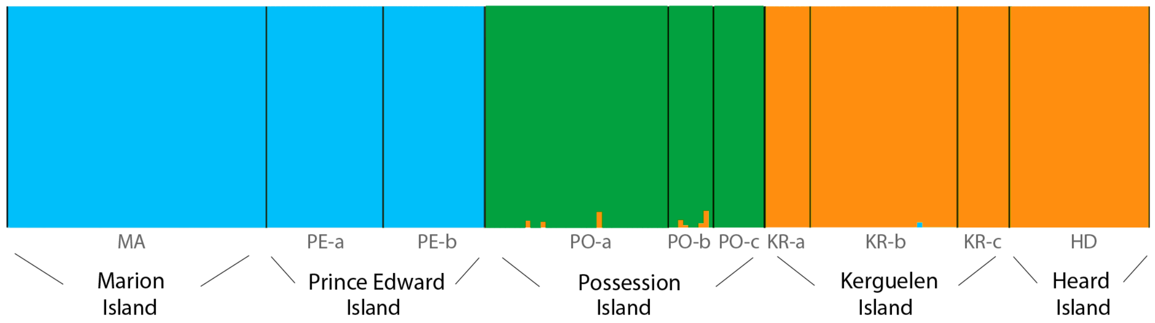

Table S10 Outcomes for partial Mantel tests for *Palirhoeus eatoni.* Genetic distance (F_{ST}) correlated significantly with both the geographic distance between sites and the identified clusters, regardless of whether two clusters (i.e. 'east' and 'west') or three clusters (i.e. as observed in Figure S14) were considered.

Relationship tested	Mantel's *r*	p-value
Genetic distance & geographic distance (correcting for 2 clusters)	0.756	0.002
Genetic distance & geographic distance (correcting for 3 clusters)	0.460	0.014
Genetic distance & 2 clusters (correcting for geographic distance)	0.768	0.002
Genetic distance & 3 clusters (correcting for geographic distance)	0.598	0.003

Table S11 Genetic diversity statistics for populations of *Palirhoeus eatoni* based on a dataset of 5,859 SNPs. None of the populations were found to be significantly different with respect to any of these genetic diversity metrics, based on 5000 bootstraps over loci. 95% confidence intervals are provided in parentheses.
H_O = observed heterozygosity; H_E = expected heterozygosity; F_{IS} = inbreeding coefficient.

Island	Site	H_O	H_E	F_{IS}
Heard	HD	0.044 (0.041, 0.047)	0.064 (0.060, 0.068)	0.309 (0.285, 0.331)
Kerguelen	KR-a	0.064 (0.060, 0.068)	0.072 (0.068, 0.076)	0.110 (0.084, 0.137)
	KR-b	0.087 (0.083, 0.092)	0.088 (0.084, 0.092)	0.007 (0, 0.023)
	KR-c	0.084 (0.080, 0.089)	0.090 (0.085, 0.094)	0.061 (0.040, 0.082)
Possession	PO-a	0.127 (0.123, 0.132)	0.163 (0.158, 0.168)	0.219 (0.206, 0.233)
	PO-b	0.125 (0.120, 0.130)	0.171 (0.165, 0.176)	0.267 (0.250, 0.284)
	PO-c	0.118 (0.113, 0.123)	0.149 (0.144, 0.154)	0.206 (0.187, 0.224)
Marion	MA	0.065 (0.062, 0.069)	0.079 (0.075, 0.083)	0.173 (0.159, 0.187)
Prince Edward	PE-a	0.074 (0.070, 0.078)	0.086 (0.082, 0.090)	0.140 (0.124, 0.157)
	PE-b	0.071 (0.067, 0.075)	0.082 (0.078, 0.087)	0.139 (0.123, 0.155)

vii. Physiological data

Text S3: Physiological evidence supporting zoochory of Ectemnorhinini weevils by seabirds

On sub-Antarctic islands lacking introduced rodent and beetle predators, such as Heard Island and Prince Edward Island, densities of insects are much higher than on those islands with predators, and the situation changes through time as predators significantly reduce their prey populations (9).

High densities of weevils mean that they often crawl onto flighted seabirds and could readily be transported. Survival of such transport (assuming the insects avoid preening by birds, which is unlikely during continuous flight) depends on the metabolic and water resources of the insects and their metabolic rates. If individuals are exposed to relatively moderate temperatures, they can survive depletion of resources and water for some time (10).

For the Ectemnorhinini weevils, data exist on metabolic rates at a range of temperatures, dehydration tolerances and body masses (11, 12). The latter enable estimation of metabolic resources (13). Based on an assumption of albatross external temperatures of 30°C (14) and these weevil physiological and body-mass data, an ectemnorhinine weevil of average body size (18 mg) could survive on average for 9 days before death from dehydration and 34 days before death from resource depletion (calculations available on request from the authors). Individuals of the wandering albatross can cover 900 km in a single day (15), approximately the distance between the Prince Edward Islands and the Crozet archipelago.

viii. Specimen details and sequence data deposition

Table S12 List of all weevil species used for phylogenomic analysis, with those of the Ectemnorhinini shown in bold. Tribal and subfamilial classification is as used in the latest literature, not as resolved here (see Figure 1). DNA codes relate to Anchored Hybrid Enrichment data deposited on the Zenodo Digital Repository (10.5281/zenodo.3955188). The final number of loci sequenced for each specimen is also shown.

DNA code	Genus	Species	Tribe	Subfamily	Final no. of loci	Notes
DDM0520	*Acantholophus*	*sp.*	Amycterini	Cyclominae	387	Previously published in ref. 16
DDM2528	*Aegorhinus*	*silvicola*	Aterpini	Cyclominae	206	
DDM0076	*Aesiotes*	*notabilis*	Aterpini	Cyclominae	430	Previously published in ref. 16
DDM4172	*Agronus*	*cinerarius*	Otiorhynchini	Entiminae	193	
DDM4877	*Aoplocnemis*	*sp*	Aterpini	Cyclominae	276	
DDM0507	*Bagous*	*americanus*	Bagoini	Bagoinae	435	Previously published in ref. 16
DDM4792	*Barynotus*	*obscurus*	Barynotini	Entiminae	284	
DDM4555	*Blosyrus*	*sp.*	Blosyrini	Entiminae	301	
**DDM4505**	***Bothrometopus***	***brevis***	**Ectemnorhinini**	**Entiminae**	**410**	
**DDM4507**	***Bothrometopus***	***randi***	**Ectemnorhinini**	**Entiminae**	**415**	
**DDM4509**	***Bothrometopus***	***gravis***	**Ectemnorhinini**	**Entiminae**	**428**	
**DDM4512**	***Bothrometopus***	***huntleyi***	**Ectemnorhinini**	**Entiminae**	**422**	
**DDM4514**	***Bothrometopus***	***elongatus***	**Ectemnorhinini**	**Entiminae**	**393**	
DDM4780	*Brachyderes*	*pubescens*	Brachyderini	Entiminae	212	
DDM5459	*Bronchus*	*furvus*	Hipporhinini	Cyclominae	344	
DDM4373	*Callirhopalus*	*bifasciatus*	Cneorhinini	Entiminae	233	
**DDM5479**	***Canonopsis***	***sericea***	**Ectemnorhinini**	**Entiminae**	**373**	
**DDM5480**	***Canonopsis***	***sericea***	**Ectemnorhinini**	**Entiminae**	**246**	
DDM4732	*Charagmus*	*stierlini*	Sitonini	Sitoninae	393	
DDM4384	*Chloropholus*	*sp.*	Hyperini	Hyperinae	354	
**DDM4511**	***Christensenia***	***dreuxi***	**Ectemnorhinini**	**Entiminae**	**360**	
DDM4882	*Chrysolopus*	*spectabilis*	Aterpini	Cyclominae	212	
DDM4893	*Cisolea*	*sp*	Phrynixini		311	
DDM5401	*Cratopus*	*sp.*	Cratopodini	Entiminae	371	
DDM4793	*Cycloderes*	*guinardi*	Tanymecini	Entiminae	317	
DDM2524	*Cylydrorhinus*	*chilensis*	Cylydrorhinini	Entiminae	209	
DDM4180	*Dasydema*	*anucella*	Leptopiini	Entiminae	134	
DDM4937	*Dermatodes*	*sp*	Dermatodini	Entiminae	277	
DDM3063	*Dicasticus*	*funicularis*	Embrithini	Entiminae	253	
**DDM5481**	***Disker***	***tenuicornis***	**Ectemnorhinini**	**Entiminae**	**351**	
**DDM5482**	***Disker***	***tenuicornis***	**Ectemnorhinini**	**Entiminae**	**395**	
DDM0509	*Echinocnemus*	*sp.*	Erirhinini	Erirhininae	420	Previously published in ref. 16
DDM5363	*Ecrizothis*	*boviei*	Leptopiini	Entiminae	384	
**DDM4506**	***Ectemnorhinus***	***vanhoeffenianus***	**Ectemnorhinini**	**Entiminae**	**425**	

**DDM4508**	***Ectemnorhinus***	***viridis***	**Ectemnorhinini**	**Entiminae**	**363**	
**DDM4513**	***Ectemnorhinus***	***marioni***	**Ectemnorhinini**	**Entiminae**	**350**	
**DDM5370**	***Ectemnorhinus***	***similis***	**Ectemnorhinini**	**Entiminae**	**403**	
DDM5079	*Elytrurus*	*expansus*	Elytrurini	Entiminae	308	
DDM5404	*Entimus*	*imperialis*	Entimini	Entiminae	423	
DDM4406	*Epicaerus*	*formidolosus*	Geonemini	Entiminae	114	
DDM3121	*Episomus*	*sp*	Episomini	Entiminae	287	
DDM3075	*Eremnus*	*sp*	Tanyrhynchini	Entiminae	333	
DDM5451	*Eremnus*	*acuminatus*	Tanyrhynchini	Entiminae	224	
DDM5368	*Ethemaia*	*sellata*	Rhythirrinini	Cyclominae	329	
DDM5076	*Eugnathus*	*sp*	Sitonini	Sitoninae	384	
DDM4360	*Eupholus*	*geoffreyi*	Leptopiini	Entiminae	321	
DDM0491	*Eurhynchus*	*laevior*	Eurhynchini	Eurynchinae	440	Previously published in ref. 16
DDM4913	*Eurychirus*	*bituberculatus*		Hyperinae	375	
DDM4963	*Eutinophaea*	*sp*	Ottistirini	Entiminae	352	
DDM4394	*Exophthalmus*	*sp.*	Eustylini	Entiminae	212	
DDM4914	*Gerynassa*	*sp*		Hyperinae	280	
DDM0078	*Gonipterus*	*notographus*	Gonipterini	Cyclominae	386	Previously published in ref. 16
DDM4787	*Graptus*	*nictitans*	Byrsopagini	Entiminae	338	
DDM4737	*Gronopidius*	*jekeli*	Hipporhinini	Cyclominae	360	
DDM4931	*Hackeria*	*sp*	Cyphicerini	Entiminae	342	
DDM0538	*Hypera*	*nigrirostris*	Hyperini	Hyperinae	415	Previously published in ref. 16
DDM4454	*Hypomeces*	*sp.*	Tanymecini	Entiminae	333	
DDM0492	*Ithycerus*	*noveboracensis*		Ithycerinae	423	Previously published in ref. 16
DDM0080	*Leptopius*	*sp.*	Leptopiini	Entiminae	396	Previously published in ref. 16
DDM0521	*Listroderes*	*bruchi*	Listroderini	Cyclominae	364	Previously published in ref. 16
DDM5111	*Listronotus*	*bonariensis*	Listroderini	Cyclominae	402	
DDM4131	*Litostylus*	*strangulatus*	Naupactini	Entiminae	276	
DDM4888	*Mandalotina*	*sp.*	Notiomimetini	Cyclominae	398	
DDM4950	*Mandalotus*	*sp*	Leptopiini	Entiminae	317	
DDM4778	*Meira*	*vauclusiana*	Peritelini	Entiminae	412	
DDM4934	*Myllocerus*	*sp.*	Cyphicerini	Entiminae	329	
DDM5441	*Myllocerus*	*undatus*	Cyphicerini	Entiminae	392	
DDM0526	*Naupactus*	*peregrinus*	Naupactini	Entiminae	368	Previously published in ref. 16
DDM4867	*Notaris*	*acridulus?*	Erirhinini	Erirhininae	329	
DDM4715	*Omias*	*puberulus*	Omiini	Entiminae	360	
DDM4928	*Oribius*	*sp*	Celeuthetini	Entiminae	285	
DDM1253	*Otiorhynchus*	*sulcatus*	Otiorhynchini	Entiminae	336	
DDM3114	*Ottistira*	*sp*	Ottistirini	Entiminae	337	
**DDM4510**	***Palirhoeus***	***eatoni***	**Ectemnorhinini**	**Entiminae**	**402**	
DDM2595	*Pantoreites*	*sp.*	Gonipterini	Cyclominae	330	
DDM5073	*Pantorhytes*	*stanleyanus*	Pachyrhynchini	Cyclominae	280	
DDM4952	*Perperus*	*sp*	Leptopiini	Entiminae	370	

DDM4930	*Philopedon*	*sp.*	Cneorhinini	Entiminae	109	
DDM5115	*Phrynixus*	*terreus*	Phrynixini		377	
DDM5074	*Phyllobius*	*sp.*	Phyllobiini	Entiminae	329	
DDM4447	*Phytoscaphus*	*sp.*	Cyphicerini	Entiminae	390	
DDM4309	*Polyclaeis*	*equestris*	Tanymecini	Entiminae	334	
DDM4130	*Polydacrys*	*moestus*	Anypotactini	Entiminae	352	
DDM0081	*Polydrusus*	*cervinus*	Polydrusini	Entiminae	442	Previously published in ref. 16
DDM4148	*Premnotrypes*	*latithorax*	Premnotrypini	Entiminae	215	
DDM5450	*Proscephaladeres*	*aspericollis*	Blosyrini	Entiminae	178	
DDM5477	*Protostrophus*	*fuscimaculatus*	Cneorhinini	Entiminae	206	
DDM4955	*Prypnus*	*sp*	Leptopiini	Entiminae	294	
DDM4790	*Pseudomeira*	*flavipennis*	Peritelini	Entiminae	272	
DDM5357	*Rhyncogonus*	*nodosus*	Rhyncogonini	Entiminae	351	
DDM4962	*Sciobius*	*marshalli*	Otiorhynchini	Entiminae	368	
DDM4229	*Sciomias*	*subtilis*	Sciaphilini	Entiminae	218	
DDM0527	*Sitona*	*hispidulus*	Sitonini	Sitoninae	433	
DDM4958	*Stenocorynus*	*sp.*	Leptopiini	Entiminae	352	
DDM4886	*Steriphus*	*sericeus*	Listroderini	Cyclominae	343	
DDM4761	*Strophosoma*	*faber*	Brachyderini	Entiminae	308	
DDM4681	*Syntaphocerus*	*hispidus*	Embrithini	Entiminae	103	
DDM3084	*Systates*	*sp*	‘Peritelini’	Entiminae	365	
DDM4903	*Talaurinus*	*sp*	Amycterini	Cyclominae	273	
DDM0071	*Tanysphyrus*	*lemnae*	Tanysphyrini	Erirhininae	422	Previously published in ref. 16
DDM4801	*Trachyphloeus*	*alternans*	Trachyphloeini	Entiminae	370	
DDM5078	*Tropiphorus*	*elevatus?*	Byrsopagini	Entiminae	293	

Table S13 Complete list of the species of the tribe Ectemnorhinini (after refs. 2, 17, 18), with collection details for all specimens used in the phylogenetics component of this study and corresponding Genbank accession details for sequences. Two species recognised by ref. 2; *Bothrometopus derelictorum* and *Ectemnorhinus pluricro*; are not included as they were treated as being conspecific with *B. crozetensis* and *E. richtersi*, respectively, in ref. 19.

			COLLECTION					SEQUENCE ACCESSIONS				
**Genus**	**Species**	**Specimen ID**	**Island (Archipelago)**	**Year**	**Latitude**	**Longitude**	**Physical deposition**[†]	**COI**	**EF1a**	**CYB**	**28S**	**16S**
Bothrometopus	*angusticollis*	Ban_KR01	Kerguelen (Kerguelen)	2016	-49.53231	70.14238	INRA (France)	MT701233	MT701413	MT701321	MT701045	MT701139
		Ban_KR02	Kerguelen (Kerguelen)	2016	-49.26966	70.04198	INRA (France)	MT701234	MT701414	MT701322	MT701046	MT701140
	brevis	Bbr_KR01	Kerguelen (Kerguelen)	2015	-49.68226	70.23741	INRA (France)	MT701238	MT701418	MT701326	MT701050	MT701144
		Bbr_KR02	Kerguelen (Kerguelen)	2011	-48.67642	69.02244	INRA (France)	MT701239	MT701419	MT701327	MT701051	MT701145
		Bbr_KR03	Kerguelen (Kerguelen)	2011	-48.84825	69.11629	INRA (France)	MT701240	MT701420	MT701328	MT701052	MT701146
		Bbr_HD01	Heard (HIMI)	2000	-53.03680	73.39743	ANIC (Australia)	MT701235	MT701415	MT701323	MT701047	MT701141
		Bbr_HD03	Heard (HIMI)	2000	-53.03680	73.39743	ANIC (Australia)	MT701236	MT701416	MT701324	MT701048	MT701142
		Bbr_HD04	Heard (HIMI)	2000	-53.02712	73.37838	ANIC (Australia)	MT701237	MT701417	MT701325	MT701049	MT701143
	*cf. brevis**	Bcfbr_CR02	Possession (Crozet)	2017	-46.37928	51.80333	ANIC (Australia)	MT701244	MT701425	MT701333	MT701057	MT701151
		Bcfbr_CR03	Possession (Crozet)	2016	-46.35243	51.72424	INRA (France)	MT701245	MT701426	MT701334	MT701058	MT701152
		Bcfbr_CR04	Possession (Crozet)	2013	-46.45963	51.83105	INRA (France)	MT701246	MT701427	MT701335	MT701059	MT701153
	crozetensis	Bcr_CR01	Possession (Crozet)	2016	-46.39308	51.81997	ANIC (Australia)	MT701241	MT701421	MT701329	MT701053	MT701147
		Bcr_CR02	Possession (Crozet)	2016	-46.43169	51.80248	ANIC (Australia)	MT701242	MT701422	MT701330	MT701054	MT701148
		Bcr_CR03	Possession (Crozet)	2016	-46.35239	51.72629	INRA (France)	MT701243	MT701423	MT701331	MT701055	MT701149
	daviesi	Bda_CR01	Possession (Crozet)	2017	-46.43567	51.83423	ANIC (Australia)	-	MT701424	MT701332	MT701056	MT701150
	desolationis	Bde_CR01	Possession (Crozet)	2017	-46.43354	51.86074	Uni of Rennes (France)	-	MT701428	MT701336	MT701060	MT701154
	elongatus	Bel_MA01	Marion (PEI)	2016	-46.89094	37.79452	ANIC (Australia)	MT701247	MT701429	MT701337	MT701061	MT701155
		Bel_MA02	Marion (PEI)	2016	-46.89094	37.79452	ANIC (Australia)	MT701248	MT701430	MT701338	MT701062	MT701156
		Bel_MA03	Marion (PEI)	2016	-46.89094	37.79452	ANIC (Australia)	MT701249	MT701431	MT701339	MT701063	MT701157
		Bel_PE03	Prince Edward (PEI)	2010	-46.62671	37.93181	ANIC (Australia)	MT701250	MT701432	MT701340	MT701064	MT701158
		Bel_PE04	Prince Edward (PEI)	2010	-46.62671	37.93181	ANIC (Australia)	MT701251	MT701433	MT701341	MT701065	MT701159
		Bel_PE05	Prince Edward (PEI)	2010	-46.62671	37.93181	ANIC (Australia)	MT701252	MT701434	MT701342	MT701066	MT701160
	fasciatus	Bfa_CR01	Possession (Crozet)	2016	-46.39131	51.80719	ANIC (Australia)	-	MT701436	MT701343	MT701067	MT701161

	gracilipes	Bgr_KR01	Kerguelen (Kerguelen)	2017	-49.34260	70.06397	ANIC (Australia)	MT701256	MT701440	MT701347	MT701071	MT701165
		Bgr_KR04	Kerguelen (Kerguelen)	2017	-49.34260	70.06397	ANIC (Australia)	MT701257	MT701441	MT701348	MT701072	MT701166
		Bgr_KR06	Kerguelen (Kerguelen)	2017	-49.34260	70.06397	ANIC (Australia)	MT701258	MT701442	MT701349	MT701073	MT701167
		Bgr_HD01	Heard (HIMI)	2000	-53.01822	73.35160	ANIC (Australia)	MT701253	MT701437	MT701344	MT701068	MT701162
		Bgr_HD02	Heard (HIMI)	2000	-53.01822	73.35160	ANIC (Australia)	MT701254	MT701438	MT701345	MT701069	MT701163
		Bgr_HD03	Heard (HIMI)	2000	-53.01822	73.35160	ANIC (Australia)	MT701255	MT701439	MT701346	MT701070	MT701164
	gravis	Bgv_CR02	Possession (Crozet)	2017	-46.45299	51.78559	ANIC (Australia)	MT701259	MT701435	MT701350	MT701086	MT701168
	huntleyi	Bhu_MA01	Marion (PEI)	2016	-46.88489	37.86807	ANIC (Australia)	MT701260	MT701443	MT701351	MT701074	MT701169
		Bhu_MA02	Marion (PEI)	2016	-46.88459	37.86807	ANIC (Australia)	MT701261	MT701444	MT701352	MT701075	MT701170
		Bhu_MA09	Marion (PEI)	2016	-46.89094	37.79452	ANIC (Australia)	MT701262	MT701445	MT701353	MT701076	MT701171
		Bhu_PE01	Prince Edward (PEI)	2010	-46.63225	37.94998	ANIC (Australia)	MT701263	MT701446	MT701354	MT701077	MT701172
		Bhu_PE02	Prince Edward (PEI)	2010	-46.63225	37.94998	ANIC (Australia)	MT701264	MT701447	MT701355	MT701078	MT701173
		Bhu_PE03	Prince Edward (PEI)	2010	-46.63225	37.94998	ANIC (Australia)	MT701265	MT701448	MT701356	MT701079	MT701174
	parvulus	Bpa_MA02	Marion (PEI)	2010	-46.88454	37.86893	ANIC (Australia)	MT701266	MT701449	MT701357	MT701080	MT701175
		Bpa_MA03	Marion (PEI)	2010	-46.88454	37.86893	ANIC (Australia)	MT701267	MT701450	MT701358	MT701081	MT701176
		Bpa_MA07	Marion (PEI)	2010	-46.88454	37.86893	ANIC (Australia)	-	MT701451	MT701359	MT701082	MT701177
		Bpa_PE01	Prince Edward (PEI)	2010	-46.65996	37.93460	ANIC (Australia)	MT701268	MT701452	MT701360	MT701083	MT701178
		Bpa_PE02	Prince Edward (PEI)	2010	-46.65996	37.93460	ANIC (Australia)	MT701269	MT701453	MT701361	MT701084	MT701179
		Bpa_PE05	Prince Edward (PEI)	2010	-46.65996	37.93460	ANIC (Australia)	MT701270	MT701454	MT701362	MT701085	MT701180
	randi	Bra_CR01	Possession (Crozet)	2016	-46.45856	51.72686	INRA (France)	MT701271	MT701455	MT701363	MT701087	MT701181
		Bra_CR02	Possession (Crozet)	2016	-46.45856	51.72686	INRA (France)	MT701272	MT701456	MT701364	MT701088	MT701182
		Bra_MA03	Marion (PEI)	2016	-46.88459	37.86807	ANIC (Australia)	MT701273	MT701457	MT701365	MT701089	MT701183
		Bra_MA04	Marion (PEI)	2016	-46.88489	37.86807	ANIC (Australia)	MT701274	MT701458	MT701366	MT701090	MT701184
		Bra_MA05	Marion (PEI)	2016	-46.88489	37.86807	ANIC (Australia)	MT701275	MT701459	MT701367	MT701091	MT701185
	comes	**Unsampled**										
	sulcatus	**Unsampled**										
	dreuxi	**Unsampled**										
	variabilis	**Unsampled**										
	villiersi	**Unsampled**										
Canonopsis	*sericea*^	Cse_HD05	Heard (HIMI)	2000	-53.01883	73.35870	ANIC (Australia)	MT701280	MT701466	MT701374	MT701098	MT701192

		Cse_HD07	Heard (HIMI)	2000	-53.02712	73.37838	ANIC (Australia)	MT701281	-	MT701375	MT701099	MT701193
		Cse_HD08	Heard (HIMI)	2000	-53.02712	73.37838	ANIC (Australia)	MT701282	-	MT701376	MT701100	MT701194
Christensenia	*antarctica*	Can_CR01	Possession (Crozet)	2015	-46.44474	51.80434	ANIC (Australia)	-	MT701460	MT701368	MT701092	MT701186
		Can_CR03	Possession (Crozet)	2016	-46.40875	51.78272	ANIC (Australia)	-	MT701461	MT701369	MT701093	MT701187
		Can_CR04	Possession (Crozet)	2016	-46.44474	51.80434	ANIC (Australia)	MT701276	MT701462	MT701370	MT701094	MT701188
	dreuxi	Cdr_CR03	Possession (Crozet)	2016	-46.43537	51.71147	ANIC (Australia)	MT701277	MT701463	MT701371	MT701095	MT701189
		Cdr_CR04	Possession (Crozet)	2016	-46.43610	51.74528	ANIC (Australia)	MT701278	MT701464	MT701372	MT701096	MT701190
		Cdr_CR05	Possession (Crozet)	2015	-46.43530	51.74703	INRA (France)	MT701279	MT701465	MT701373	MT701097	MT701191
Disker	*tenuicornis*	Dte_KR01	Kerguelen (Kerguelen)	2018	-49.55185	69.78029	ANIC (Australia)	MT701283	MT701467	MT701377	MT701101	MT701195
		Dte_KR02	Kerguelen (Kerguelen)	2018	-49.55185	69.78029	ANIC (Australia)	MT701284	MT701468	MT701378	MT701102	MT701196
Ectemnorhinus	*drygalskii*	Edr_KR01	Kerguelen (Kerguelen)	2014	-49.37948	70.44219	ANIC (Australia)	MT701285	MT701469	MT701379	MT701103	MT701197
	geniculatus	Ege_CR01	Possession (Crozet)	2017	-46.35648	51.76759	ANIC (Australia)	MT701286	MT701470	MT701380	MT701104	MT701198
	possessionensis	Epo_CR02	Possession (Crozet)	2017	-46.36771	51.74285	ANIC (Australia)	MT701288	MT701472	MT701381	MT701106	MT701200
		Epo_CR03	Possession (Crozet)	2015	-46.35476	51.72313	INRA (France)	MT701289	MT701473	MT701382	MT701107	MT701201
	richtersi	Eri_CR01	Possession (Crozet)	2013	-46.43169	51.85990	INRA (France)	MT701290	MT701474	MT701383	MT701108	MT701202
		Eri_CR02	Possession (Crozet)	2013	-46.43244	51.85970	INRA (France)	MT701291	MT701475	MT701384	MT701109	MT701203
		Eri_CR03	Possession (Crozet)	2013	-46.43354	51.85856	INRA (France)	MT701292	MT701476	MT701385	MT701110	MT701204
	similis	Esi_MA04	Marion (PEI)	2016	-46.89094	37.79452	ANIC (Australia)	MT701293	MT701477	MT701386	MT701111	MT701205
		Esi_MA08	Marion (PEI)	2016	-46.88469	37.86794	ANIC (Australia)	MT701294	MT701478	MT701387	MT701112	MT701206
		Esi_MA11	Marion (PEI)	2016	-46.88945	37.79650	ANIC (Australia)	MT701295	MT701479	MT701388	MT701113	MT701207
		Esi_PE01	Prince Edward (PEI)	2010	-46.64640	37.96475	ANIC (Australia)	MT701296	MT701480	MT701389	MT701114	MT701208
		Esi_PE02	Prince Edward (PEI)	2010	-46.63440	37.94521	ANIC (Australia)	MT701297	MT701481	MT701390	MT701115	MT701209
		Esi_PE04	Prince Edward (PEI)	2010	-46.63207	37.94225	ANIC (Australia)	MT701298	MT701482	MT701391	MT701116	MT701210
	vanhoeffenianus	Eva_CR02	Possession (Crozet)	2017	-46.43422	51.84029	ANIC (Australia)	MT701299	MT701483	MT701392	MT701117	MT701211
		Eva_CR04	Possession (Crozet)	2017	-46.43142	51.85891	ANIC (Australia)	MT701300	MT701484	MT701393	MT701118	MT701212
		Eva_CR05	Possession (Crozet)	2017	-46.43579	51.83423	ANIC (Australia)	MT701301	MT701485	MT701394	MT701119	MT701213
		Eva_CR06	Possession (Crozet)	2017	-46.43579	51.83423	ANIC (Australia)	MT701287	MT701471	MT701395	MT701105	MT701199
	viridis	Evi_KR01	Kerguelen (Kerguelen)	2013	-49.50920	70.05518	INRA (France)	MT701305	MT701489	MT701399	MT701123	MT701217
		Evi_KR02	Kerguelen (Kerguelen)	2013	-49.50706	70.03787	INRA (France)	MT701306	MT701490	MT701400	MT701124	MT701218

		Evi_HD01	Heard (HIMI)	2000	-53.01908	73.39239	ANIC (Australia)	MT701302	MT701486	MT701396	MT701120	MT701214
		Evi_HD03	Heard (HIMI)	2000	-53.01908	73.39239	ANIC (Australia)	MT701303	MT701487	MT701397	MT701121	MT701215
		Evi_HD04	Heard (HIMI)	2000	-53.01908	73.39239	ANIC (Australia)	MT701304	MT701488	MT701398	MT701122	MT701216
	kuscheli	**Unsampled**										
	bougainvillei	**Unsampled**										
	fuscus	**Unsampled**										
	inexpectatus	**Unsampled**										
	affinis	**Unsampled**										
	tamarisi	**Unsampled**										
Palirhoeus	*eatoni*	Pea_CR03	Possession (Crozet)	2016	-46.39308	51.81997	ANIC (Australia)	MT701307	MT701491	MT701401	MT701125	MT701219
		Pea_CR45	Possession (Crozet)	2016	-46.39308	51.81997	ANIC (Australia)	MT701308	MT701492	MT701402	MT701126	MT701220
		Pea_HD01	Heard (HIMI)	2000	-53.02712	73.37838	ANIC (Australia)	MT701309	MT701493	MT701403	MT701127	MT701221
		Pea_HD02	Heard (HIMI)	2000	-53.02712	73.37838	ANIC (Australia)	MT701310	-	MT701404	MT701128	MT701222
		Pea_HD19	Heard (HIMI)	2000	-53.02712	73.37838	ANIC (Australia)	MT701311	MT701494	MT701405	MT701129	MT701223
		Pea_KR11	Kerguelen (Kerguelen)	2017	-49.35410	70.07931	ANIC (Australia)	MT701312	MT701495	-	MT701130	MT701224
		Pea_KR13	Kerguelen (Kerguelen)	2017	-49.35410	70.07931	ANIC (Australia)	MT701313	MT701496	MT701406	MT701131	MT701225
		Pea_KR21	Kerguelen (Kerguelen)	2017	-49.35410	70.07931	ANIC (Australia)	MT701314	MT701497	-	MT701132	MT701226
		Pea_MA17	Marion (PEI)	2016	-46.88489	37.86807	ANIC (Australia)	MT701315	MT701498	MT701407	MT701133	MT701227
		Pea_MA18	Marion (PEI)	2016	-46.88489	37.86807	ANIC (Australia)	MT701316	MT701499	MT701408	MT701134	MT701228
		Pea_MA25	Marion (PEI)	2016	-46.88489	37.86807	ANIC (Australia)	MT701317	MT701500	MT701409	MT701135	MT701229
		Pea_PE02	Prince Edward (PEI)	2010	-46.65996	37.93460	ANIC (Australia)	MT701318	MT701501	MT701410	MT701136	MT701230
		Pea_PE32	Prince Edward (PEI)	2010	-46.64546	37.99778	ANIC (Australia)	MT701319	MT701502	MT701411	MT701137	MT701231
		Pea_PE33	Prince Edward (PEI)	2010	-46.65996	37.93460	ANIC (Australia)	MT701320	MT701503	MT701412	MT701138	MT701232
Pachnobium	*dreuxi*		**Unsampled:** originally described from fossil, only two modern specimens exist worldwide (both damaged); placement in Ectemnorhinini debated									

† ANIC: Australian National Insect Collection, Clunies Ross street, Acton, Australian Capital Territory (ACT) 2601, Australia.
INRA: Institut National de Recherche pour l'Agriculture, l'Alimentation et l'Environnement, Rue de Saint-Brieuc Batiment 13, 35000 Rennes, France.
University of Rennes: Ecosystèmes, Biodiversité, Evolution (ECOBIO), Campus Beaulieu, 35042 Rennes, France.

* These three specimens were identified as '*Bothrometopus cf. brevis*', as on collection they were deemed morphologically identical to *B. brevis*; however *B. brevis* itself has not officially been recorded from the Crozet Islands previously.

^ *Canonopsis sericea* is also found on Kerguelen Island, from which we were unable to obtain specimens

Table S14 Collection details for *Palirhoeus eatoni* populations used for phylogeographic analysis. The sample size shown (*N*) is that which remained after excluding individuals with >50% missing SNP data (i.e. the final sample size analyzed). The dataset of genome-wide SNPs for all populations has been archived on Figshare at doi:10.26180/14446023.

Archipelago	Island	Site	*N*	Latitude	Longitude	Collection dates
HIMI	Heard					
		HD	28	-53.02711	73.37839	Dec 2000 – Mar 2001
Kerguelen	Kerguelen					
		KR-a	9	-49.38148	70.44394	Mar 2014
		KR-b	29	-49.35410	70.07931	Jan 2017
		KR-c	10	-49.21606	69.86580	Mar 2016
Crozet	Possession					
		PO-a	36	-46.39308	51.81997	Dec 2016
		PO-b	9	-46.36005	51.71759	Nov 2016
		PO-c	10	-46.38185	51.66161	May 2015
PEI	Prince Edward					
		PE-a	23	-46.64546	37.99778	Apr 2010
		PE-b	20	-46.65996	37.93460	Apr 2010
	Marion					
		MA	51	-46.88489	37.86893	Apr 2010 & Dec 2016

Text S4: Phylogenomic inference and molecular dating: detailed methods

PartitionFinder v.1.1.1 (20) was used to find the best-fit cluster of partitions for the 'C12' dataset of 1st and 2nd codons for the supermatrix of 515 genes (deposited on the Zenodo Digital Repository at doi:10.5281/zenodo.3955188). We used the corrected Akaike information criterion (AICc) for model selection, 'linked' branch lengths, an 'rcluster' search algorithm and the models GTR+G+I and GTR+G. Based on PartitionFinder outcomes (partitioning scheme archived at doi:10.5281/zenodo.3955188), the dataset was partitioned and analysed under the GTR+G+I model using Maximum Likelihood (ML) phylogenetic inference conducted in RAxML v.8 (21). We completed 10 separate thorough ML tree searches and 100 slow bootstrap replicates using the University of Memphis HPC cluster on nodes with 48 cores and 1TB RAM.

To estimate divergence times, the PAML package (containing MCMCtree and codeml) (22) was employed to generate a timetree using a Markov chain Monte Carlo (MCMC) approach. We used the data from 1st and 2nd codon positions and the partitioned (best) ML tree as input for our analysis. The root constraint was applied as a maximum age of 165 Ma, which is the age of the oldest weevil fossils known (23). We applied a further seven fossil calibrations (see Text S5) as soft minimum ages (truncated Cauchy distributions), using the following parameters for each calibration point: offset 0.1, scale parameter 1 and left tail probability 0.025. An independent-rates model was used to relax the clock, with default parameter settings. The analysis was run for 2 million generations, sampling every 10 iterations and applying a 1% burn-in. Four separate MCMCtree runs were implemented on the University of Memphis HPC cluster. We determined the resulting effective sample size (ESS) using the program Tracer v.1.7.1 (24), ensuring that the ESS for all parameters exceeded 200, as recommended in the MCMCtree manual. The resulting output files were checked for convergence using a custom plotting script in R (25) and all four runs were determined to have converged.

Text S5: Fossil choice and placement for phylogenomic timetree calibration

We conducted a careful assessment of the fossils assigned in the literature to the lineages included in our taxon set. Although the fossil record for weevils is fairly extensive, the majority of fossils with reliable dates and plausible identifications fall outside the family Curculionidae, which is the focus here. Most fossils assigned to Curculionidae are generally poorly preserved and have rarely been re-appraised since original descriptions a century or more ago. Additionally, very few current subfamilies and tribes of Curculionidae are demonstrably monophyletic (identified by synapomorphic characters), making the assignment of extinct taxa often difficult. Thus, we were conservative in our selection approach, ultimately identifying seven fossils as sufficiently reliable and representative to be used as calibration points in our phylogenomic timetree.

A. The root of the timetree, being the divergence of the families Brentidae and Curculionidae, was dated with two fossils from the Crato Formation in Santana, Brazil, *Axelrodiellus ruptus* and *Arariperhinus monnei*, of Aptian age (125–112 Ma). The former taxon was tentatively assigned to the brentid subfamily Eurhynchinae (26) and the latter more definitely to the curculionid subfamily Curculioninae (27), although this subfamily placement is not unequivocal (28). However, the salient character of both fossils, the elongate basal two ventrites of the abdomen, indicates that they are indeed representatives of the families Brentidae and/or Curculionidae.

B. The brentid subfamily Eurhynchinae was dated with several compression fossils from the Orapa kimberlite diatreme in Botswana described as *Orapaeus cretaceus* (29), of Turonian age (91 Ma) (30, 31). The assignment of the taxon to Eurhynchinae is based on its elongate ventrites 1 and 2, loose antennal clubs and indicated protibial notch (as occurs only in Eurhynchinae).

C. The root of the subfamilies Hyperinae, Cyclominae and Entiminae was dated with a fossil from the Dorotea Formation in Chile described as *Dorotheus guidensis* (32), of Maastrichtian age (70–65 Ma). The single elytron was tentatively assigned to the tribe Cylydrorhinini (Entiminae) (32), but at the time the concept of Cylydrorhinini also included the current tribe Listroderini (Cyclominae) and the 10 complete striae of the elytron actually accord with Listroderini rather than Cylydrorhinini. However, as 10 complete striae also occur in the subfamily Hyperinae (e.g. the South American genus *Phelypera*) and the apparent forest environment of the stratum contradicts an assignment of the fossil to Listroderini (32), it is more likely to represent the subfamily Hyperinae and we therefore used it to date the root of the Hyperinae-Entiminae-Cyclominae clade.

D. The stem of the tribe Tanymecini (apparently including Cratopodini) was dated with a fossil in Fushun amber described as *Hypomeces fushunensis* (33), of Eocene age (56–34 Ma). Based on the color photograph, drawings and detailed comparison with *Hypomeces* "*squamosus*" in the original description, the specimen evidently represents either *Hypomeces* or a similar tanymecine genus and was therefore taken here to represent the Tanymecini lineage.

E. The Palaearctic tribe Otiorhynchini was dated with a fossil from the Messelgrube in Germany described as *Palaeoalatorostrum schaali* (34), of Lutetian age (48–41 Ma). The specimen was compared with the genus *Otiorhynchus* and provisionally placed in Otiorhynchini, a plausible assignment judging from the illustrations provided.

F. The tribe Cneorhinini was dated with a fossil from the Messelgrube in Germany described as *Palaeocneorhinus messelensis* (34), of Lutetian age (48–41 Ma). The fossil was compared with the genera *Cneorhinus* and *Attactagenus* and hence placed in Cneorhinini, again a plausible assignment based on the illustrations and characters provided. We used this fossil with some hesitation as the tribe Cneorhinini is not directly represented in our analysis and the two African genera currently placed in this tribe (*Philopedon* and *Protostrophus*) appear in different clades in our tree, the former with low support as adelphic to *Sciomias* (Sciaphilini) and the latter with strong support in a clade with *Blosyrus* and *Proscephaladeres* (both classified as Blosyrini). As *Cneorhinus* is very likely to belong in the same larger clade (including also Brachyderini), we calibrated the Blosyrini clade with the age of *Palaeocneorhinus.*

G. The tribe Geonemini (including Eustylini) was dated with several fossils from Dominican amber, of Burdigalian age (20–15 Ma), e.g. *Diaprepes anticus, Scelionoma compacta* and *Tropirhinus palpebratus* (35) and *Diaprepes squamula* and *Lachnopus serraticrus* (36). The current tribes Geonemini and Eustylini form a single clade in the phylogenetic analysis of ref. 37, as do our two representative genera, *Epicaerus* and *Exophthalmus*, and the two tribes evidently represent a single lineage, which we therefore calibrated with the age of Dominican amber.

x. Supplementary methods for phylogenetics

Table S15 Details of primers and thermal cycling protocol for the PCR amplification of three mitochondrial and two nuclear genes in the Ectemnorhinini.

Genome	Gene	Annealing protocol	Forward primer	Reverse primer	Primer reference
mitochondrial	COI	4 cycles at 47°C,<br>35 cycles at 53-54°C	ATTCAACCAATCATAAAGATATTGG	TAAACTTCTGGATGTCCAAAAAATCA	(38)
mitochondrial	CYB	35 cycles at 53°C	GAGGAGCAACTGTAATTACTAA	AAAAGAARTATCATTCAGGTTGAAT	(39)
mitochondrial	16S	34 cycles at 52°C	GGTCCTTTCGTACTAA	CRCCTGTTTATTAAAAACAT	(40)
nuclear	28S	40 cycles at 55-57°C	GACTACCCCCTGAATTTAAGCAT	GACTCCTTGGTCCGTGTTTCAAG	(41)
nuclear	EF1a	40 cycles at 55°C	CTGGTGAATTTGAAGCYGGTA	CCACCAATTTTGTAGACATC	*Forward*: (42)<br>*Reverse*: (43)

Table S16 Results from Xia's substitution saturation test conducted in DAMBE 7.0 (44) for the third codon of the COI gene, across the entire Ectemnorhinini phylogeny. Xia's test calculates a saturation index ('Iss') and assesses whether this is significantly less than the critical index ('Iss.c') beyond which sequences fail to recover the true tree (depending on the number of MOTUs and topological symmetry of the true tree: see ref. 45). The true topology contains between 16 and 32 MOTUs and has elements of both symmetrical and asymmetrical topology (highlighted results). Under an asymmetrical topology, saturation was found to be significant (Iss ≮ Iss.c; results in bold typeface). Saturation tests for all other genes and codons did not detect significant saturation.

		Symmetrical			Asymmetrical		
Number MOTUs	Iss	Iss.c	Iss < Iss.c?		Iss.c	Iss < Iss.c?	
			T statistic	p-value		T statistic	p-value
4	0.439	0.777	9.975	<0.001	0.761	9.491	<0.001
8	0.456	0.732	7.756	<0.001	0.629	4.877	<0.001
16	0.468	0.657	5.211	<0.001	**0.460**	**0.229**	**0.819**
32	0.477	0.684	5.499	<0.001	**0.361**	**3.080**	**0.002**

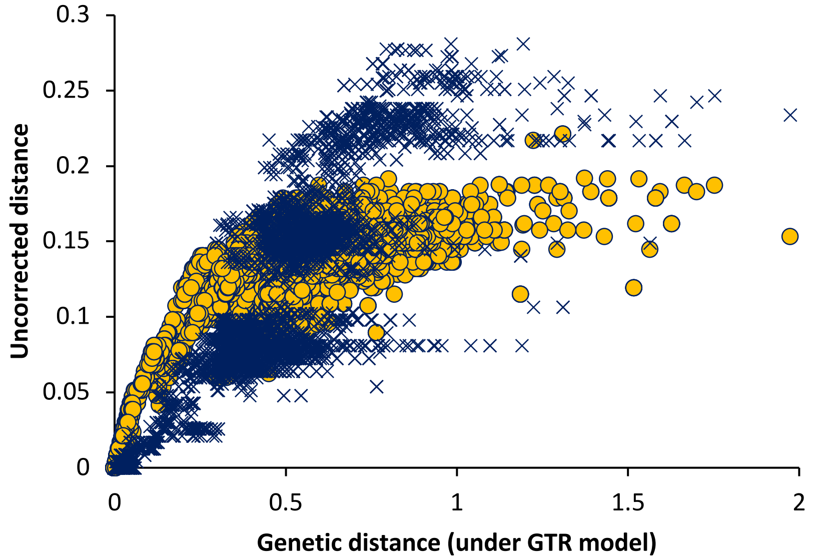

Figure S15 Substitution saturation plot for COI 3rd codon across entire Ectemnorhinini phylogeny. Blue crosses represent transversions, gold circles represent transitions. The slope begins to plateau as genetic distance increases, indicating significant saturation (particularly for transitions).

Text S6: Phylogenetic inference and molecular dating: detailed methods

Relationships among Ectemnorhinini species were initially explored using Bayesian inference (BI) and Maximum Likelihood (ML) analyses of a concatenated dataset of the three mitochondrial and two nuclear genes. BI analysis was carried out in MrBayes v.3.2.6 on the CIPRES Science Gateway 3.3 (46). Best-fitting substitution models were applied independently to partitions of the sequence data (see Table S17), with partitions and models selected in PartitionFinder using the 'greedy' search algorithm, 'mrbayes' set of models, 'linked' branch lengths and AICc. Two independent BI analyses using four MCMC chains (three heated, one cold) were run for 20 million generations from random starting trees, sampled every 10,000 generations. Mixing and convergence of analyses were assessed using Tracer. Burn-in was conservatively set to 20%, with remaining trees used to build a majority-rule consensus tree. ML analysis was conducted with RAxML on the CIPRES Gateway, using the GTR+G substitution model applied independently to data partitions as determined by PartitionFinder (Table S17). Rates were allowed to vary across partitions and the 'autoMRE' algorithm determined a sufficient number of bootstrap replicates. BL and MI phylogeny estimates were compared to determine congruence – with one another, with the phylogenomic tree generated by AHE and with morphological species designations. As some phylogenetic clades were inconsistent with morphological species designations (which have long been contested anyway), ML and BI trees were used to designate molecular operational taxonomic units (MOTUs) for subsequent analyses, as detailed in this appendix, section ii.

Divergence dates among Ectemnorhinini were estimated from our five-gene dataset by linking gene trees, as implemented in BEAST v.2.5 (47), and by co-estimating gene trees embedded in a shared species tree, as implemented in starBEAST (48). For analysis in BEAST we randomly selected one sequence to represent each MOTU, as BEAST assumes either a coalescent or phylogenetic divergence process and a mixture of both is misleading. We also performed a second analysis with one sequence selected to represent each traditional morphological species, to ensure our use of MOTUs did not affect subsequent inferences. The partitioning strategy identified by PartitionFinder was employed (Table S17), with the best substitution model for each partition selected using Bayesian model averaging implemented in bModeltest (49). Uncorrelated relaxed lognormal clocks allowed for rate variation among lineages. Given the geo-glacial history of the sub-Antarctic, we considered extinction highly likely, thus a birth-death process was used for the tree prior. For nodes previously found to be incongruent between the BI/ML phylogenetic trees and the AHE-derived phylogenomic tree, we enforced the topology of the phylogenomic tree (see Figure S17), as it had higher support and was consistently more congruent with morphological hypotheses (e.g. *Palirhoeus* as basal to *Bothrometopus*: ref. 2). For calibration, geological (island formation) constraints were applied as uniform upper bounds and secondary fossil-derived constraints were applied as normal priors (detailed in Text S7). A second analysis was run with secondary constraints excluded but substitution rates calculated previously for Coleoptera provided as lognormal priors for the mean rates of relaxed clocks (see Text S7).

In contrast to BEAST, starBEAST performs optimally with multiple samples per lineage, thus we retained the entire dataset of sequences for these analyses. All other parameters were the same as for the BEAST analyses, except that individual genes formed the partitions (with only the mitochondrial gene trees linked) and we used Analytical Population Size Integration as the population model. MCMC analyses in both BEAST and starBEAST were run for 200 million generations, sampling one in every 10,000 trees. Outputs were checked in Tracer to ensure that stationarity had been reached and that the ESS of all parameters exceeded 200. The first 20% of trees were discarded as burn-in and the remainder were used to generate dated maximum clade credibility trees.

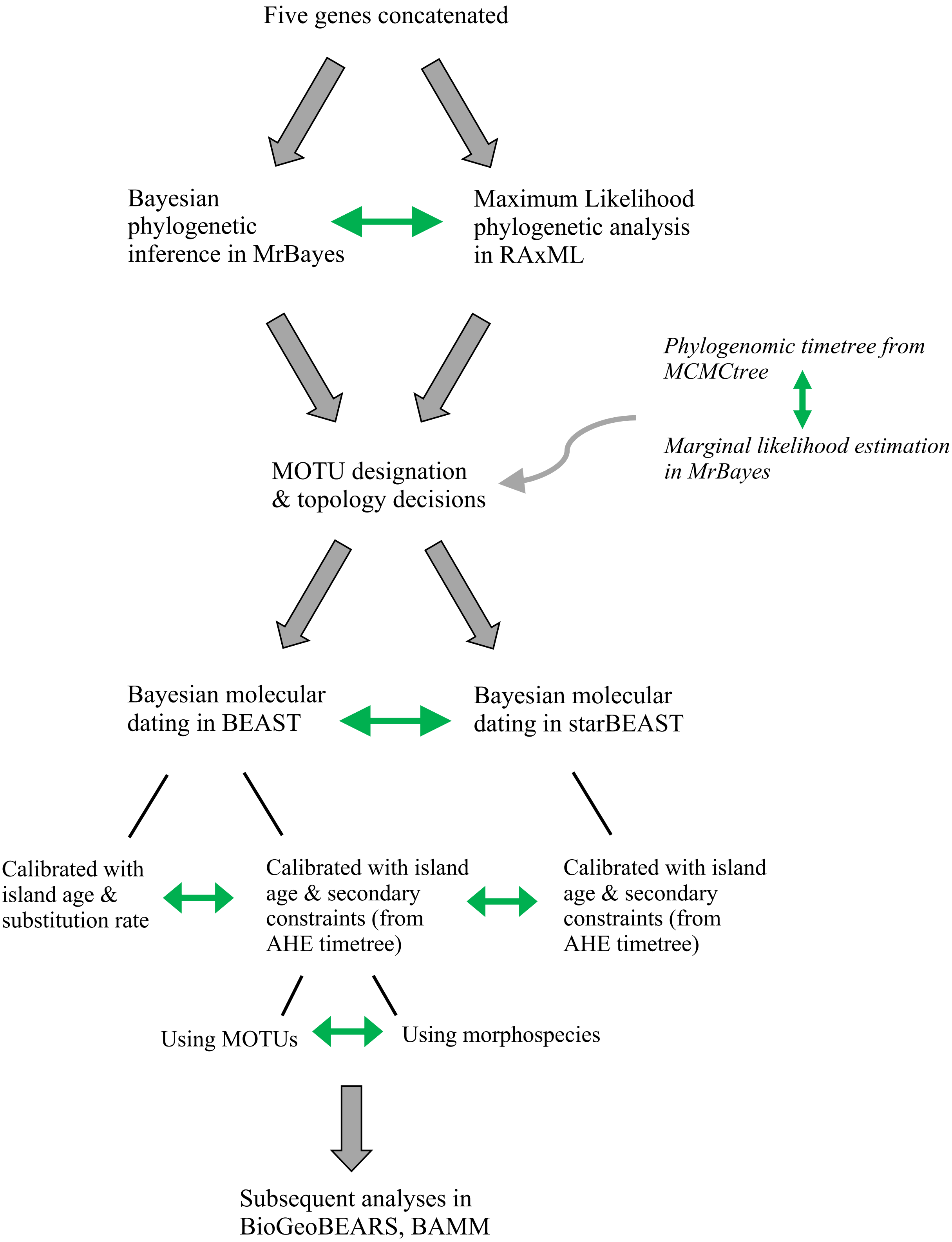

Figure S16 Overview of phylogenetic and biogeographic inference analyses conducted on the concatenated 5-gene dataset for Ectemnorhinini (with input from phylogenomics in italics). Green arrows indicate cross-checks for congruence among analyses.

Table S17 Best-fit partitioning strategies and substitution models for the concatenated 5-gene dataset for Ectemnorhinini, identified by corrected Akaike information criterion (AICc) in PartitionFinder v.1.1.1 (20). For MrBayes analyses, the full set of models available were tested, whereas for RaxML analyses all partitions were necessarily assigned the same model (GTR+G). Model testing was performed on both the original concatenated dataset and the dataset with COI 3rd codons RY-coded for saturation.

cod = codon

	Original sequences		*COI 3rd codon RY-coded*	
**RAxML**	16S	GTR+G	16S	GTR+G
	EF1a-cod1, 28S	GTR+G	EF1a-cod1, 28S	GTR+G
	CYB-cod1, COI-cod1	GTR+G	CYB-cod1, COI-cod1	GTR+G
	EF1a-cod2, COI-cod2, CYB-cod2	GTR+G	EF1a-cod2, COI-cod2, CYB-cod2	GTR+G
	COI-cod3	GTR+G	COI-cod3	GTR+G
	CYB-cod3	GTR+G	CYB-cod3	GTR+G
	EF1a-cod3	GTR+G	EF1a-cod3	GTR+G
**MrBayes**	16S	GTR+I+G	16S	GTR+I+G
	EF1a-cod1, 28S	GTR+I+G	EF1a-cod1, 28S	GTR+I+G
	CYB-cod1, COI-cod1	GTR+I+G	CYB-cod1, COI-cod1	GTR+I+G
	EF1a-cod2, COI-cod2, CYB-cod2	GTR+I+G	EF1a-cod2, COI-cod2, CYB-cod2	GTR+I+G
	COI-cod3	GTR+I+G	COI-cod3	SYM+G
	CYB-cod3	GTR+I+G	CYB-cod3	GTR+I+G
	EF1a-cod3	HKY+I	EF1a-cod3	HKY+I

Text S7: Prior settings and time calibrations used in BEAST and starBEAST analyses

Molecular dating of divergences among the Ectemnorhinini was carried out in both BEAST and starBEAST. Whereas the datasets and partitions used in these two programs differed (see main text), the parameters and priors outlined below were implemented in both.

Tree prior

A birth-death model was used as the tree prior to allow for extinction, with birth rate and death rate given default priors.

Topology constraints

The genera *Bothrometous* and *Palirhoeus* were constrained as monophyletic, and the genera *Disker, Ectemnorhinus, Bothrometopus and Palirhoeus* were constrained as monophyletic (see Figure S17), reflecting outcomes from the Anchored Hybrid Enrichment (AHE) phylogenomic timetree, as well as long-standing hypotheses based on morphology (2).

Geological constraints

The geological age of the PEI archipelago was used as a uniform hard upper-bound age constraint for divergences within morphospecies for which one clade exclusively inhabits the PEI (and therefore could not have diverged earlier than the islands' subaerial existence). Deeper, morphologically recognized species divergences with PEI-inhabiting taxa were not constrained in this way, because extinct or unsampled species could confound the age of such nodes (50). In our supplementary analyses using morphospecies (rather than MOTUs) for tree tips, only the divergence between the very closely related *B. crozetensis* and *B. parvulus* was constrained using this calibration.
The maximum age determination for rocks from Marion Island (the older of the two PEI islands) is reported as 454 +/- 21 ka using potassium-argon dating (51). We therefore conservatively used 475 ka as the maximum age estimate for these divergences (see Figure S17). Geological ages of the other archipelagos were not applied as constraints, because the submarine plateaus on which they sit have undergone substantial uplift and subduction, thus other parts of these archipelagos may have been subaerial even earlier (see ref. 52).

Secondary fossil-derived constraints

Two key nodes (see Figure S17) were constrained using age estimates taken from the phylogenomic timetree, which we considered robust given its 515 genes and 7 discrete fossil calibrations. These priors were applied as normal distributions with standard deviations set to capture the 95% highest posterior density (HPD) interval corresponding to the AHE estimate (as recommended in ref. 53). Thus, the crown was given a normal prior with mean = 47.1 Ma and sigma = 5 (95% HPD = 37.3–56.9 Ma), and the divergence of *Ectemnorhinus* from *Bothrometopus-Palirhoeus* was given a normal prior with mean = 22.45 Ma and sigma = 3.35 (95% HPD = 15.9–29.0 Ma). These calibrations were only used in the analyses where clock rates were given non-informative priors (see below).

Clock model and priors

Uncorrelated relaxed lognormal clocks were applied to each partition, allowing for rate variation among lineages according to a lognormal distribution. The standard deviation of this distribution was itself exponential, with a mean of 0.3. The mean rate for each relaxed clock ('ucldMean' in BEAST; 'uclnClockRate' in starBEAST) was given a lognormal prior,

with a mean that was either informative (based on rates provided in the Coleoptera literature) or non-informative, as detailed below.

Geological and secondary fossil-derived calibrations applied

Geological and informative rate calibrations applied, no secondary constraints

	Non-informative rates		Informative rates	
BEAST	ucldMean	Justification	ucldMean	Justification
16S	0.001		0.0054	Rate deduced for 16S in ref. 54
28S, EF1a-cod1	0.0001		0.0006	Rate deduced for 28S in ref. 54
CYB-cod1, COI-cod1	0.001	Indication of order of magnitude, as per BEAST manual	0.00175	Rate deduced for 1st codon of mitochondrial PCGs in ref. 55
EF1a-cod2, COI-cod2, CYB-cod2	0.001		0.00085	Rate deduced for 2nd codon of mitochondrial PCGs in ref. 55
COI-cod3 & CYB-cod3 (linked for clock model, not site model)	0.01		0.0242	Rate deduced for 3rd codon of mitochondrial PCGs in ref. 55
EF1a-cod3	0.01		0.01	No robust estimate of nuclear PCG 3rd codon rate, therefore order of magnitude provided
starBEAST	uclnClockRate	Justification		
16S	0.001			
28S	0.0001			
EF1a	0.001	As above		
CYB	0.01			
COI	0.01			

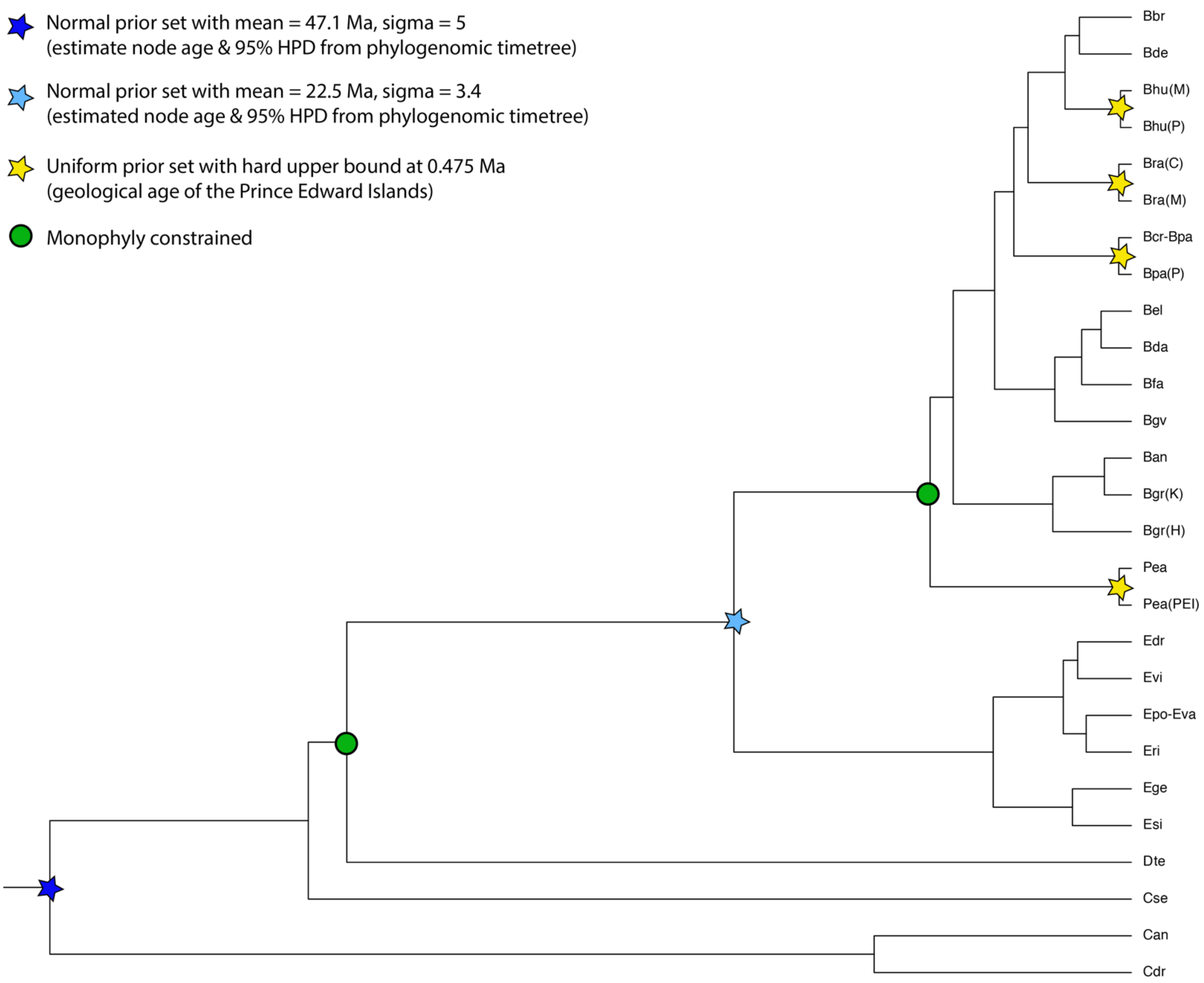

Figure S17 Phylogeny estimate for Ectemnorhinini weevils showing the placement of time calibrations and topology constraints for BEAST and starBEAST analyses.

xi. Supplementary results based on morphological species rather than MOTUs

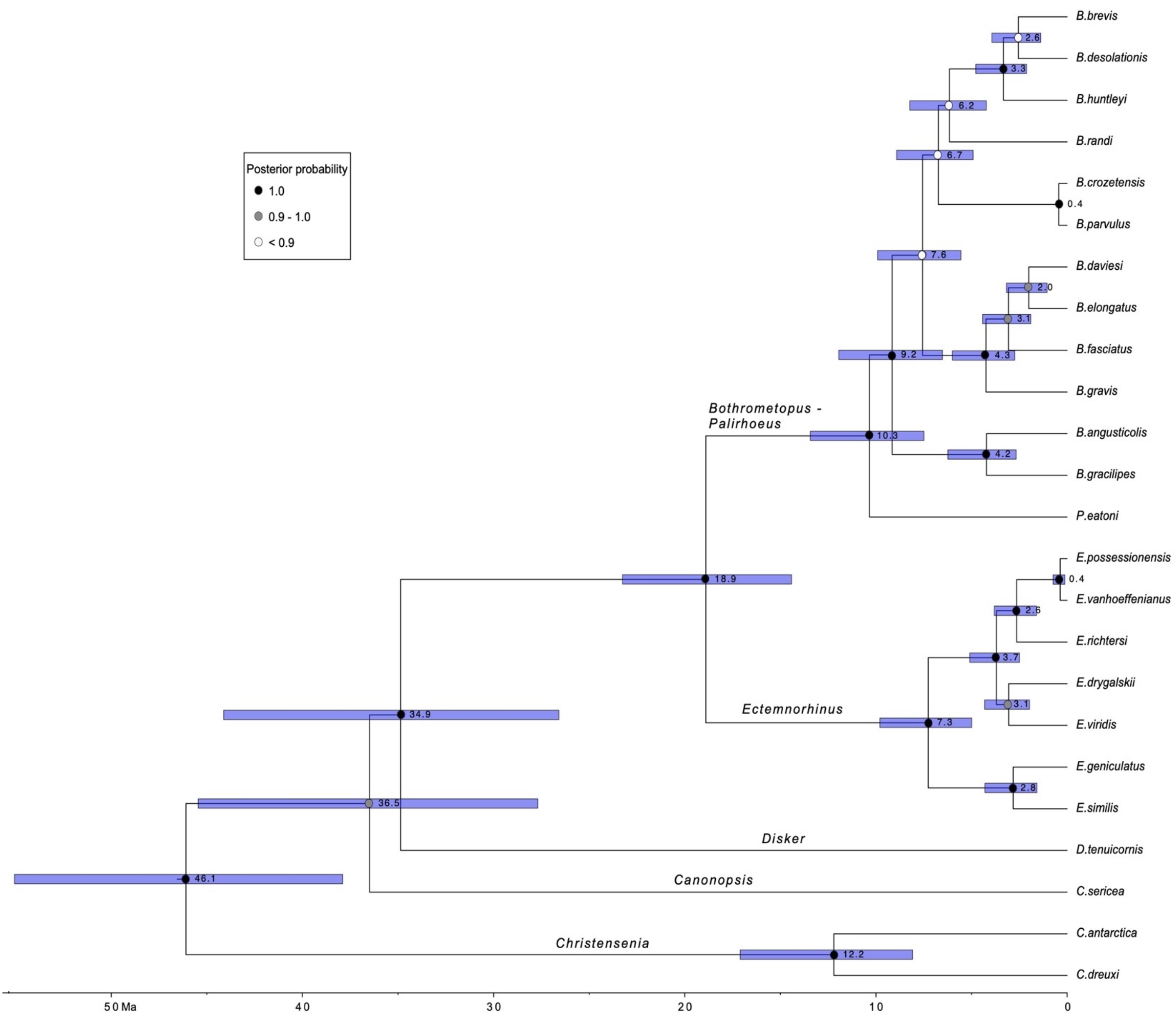

Figure S18 Maximum clade credibility tree for Ectemnorhinini generated in BEAST using data from three mitochondrial and two nuclear genes. The tree was calibrated with the geological emergence of the Prince Edward Islands archipelago and two key divergence dates estimated by our fossil-calibrated phylogenomic tree (see Text S7). Tips are labelled with morphological species; nodes are labelled with estimated node ages (in Ma). Error bars represent 95% highest posterior densities for estimated node age.

Table S18 Outcomes of historical biogeographic model inference in BioGeoBEARS using the DEC+*J*+*X* model, as well as the mean number of cladogenetic and anagenetic events inferred from 100 iterations of biogeographic stochastic mapping (BSM). The input phylogeny was that generated using morphological species at tree tips (rather than MOTUs). LnL = log-likelihood; *d* = dispersal parameter; *e* = extinction parameter; *J* = founder effect parameter; *X* = dispersal distance modifier

**Model outcomes**						**Mean event counts (from BSM)**				
						Anagenetic	Cladogenetic			
Model	LnL	*d*	*e*	*J*	*X*	Range expansion	Founder event	Within-archipelago sympatry	Subset sympatry	Vicariance
DEC+*J*+*X*	-68.88	0.36	0.03	0.42	-2.48	21	3	16	3	2
						Total dispersal events				
						24				

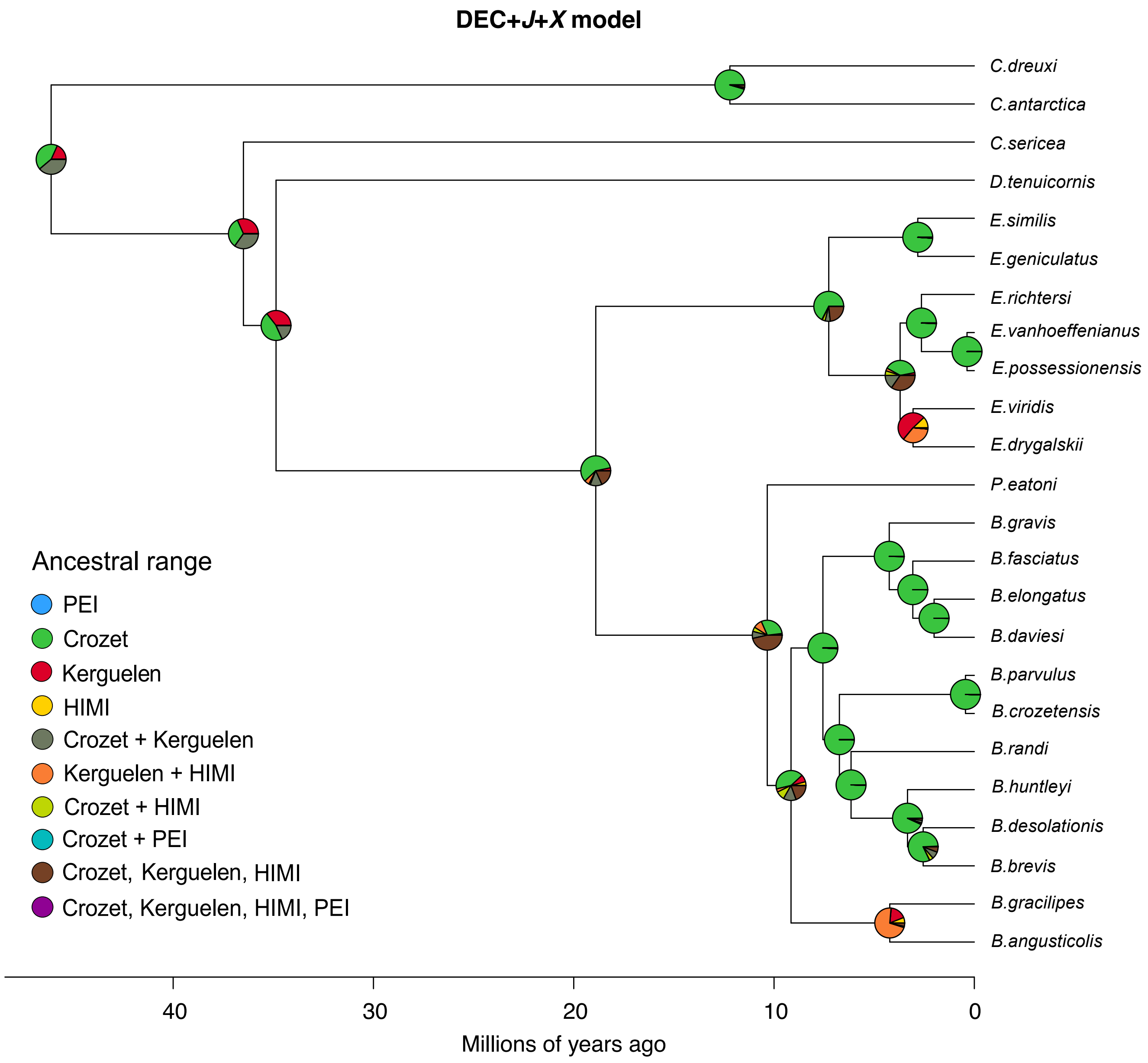

Figure S19 Ancestral range estimation based on the DEC+*J*+*X* biogeographic model implemented in BioGeoBEARS. Pie charts show the probability of ancestral ranges for each ancestral node throughout the phylogeny. Phylogeny tips represent morphological species.

Table S19 Mean number of total inter-archipelago dispersal events (both anagenetic and cladogenetic) inferred throughout the Ectemnorhinini evolutionary history based on the DEC+*J*+*X* model and 100 iterations of biogeographic stochastic mapping, using a phylogeny with morphological species at tree tips rather than MOTUs. Dispersal events inferred in a westerly direction are highlighted in yellow.
HIMI = Heard Island and McDonald Islands; K = Kerguelen Islands; C = Crozet Islands; PEI = Prince Edward Islands

		Dispersal into archipelago				
		HIMI	K	C	PEI	*Mean total dispersals out of archipelago:*
Dispersal from archipelago	HIMI	0	3.7	0.6	0.1	**4.4**
	K	6.3	0	2.1	0.2	**8.6**
	C	1.3	3.8	0	5.8	**10.9**
	PEI	0	0	0	0	**0**
Mean total dispersals into archipelago:		**7.6**	**7.5**	**2.7**	**6.1**	**Mean total dispersal events ≈ 24**

xii. Input data for historical biogeographic inference

Table S20 Distribution ranges for Ectemnorhinini MOTUs (and traditional morphological species in grey below), expressed as binary membership/non-membership to an archipelago, taken from ref. (19).
HIMI = Heard Island and McDonald Islands; K = Kerguelen Islands; C = Crozet Islands; PEI = Prince Edward Islands

MOTU / Morphospecies	Archipelago			
	HIMI	K	C	PEI
Ban	0	1	0	0
B. angusticolis	0	1	0	0
Bbr	1	1	1	0
B. brevis	1	1	1	0
Bcr-Bpa	0	0	1	1
Bpa(P)	0	0	0	1
B. crozetensis	0	0	1	0
B. parvulus	0	0	0	1
Bel	0	0	0	1
B. elongatus	0	0	0	1
Bfa	0	0	1	0
B. fasciatus	0	0	1	0
Bgr(K)	0	1	0	0
Bgr(H)	1	0	0	0
B. gracilipes	1	1	0	0
Bhu(M)	0	0	0	1
Bhu(P)	0	0	0	1
B. huntleyi	0	0	0	1
Bra(C)	0	0	1	0
Bra(M)	0	0	0	1
B. randi	0	0	1	1
Bde	0	0	1	0
B. desolationis	0	0	1	0
Bda	0	0	1	0
B. daviesi	0	0	1	0
Bgv	0	0	1	0
B. gravis	0	0	1	0
Can	0	0	1	0
C. antarcticus	0	0	1	0

Cdr	0	0	1	0
C. dreuxi	0	0	1	0
Edr	0	1	0	0
E. drygalskii	0	1	0	0
Epo-Eva	0	0	1	0
E. possessonensis	0	0	1	0
E. vanhoeffenianus	0	0	1	0
Esi	0	0	0	1
E. similis	0	0	0	1
Evi	1	1	0	0
E. viridis	1	1	0	0
Eri	0	0	1	0
E. richtersi	0	0	1	0
Cse	1	1	0	0
C. sericea	1	1	0	0
Dis	0	1	0	0
D. tenuicornis	0	1	0	0
Ege	0	0	1	0
E. geniculatus	0	0	1	0
Pea	1	1	1	0
Pea(PEI)	0	0	0	1
P. eatoni	1	1	1	1

Table S21 Matrix of geodesic distances (in kilometers) between archipelagos. Distances were calculated using the 'raster' package (56) in R and were measured between the centroid of archipelagos – themselves calculated as the centerpoint of landmasses using the 'rgeos' package (57) in R. All distances were rescaled prior to use in BioGeoBEARS by dividing by the smallest distance (such that this distance became 1).
HIMI = Heard Island and McDonald Islands; K = Kerguelen Islands; C = Crozet Islands; PEI = Prince Edward Islands

	HIMI	K	C	PEI
HIMI	0	507.16	1738.56	2625.55
K	507.16	0	1373.82	2360.78
C	1738.56	1373.82	0	1060.96
PEI	2625.55	2360.78	1060.96	0

xiii. Supplementary methods for phylogeography

Text S8: Genome-wide SNP library preparation and quality filtering

A genome-wide SNP library for *Palirhoeus eatoni* was developed using DArT-Seq™ Technology (58, 59) at Diversity Arrays Technology Pty. Ltd., Canberra, Australia. Preliminary testing selected the restriction enzyme combination *Pst*I–*Hpa*II to double-digest genomic DNA. This was followed by the ligation of adaptors to restriction enzyme overhangs (to facilitate sample identification and Illumina sequencing: see ref. 60). Fragments were PCR-amplified with an initial denaturation for 1 min at 94°C; 30 cycles each consisting of 20 s at 94°C, 30 s at 58°C, 45 s at 72°C; and final extension for 7 min at 72°C. Equimolar amounts of PCR product for each sample were pooled and applied to c-Bot (Illumina) bridge PCR followed by sequencing on Illumina Hiseq2500. Raw Illumina fastq files were processed with a proprietary DArT pipeline, wherein poor-quality reads were filtered at a minimum read Phred score of 10, with more stringent filtering of barcode regions (minimum Phred score of 30) to ensure sequences were reliably assigned to samples. Approximately 2,500,000 sequences per barcode/sample were identified and used in SNP calling. Fragments were trimmed to 69 bp, after which candidate SNP markers were called using a second DArT pipeline (DArTsoft14) and filtered for ≥ 20% call rate and a minimum read depth of 5 (average across all markers was > 40 reads per locus). Furthermore, a subset of technical replicates (processed from DNA through to SNP allelic calls) ensured that the dataset had an average of 99.5% scoring consistency.

The SNP dataset generated by DArT was further filtered using the 'dartR' package v.1.0.5 (61) in R. While less stringent call rates incur more missing data, they are likely to provide greater phylogenomic resolution due to retention of more markers (see ref. 62). We therefore filtered our SNPs to a moderate call rate of ≥ 80%. Where more than one SNP was called from the same fragment, these were randomly excluded until a single SNP remained per fragment, to minimize genetic linkage. The minimum minor allele frequency was set to 2% globally (across all geographic sites) or 20% locally (within a site). Finally, individuals with ≥ 50% missing data were excluded and any SNPs that became monomorphic as a result were also excluded. The number of SNPs remaining at each filtering step is shown in Table S22 and the final filtered SNP dataset is available on Figshare (doi:10.26180/14446023).

Table S22 Number of genome-wide SNPs retained for *Palirhoeus eatoni* at each stage of quality filtering. The final step (in grey) was only carried out for the conservative dataset. MAF = minor allele frequency; HWE = Hardy-Weinberg Equilibrium

Filtering step	SNPs remaining
Reproducibility > 90% + read depth ≥ 5	29,688
Call rate ≥ 80%	11,041
Secondary reads from the same fragment removed	7,514
MAF ≥ 2% globally or ≥ 20% locally	6,158
Monomorphic loci removed*	5,859
Loci violating HWE / putatively under selection removed	5,363

*Monomorphic loci were identified once individuals with ≥ 50% missing data had been removed from the dataset

Text S9: Phylogeographic analyses of SNP dataset: detailed methods

To assess and compare differences in genetic diversity among sites, observed and expected heterozygosity and inbreeding coefficients were calculated for each population of *Palirhoeus eatoni* using GenoDive v.2.0 (63), with 95% confidence intervals determined using 5,000 bootstraps.

Population structure was examined in a number of ways. Pairwise F_{ST} estimates among all populations were calculated in GenoDive, with 5,000 permutations to estimate significance. The clustering of individuals due to genetic similarity was visualized using Principal Coordinate Analysis (PCoA), conducted using the 'gl.pcoa' function in dartR. Scree plots were used to assess how many principal coordinates to examine; it was clear that the first two coordinates captured the majority of variation. A Bayesian clustering algorithm was also used to infer population structure, as implemented in fastSTRUCTURE (64). To determine the most appropriate number of populations to describe the data, we used the 'chooseK.py' function after running ten replicates for each K value from K=1 to K=15. Inferred population assignments for the range of best fitting K values were then visualized using Distruct v.2.3 (65). As it was clear that there were two distinct clusters of the data ('east' and 'west'), which swamped all finer-level genetic structure, we additionally conducted a fastSTRUCTURE analysis within each of these clades independently.

The identification of population clusters can be confounded by an underlying pattern of isolation-by-distance (IBD) and vice-versa (66). Therefore, we explored our dataset for a linear relationship between genetic distance (F_{ST}) and geographic distance between sites (input data provided in Table S25), which would indicate IBD. Specifically, we used partial Mantel tests to explore whether IBD patterns remained significant while correcting for clusters as a covariate, and whether clusters remained significant after correcting for geographic distance. Partial Mantel tests were carried out with the 'ncf' package v.1.2-8 (67) in R, using the "pearson" method. We defined clusters in two different ways: a) the main east-west split (i.e., two clusters) and b) the three clusters visible in PCoA and fastSTRUCTURE plots.

Finally, we explored the dataset for fixed allelic differences between populations (when no alleles are shared for a given SNP), which indicates a complete absence of contemporary gene flow (68). Whereas many clustering approaches assume HWE, fixed difference analysis is less affected by departures from HWE or small sample sizes (69). Using the 'gl.fixed.diff' function in dartR, we calculated the number of SNPs fixed for different alleles among populations. Code for all analyses performed in R can be found on Figshare at doi:10.26180/14446023.

xiv. Phylogeographic results for conservative SNP dataset

Table S23 Genetic diversity statistics for populations of *Palirhoeus eatoni* based on a conservative dataset of 5,363 SNPs. None of the populations were found to be significantly different with respect to any of these genetic diversity metrics, based on 5,000 bootstraps over loci. 95% confidence intervals are provided in parentheses.
H_O = observed heterozygosity; H_E = expected heterozygosity; F_{IS} = inbreeding coefficient.

Island	Site	H_O	H_E	F_{IS}
Heard	HD	0.037 (0.034, 0.039)	0.053 (0.049, 0.056)	0.309 (0.286, 0.331)
Kerguelen	KR-a	0.057 (0.053, 0.061)	0.065 (0.061, 0.069)	0.125 (0.096, 0.153)
	KR-b	0.076 (0.072, 0.080)	0.077 (0.073, 0.081)	0.014 (0, 0.030)
	KR-c	0.074 (0.070, 0.078)	0.079 (0.075, 0.084)	0.067 (0.045, 0.090)
Possession	PO-a	0.121 (0.117, 0.126)	0.150 (0.145, 0.155)	0.190 (0.178, 0.202)
	PO-b	0.117 (0.112, 0.121)	0.159 (0.154, 0.164)	0.268 (0.249, 0.286)
	PO-c	0.113 (0.108, 0.117)	0.142 (0.136, 0.147)	0.206 (0.187, 0.225)
Marion	MA	0.061 (0.057, 0.064)	0.071 (0.067, 0.075)	0.151 (0.137, 0.165)
Prince Edward	PE-a	0.067 (0.064, 0.071)	0.078 (0.074, 0.082)	0.131 (0.115, 0.147)
	PE-b	0.066 (0.062, 0.070)	0.075 (0.071, 0.079)	0.119 (0.103, 0.136)

Table S24 Matrix of pairwise F_{ST} values among sites for *Palirhoeus eatoni*, based on a conservative dataset of 5,363 SNPs. Site codes correspond to those provided in Table S14 (capital letters code for islands, lower-case suffixes for sites within islands).

	HD	KR-a	KR-b	KR-c	PO-a	PO-b	PO-c	MA	PE-a	PE-b
HD	-									
KR-a	0.453	-								
KR-b	0.298	0.256	-							
KR-c	0.318	0.361	0.172	-						
PO-a	0.561	0.468	0.516	0.479	-					
PO-b	0.645	0.518	0.579	0.530	0.093	-				
PO-c	0.668	0.542	0.603	0.566	0.144	0.071	-			
MA	0.890	0.884	0.877	0.877	0.816	0.845	0.852	-		
PE-a	0.890	0.878	0.870	0.867	0.788	0.820	0.829	0.048	-	
PE-b	0.894	0.882	0.873	0.871	0.788	0.821	0.831	0.028	0.047	-

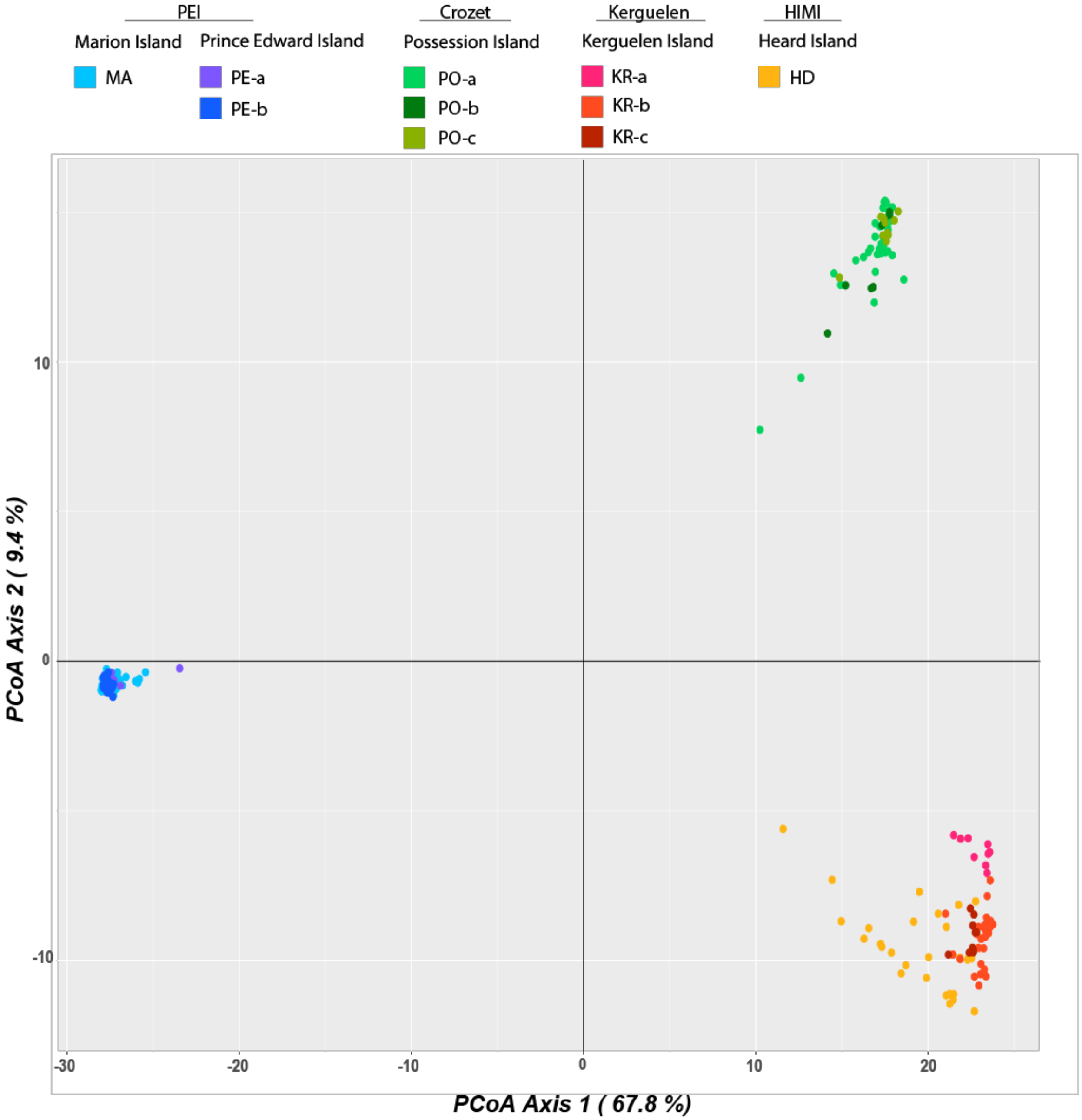

Figure S20 PCoA for *Palirhoeus eatoni* populations across the sub-Antarctic, using a conservative dataset of 5,363 SNPs. The first two principal coordinates are shown, which together explain 77% of genetic variation. Individual sites and the island and archipelago to which they belong are indicated in the legend.

xv. Input data for isolation-by-distance analysis

Table S25 Matrix of pairwise geodesic distances (in kilometers) between individual sites sampled for *Palirhoeus eatoni,* used as input data for isolation-by-distance analysis. Site codes correspond to those used in Table S14 (capital letters code for islands, lower-case suffixes for sites within islands). Distances were calculated using a coordinate-based distance calculator at *https://gps-coordinates.org/distance-between-coordinates.php*

	HD	KR-a	KR-b	KR-c	PO-a	PO-b	PO-c	MA	PE-a	PE-b
HD	-									
KR-a	453.92	-								
KR-b	468.57	26.58	-							
KR-c	489.44	45.78	21.80	-						
PO-a	1707.84	1423.92	1397.35	1380.22	-					
PO-b	1716.42	1432.56	1405.99	1388.68	8.67	-				
PO-c	1718.67	1435.75	1409.17	1392.08	12.21	4.93	-			
MA	2601.09	2414.34	2388.38	2374.54	1065.16	1057.90	1053.31	-		
PE-a	2605.21	2413.63	2387.60	2374.49	1056.61	1049.23	1044.69	28.40	-	
PE-b	2608.66	2417.65	2391.63	2377.62	1061.33	1053.96	1049.41	25.52	5.08	-

xvi. References for all Supplementary Information

1. R. Jeannel, Croisière du Bougainville aux îles australes françaises. *Mém. Mus. Natl. hist. nat.* **14**, 63–201 (1940).
2. G. Kuschel, S. L. Chown, Phylogeny and systematics of the *Ectemnorhinus*-group of genera (Insecta: Coleoptera). *Invertebr. Syst.* **9**, 841-863 (1995).
3. R. E. Kass, A. E. Raftery, Bayes Factors. *J. Am. Stat. Assoc.* **90**, 773-795 (1995).
4. M. Fourment *et al.*, 19 dubious ways to compute the marginal likelihood of a phylogenetic tree topology. *Syst. Biol.* **69**, 209-220 (2020).
5. J. R. Oaks, K. A. Cobb, V. N. Minin, A. D. Leaché, Marginal likelihoods in phylogenetics: a review of methods and applications. *Syst. Biol.* **68**, 681-697 (2019).
6. W. Xie, P. O. Lewis, Y. Fan, L. Kuo, M.-H. Chen, Improving marginal likelihood estimation for Bayesian phylogenetic model selection. *Syst. Biol.* **60**, 150-160 (2011).
7. J. J. N. Kitson, B. H. Warren, C. Thébaud, D. Strasberg, B. C. Emerson, Community assembly and diversification in a species-rich radiation of island weevils (Coleoptera: Cratopini). *J. Biogeogr.* **45**, 2016-2026 (2018).
8. T. Westerhold *et al.*, An astronomically dated record of Earth's climate and its predictability over the last 66 million years. *Science* **369**, 1383-1387 (2020).
9. G. T. W. McClelland *et al.*, Climate change leads to increasing population density and impacts of a key island invader. *Ecol. Appl.* **28**, 212-224 (2018).
10. S. L. Chown, S. W. Nicolson, *Insect Physiological Ecology. Mechanisms and Patterns* (Oxford University Press, New York, USA, 2004).
11. S. L. Chown, C. J. Klok, Water-balance characteristics respond to changes in body size in Subantarctic weevils. *Physiol. Biochem. Zool.* **76**, 634-643 (2003).
12. C. J. Klok, S. L. Chown, Temperature- and body mass-related variation in cyclic gas exchange characteristics and metabolic rate of seven weevil species: Broader implications. *J. Insect Physiol.* **51**, 789-801 (2005).
13. H. M. Lease, B. O. Wolf, Lipid content of terrestrial arthropods in relation to body size, phylogeny, ontogeny and sex. *Physiol. Entomol.* **36**, 29-38 (2011).
14. P. H. Boersch-Supan, L. R. Johnson, R. A. Phillips, S. J. Ryan, Surface temperatures of albatross eggs and nests. *Emu* **118**, 224-229 (2018).
15. P. Jouventin, H. Weimerskirch, Satellite tracking of Wandering albatrosses. *Nature* **343**, 746-748 (1990).
16. S. Shin *et al.*, Phylogenomic data yield new and robust insights into the phylogeny and evolution of weevils. *Mol. Biol. Evol.* **35**, 823-836 (2018).
17. J.-D. Chapelin-Viscardi, J.-F. Voisin, P. Ponel, N. Van der Putten, *Pachnobium dreuxi* n. g., n. sp., ses occurrences modernes et fossiles sur l'archipel Crozet (Coléoptère Curculionidae Ectemnorrhininae). *Ann. Soc. Entomol. Fr.* **46**, 125-131 (2010).
18. G. C. Grobler, L. Janse van Rensburg, A. D. S. Bastos, C. T. Chimimba, S. L. Chown, Molecular and morphometric assessment of the taxonomic status of *Ectemnorhinus* weevil species (Coleoptera: Curculionidae, Entiminae) from the sub-Antarctic Prince Edward Islands. *J. Zool. Syst. Evol. Res.* **44**, 200-211 (2006).

19. J.-F. Voisin, J.-D. Chapelin-Viscardi, P. Ronel, M. Rapp, "Les Coléoptères de la province de Kerguelen (îles subantarctiques de l'océan Indien)" in Faune de France, 99. (Fédération française des Sociétés de Sciences Naturelles, Paris, France, 2017).
20. R. Lanfear, B. Calcott, S. Y. W. Ho, S. Guindon, PartitionFinder: combined selection of partitioning schemes and substitution models for phylogenetic analyses. *Mol. Biol. Evol.* **29**, 1695-1701 (2012).
21. A. Stamatakis, RAxML version 8: a tool for phylogenetic analysis and post-analysis of large phylogenies. *Bioinformatics* **30**, 1312-1313 (2014).
22. Z. Yang, PAML 4: phylogenetic analysis by maximum likelihood. *Mol. Biol. Evol.* **24**, 1586-1591 (2007).
23. Y. Yu, S. R. Davis, C. Shih, D. Ren, H. Pang, The earliest fossil record of Belidae and its implications for the early evolution of Curculionoidea (Coleoptera). *J. Syst. Palaeontol.* **17**, 2105-2117 (2019).
24. A. Rambaut, A. J. Drummond, D. Xie, G. Baele, M. A. Suchard, Posterior summarization in Bayesian phylogenetics using Tracer 1.7. *Syst. Biol.* **67**, 901-904 (2018).
25. R Core Team, "R: A language and environment for statistical computing". (R Foundation for Statistical Computing, Vienna, Austria, 2017), http://www.R-project.org/
26. V. V. Zherikhin, V. G. Gratshev, Fossil Curculionoid beetles (Coleoptera: Curculionoidea) from the lower Cretaceous of northeastern Brazil. *Paleontol. J.* **38**, 58-68 (2004).
27. M. F. D. E. A. Santos, J. R. M. Mermudes, V. M. M. D. Fonseca, A specimen of Curculioninae (Curculionidae, Coleoptera) from the Lower Cretaceous, Araripe Basin, north-eastern Brazil. *Palaeontology* **54**, 807-814 (2011).
28. R. G. Oberprieler, R. S. Anderson, A. E. Marvaldi, "Curculionoidea Latreille, 1820: Introduction, Phylogeny" in Handbook of Zoology. Arthropoda: Insecta. Coleoptera, Beetles. Volume 3: Morphology and Systematics (Phytophaga), R. A. B. Leschen, R. G. Beutel, Eds. (Walter de Gruyter, Berlin, 2014), pp. 285-300.
29. R. J. Rayner, G. Kuschel, R. G. Oberprieler, Cretaceous weevils from southern Africa, with description of a new genus and species and phylogenetic and zoogeographical comments (Coleoptera: Curculionoidea). *Insect Syst. Evol.* **25**, 137-149 (1994).
30. G. M. Dlussky, D. J. Brothers, A. P. Rasnitsyn, The first Late Cretaceous ants (Hymenoptera: Formicidae) from southern Africa, with comments on the origin of the Myrmicinae. *Insect Syst. Evol.* **35**, 1-13 (2004).
31. R. J. Rayner *et al.*, Cretaceous fossils from the Orapa diamond mine. *Palaeontol. Africana* **33**, 55-65 (1997).
32. G. Kuschel, Un curculiónido del Cretáceo superior: primer insecto fósil de Chile. *Inv. Zool. Chilenas* **5**, 49-54 (1959).
33. Y.-C. Hong, *Amber Insects of China* (Beijing Scientific and Technological Press [in Chinese], Beijing, 2002).
34. J. Rheinheimer, Neue fossile Rüsselkäfer (Coleoptera: Curculionidae) aus dem Eozän des Baltischen Bernsteins und der Grube Messel bei Darmstadt. *Stutt. Beitr. Naturkd., B (Geol. Paläontol.)* **365**, 1-24 (2007).
35. N. Franz, G. Zhang, Three new species of entimine weevils in Early Miocene amber from the Dominican Republic (Coleoptera: Curculionidae). *Biodivers. Data J.* 10.3897/BDJ.5.e10469, e10469-e10469 (2017).

36. G. Poinar Jr, A. A. Legalov, Five new species from the subfamily Entiminae (Coleoptera: Curculionidae) in Dominican amber. *Palaeontol. Electron.* **20(23A)**, 1-13 (2017).
37. E. A. Marvaldi, G. M. Del Río, A. V. Pereyra, N. Rocamundi, A. A. Lanteri, A combined molecular and morphological approach to explore the higher phylogeny of Entimine weevils (Coleoptera: Curculionidae), with special reference to South American taxa. *Diversity* **10**, 95 (2018).
38. P. D. N. Hebert, E. H. Penton, J. M. Burns, D. H. Janzen, W. Hallwachs, Ten species in one: DNA barcoding reveals cryptic species in the neotropical skipper butterfly *Astraptes fulgerator*. *Proc. Natl. Acad. Sci. U.S.A.* **101**, 14812-14817 (2004).
39. T. G. Barraclough, J. E. Hogan, A. P. Vogler, Testing whether ecological factors promote cladogenesis in a group of tiger beetles (Coleoptera: Cicindelidae). *Proc. R. Soc. Lond., Ser. B: Biol. Sci.* **266**, 1061-1067 (1999).
40. J. J. Astrin, P. E. Stüben, Phylogeny in cryptic weevils: molecules, morphology and new genera of western Palaearctic Cryptorhynchinae (Coleoptera:Curculionidae). *Invertebr. Syst.* **22**, 503-522 (2008).
41. C.-G. Kim *et al.*, Pattern of morphological diversification in the *Leptocarabus* ground beetles (Coleoptera: Carabidae) as deduced from mitochondrial ND5 gene and nuclear 28S rDNA sequences. *Mol. Biol. Evol.* **17**, 137-145 (2000).
42. D. D. McKenna, B. D. Farrell, Molecular phylogenetics and evolution of host plant use in the Neotropical rolled leaf 'hispine' beetle genus *Cephaloleia* (Chevrolat) (Chrysomelidae: Cassidinae). *Mol. Phylogen. Evol.* **37**, 117-131 (2005).
43. B. B. Normark, B. H. Jordal, B. D. Farrell, Origin of a haplodiploid beetle lineage. *Proc. R. Soc. Lond., Ser. B: Biol. Sci.* **266**, 2253-2259 (1999).
44. X. Xia, DAMBE7: New and improved tools for data analysis in molecular biology and evolution. *Mol. Biol. Evol.* **35**, 1550-1552 (2018).
45. X. Xia, Z. Xie, M. Salemi, L. Chen, Y. Wang, An index of substitution saturation and its application. *Mol. Phylogen. Evol.* **26**, 1-7 (2003).
46. M. A. Miller, W. Pfeiffer, T. Schwartz, "Creating the CIPRES Science Gateway for inference of large phylogenetic trees" in *Proceedings of the Gateway Computing Environments Workshop (GCE)* (Institute of Electrical and Electronics Engineers, New Orleans, USA, 2010), pp 1-8.
47. R. Bouckaert *et al.*, BEAST 2.5: An advanced software platform for Bayesian evolutionary analysis. *PLoS Comp. Biol.* **15**, e1006650 (2019).
48. J. Heled, A. J. Drummond, Bayesian inference of species trees from multilocus data. *Mol. Biol. Evol.* **27**, 570-580 (2010).
49. R. R. Bouckaert, A. J. Drummond, bModelTest: Bayesian phylogenetic site model averaging and model comparison. *BMC Evol. Biol.* **17**, 42 (2017).
50. G. A. McCulloch, J. M. Waters, Phylogenetic divergence of island biotas: Molecular dates, extinction, and "relict" lineages. *Mol. Ecol.* **28**, 4354-4362 (2019).
51. I. A. N. McDougall, W. Verwoerd, L. U. C. Chevallier, K–Ar geochronology of Marion Island, Southern Ocean. *Geol. Mag.* **138**, 1-17 (2001).
52. M. Heads, Metapopulation vicariance explains old endemics on young volcanic islands. *Cladistics* **34**, 292-311 (2018).
53. C. A. Hipsley, J. Müller, Beyond fossil calibrations: realities of molecular clock practices in evolutionary biology. *Front. Genet.* **5**, 138 (2014).

54. A. Papadopoulou, I. Anastasiou, A. P. Vogler, Revisiting the insect mitochondrial molecular clock: the mid-Aegean trench calibration. *Mol. Biol. Evol.* **27**, 1659-1672 (2010).
55. J. Pons, I. Ribera, J. Bertranpetit, M. Balke, Nucleotide substitution rates for the full set of mitochondrial protein-coding genes in Coleoptera. *Mol. Phylogen. Evol.* **56**, 796-807 (2010).
56. R. J. Hijmans, raster: Geographic Data Analysis and Modeling. R package version 2.5-8, https://CRAN.R-project.org/package=raster (2016).
57. R. Bivand, C. Rundel, rgeos: Interface to Geometry Engine-Open Source (GEOS). R package version 0.3-26, https://cran.r-project.org/web/packages/rgeos/index.html (2017).
58. A. Kilian *et al.*, Diversity Arrays Technology: A generic genome profiling technology on open platforms. *Methods Mol. Biol.* **888**, 67-89 (2012).
59. C. Sansaloni *et al.*, Diversity Arrays Technology (DArT) and next-generation sequencing combined: genome-wide, high throughput, highly informative genotyping for molecular breeding of *Eucalyptus*. *BMC Proc.* **5**, P54 (2011).
60. R. J. Elshire *et al.*, A robust, simple genotyping-by-sequencing (GBS) approach for high diversity species. *PLoS ONE* **6**, e19379 (2011).
61. B. Gruber, P. J. Unmack, O. F. Berry, A. Georges, dartR : An R package to facilitate analysis of SNP data generated from reduced representation genome sequencing. *Mol. Ecol. Resour.* **18**, 691-699 (2018).
62. N. Díaz-Arce, H. Arrizabalaga, H. Murua, X. Irigoien, N. Rodríguez-Ezpeleta, RAD-seq derived genome-wide nuclear markers resolve the phylogeny of tunas. *Mol. Phylogen. Evol.* **102**, 202-207 (2016).
63. P. G. Meirmans, P. H. Van Tienderen, GENOTYPE and GENODIVE: two programs for the analysis of genetic diversity of asexual organisms. *Mol. Ecol. Notes* **4**, 792-794 (2004).
64. A. Raj, M. Stephens, J. K. Pritchard, fastSTRUCTURE: variational inference of population structure in large SNP data sets. *Genetics* **197**, 573-589 (2014).
65. N. A. Rosenberg, Distruct: a program for the graphical display of population structure. *Mol. Ecol. Notes* **4**, 137-138 (2004).
66. P. G. Meirmans, The trouble with isolation by distance. *Mol. Ecol.* **21**, 2839-2846 (2012).
67. O. N. Bjornstad, ncf: Spatial Covariance Functions. R package version 1.2-8, https://CRAN.R-project.org/package=ncf (2019).
68. M. Slatkin, Gene flow and the geographic structure of natural populations. *Science* **236**, 787-792 (1987).
69. A. Georges *et al.*, Genomewide SNP markers breathe new life into phylogeography and species delimitation for the problematic short-necked turtles (Chelidae: Emydura) of eastern Australia. *Mol. Ecol.* **27**, 5195-5213 (2018).
